# Cingulate retinoic acid signaling regulates neuropathic pain and comorbid anxiodepression via extracellular matrix homeostasis

**DOI:** 10.1172/JCI190539

**Published:** 2025-07-01

**Authors:** Zhen-Zhen Li, Wan-Neng Liu, Ke-Xin Liu, Zhi-Wei Dou, Rui Zhao, Yun Chen, Meng-Meng Wang, Tao-Zhi Wang, Fei Wang, Wen-Juan Han, Wen-Guang Chu, Xing-Xing Zheng, Rou-Gang Xie, Hua Yuan, Xiao-Fan Jiang, Xiao-Long Sun, Ceng Luo, Sheng-Xi Wu

**Affiliations:** 1 Department of Neurobiology, School of Basic Medicine,; 2 Department of Neurosurgery,; 3 Department of Rehabilitation Medicine, and; 4 Department of Anesthesiology and Perioperative Medicine, Xijing Hospital, Fourth Military Medical University, Xi’an, China.

**Keywords:** Cell biology, Neuroscience, Extracellular matrix, Pain, Synapses

## Abstract

Neuropathic pain is often comorbid with affective disorders. Synaptic plasticity in anterior cingulate cortex (ACC) is assumed to be a crucial interface for pain perception and emotion. Laminin 1 (LAMB1), a key element of extracellular matrix (ECM) in ACC was recently revealed to convey extracellular alterations to intracellular synaptic plasticity and underlie neuropathic pain and aversive emotion. However, it remains elusive what triggers activity-dependent changes of LAMB1 and ECM remodeling after nerve injury. Here, we uncovered a key role of retinoic acid (RA)/RA receptor β (RARB) signaling in neuropathic pain and associated anxiodepression via regulation of ECM homeostasis. We showed that nerve injury reduced RA levels in the serum and ACC in mice and humans, which brought about downregulation of RA’s corresponding receptor, RARB. Overexpressing RARB relieved pain hypersensitivity and comorbid anxiodepression, while silencing RARB exacerbated pain sensitivity and induced anxiodepression. Further mechanistic analysis revealed that RARB maintained ECM homeostasis via transcriptional regulation of LAMB1, reversing abnormal synaptic plasticity and eventually improving neuropathic pain and aversive emotion. Taken together with our previous study, we revealed an intracellular-extracellular-intracellular feed-forward regulatory network in modulating pain plasticity. Moreover, we identified cingulate RA/RARB signaling as a promising therapeutic target for treatment of neuropathic pain and associated anxiodepression.

## Abstract 2

Cingulate retinoic acid signaling regulates neuropathic pain and comorbid anxiodepression via regulation of extracellular matrix homeostasis

## Graphical abstract

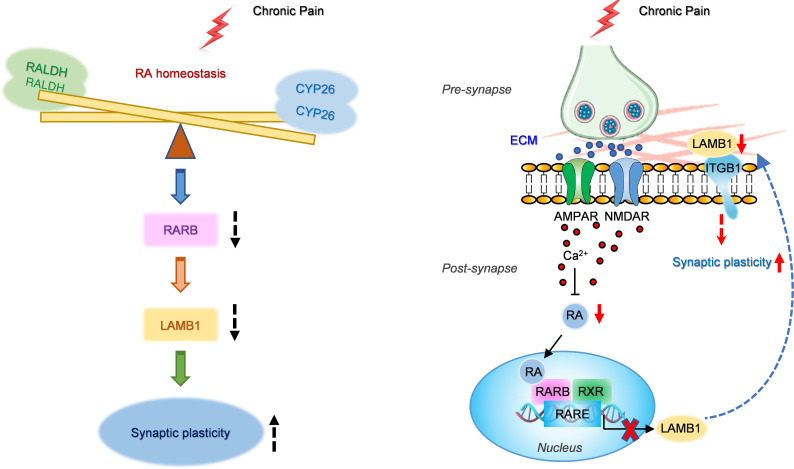

## Introduction

Chronic neuropathic pain frequently leads to emotional disturbances, such as anxiety and depression, which in turn exacerbates the severity and prolongs the duration of pain. This results in a vicious cycle between pain and anxiodepression, rendering neuropathic pain more intractable and resistant to traditional analgesics (1). Thus, finding a new and effective treatment for the comorbidity of neuropathic pain and affective disorders remains a major challenge (1).

Mounting evidence has documented the key significance of anterior cingulate cortex (ACC) as a critical interface for pain perception and emotional response (2–4). Following nerve injury, ACC neurons get activated, and inhibition of cingulate plasticity produces analgesic, anxiolytic, and antidepressive effects (5–9). Despite these advances, much attention thus far has been given to intracellular mechanisms of cingulate plasticity rather than extracellular alterations that might trigger and promote intracellular changes (10). Interestingly, we recently demonstrated an activity-dependent remodeling of extracellular matrix (ECM) in the ACC after nerve injury and revealed a new mechanism by which a key element of ECM, laminin 1 (LAMB1), conveys extracellular alterations to intracellular structural and functional plasticity and thus underlies neuropathic pain and anxiodepressive consequences (11). However, it remains elusive what triggers activity-dependent changes of LAMB1 and further ECM remodeling after nerve injury. Which signaling cascades are involved in this process?

It has shown that LAMB1 is mainly expressed in neurons and then secreted into extracellular space in the ACC (11). This suggests that an upstream intracellular cascade might be involved in triggering activity-dependent changes of ECM LAMB1 after nerve injury. In several murine and human cell lines, a retinoic acid response element (RARE) has been identified within the 5′-flanking region of the *Lamb1* gene (12). As a nuclear receptor superfamily, retinoic acid receptors (RARs), consisting of α, β, and γ subunits (13), function as transcription factors by binding to the RARE in the promoters of target genes, which is involved in neuronal development and synaptic plasticity homeostasis, ultimately affecting multiple brain functions (13–16). For example, among RAR subtypes, RARB shows preferential binding to the RARE of *Lamb1* promoter and triggers *Lamb1* transcription in murine cell lines (17). In adult mouse brain, RARB can modulate social cognition and spatial memory by regulating long-term potentiation (LTP) in the hippocampus (18, 19). However, very little is known about whether and how RARB regulates transcription of LAMB1 in the ACC and thus contributes to neuropathic pain and comorbid anxiodepression.

It is well known that the expression level and activity of RARB are regulated by its ligand retinoic acid (RA) (13, 14, 20). RA metabolic disturbance has been linked with affective disorders in clinical trials (21–23). As a metabolic product of retinol (vitamin A), the synthesis and metabolism of RA are strictly regulated in temporal and spatial dimension, thus controlling rational distribution of RA (24–26). Retinaldehyde dehydrogenase acts to catalyze the retinol into biologically active RA, while CYP26 (cytochrome P450 family 26), a metabolic enzyme, leads to the oxidization of RA (27, 28). Early studies of vitamin A and RA mainly focused on the eye, skin, and immune and reproductive systems (28, 29). Nevertheless, emerging evidence has shown that controlled RA synthesis is essential for regulating homeostatic synaptic plasticity (30, 31). RA deficiency is closely associated with a variety of brain diseases, e.g., developmental impairment, affective disorders, and cognitive dysfunction (21, 23, 32, 33). However, it remains elusive whether RA metabolic homeostasis is disturbed during pain chronicity. If so, would it affect ECM remodeling via regulation of RARB in the ACC and ultimately exacerbate pain responses and related negative emotion?

Using multiple cutting-edge approaches, we uncovered a key role of cingulate RA/RARB signaling in neuropathic pain and associated anxiodepression via regulation of ECM homeostasis. Following nerve injury, RA was significantly reduced in the serum and ACC of mice and humans. This caused the downregulation of its corresponding receptor, RARB. Overexpression of RARB relieved pain hypersensitivity and comorbid anxiodepression, while knockdown of RARB exacerbated pain sensitivity and induced anxiodepression. Further mechanistic analysis revealed that RARB acts to maintain ECM homeostasis via regulation of LAMB1 transcription, stabilizing the abnormal structural and functional plasticity of pyramidal neurons and eventually producing analgesic, anxiolytic, and antidepressive effects. This study sheds new light on the functional capability of RA/RARB homeostasis in modulating neuropathic pain and associated anxiodepression via interaction with ECM LAMB1. Taken together with our previous study (11), we revealed an intracellular-extracellular-intracellular feed-forward regulatory network underlying the comorbidity of neuropathic pain and anxiodepression. Moreover, we have identified cingulate RA/RARB signaling as a promising therapeutic target for treatment of neuropathic pain and associated anxiodepression.

## Results

### RARB as a transcription factor is decreased in the ACC after peripheral neuropathy.

**Figure 1:**
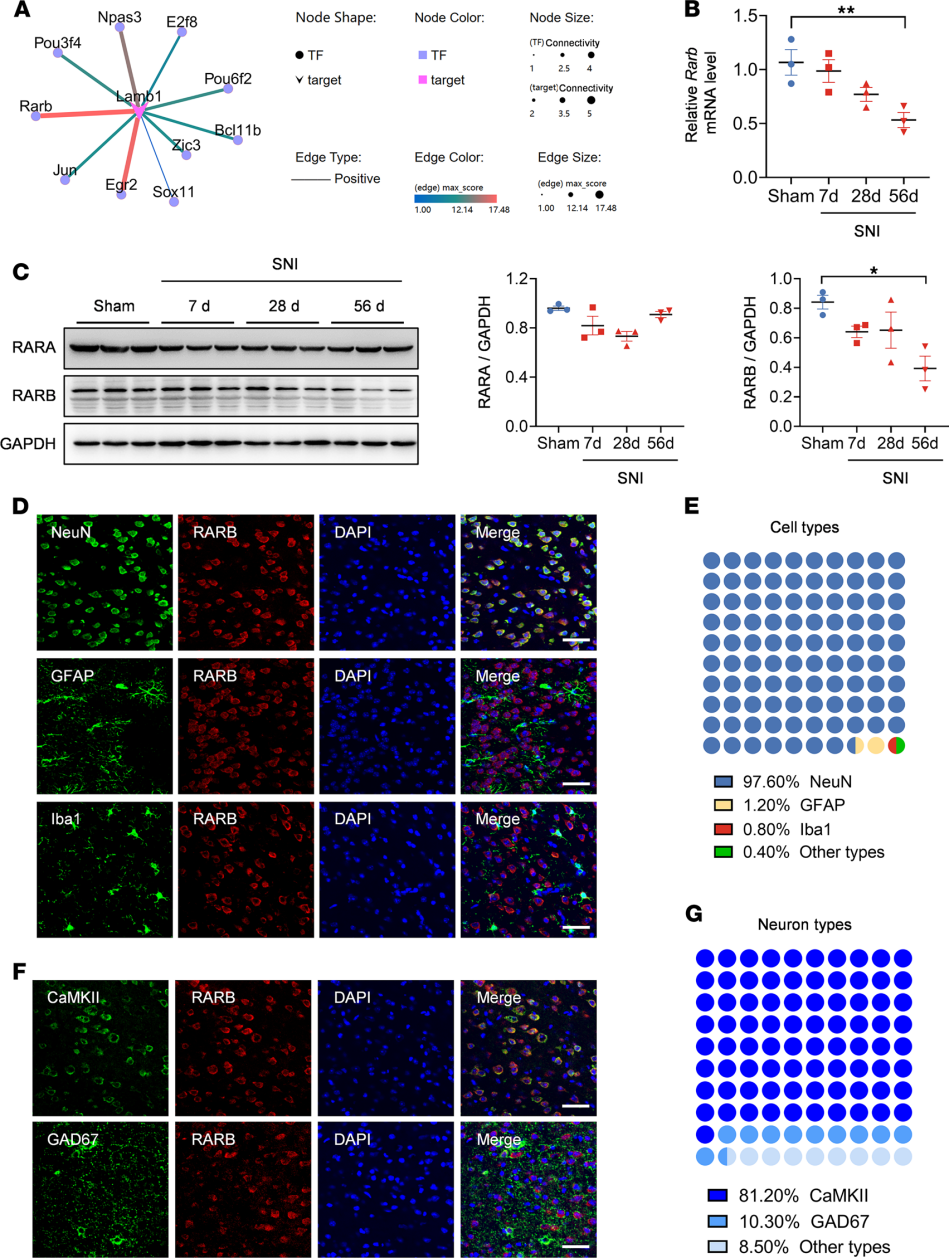
Peripheral neuropathy decreases RARB expression in the ACC. (**A**) Potential transcription factors (TF) of *Lamb1* with differentially expressed genes in RNA-Seq data of contralateral ACC from SNI-treated versus sham-treated mice. The line color and size indicate the relativity with *Lamb1* (*n* = 3–4 mice per group). (**B** and **C**) RARB expression in the ACC after SNI surgery at both the mRNA (**B**) (*n* = 3) and protein level (**C**) (*n* = 3). (**D** and **E**) Representative examples (**D**) and quantitative summary (**E**) of RARB coexpressing with neuronal nuclear antigen (NeuN), glial fibrillary acidic protein (GFAP), or ionized calcium–binding adapter molecule 1 (Iba1) (*n* = 3). (**F** and **G**) Representative examples (**F**) and quantitative summary (**G**) of RARB coexpressing with CaMKII or GAD67 neurons (*n* = 3). Scale bars: 30 μm in **D** and **F**. *P < 0.05, **P < 0.01. Statistical analysis was performed by 1-way ANOVA (**B** and **C** for RARB/GAPDH) and Kruskal-Wallis *H* test (**C** for RARA/GAPDH).

As we described previously (11), following a long-lasting pain hypersensitivity and comorbid anxiodepression caused by spared nerve injury (SNI), LAMB1, a key element of ECM, was significantly downregulated. To identify the potential transcription factors of LAMB1 that are involved in the process of neuropathic pain and associated anxiodepression, we analyzed the differentially expressed genes from RNA-Seq data in the ACC on day 56 after SNI. We identified 10 differential changes of transcription factors related with *Lamb1* (Figure 1A). Among which, RARB showed stronger relation with *Lamb1* and was involved in several physiopathological processes (12). We further verified differences at mRNA and protein levels by quantitative real-time PCR and immunoblotting in contralateral ACC at different time points after SNI, with significant downregulation of RARB on day 56 following SNI (Figure 1, B and C). We then characterized the expression profile of RARB in the ACC. The data revealed that RARB is highly coexpressed with neuronal nuclear antigen and sparsely with either glial fibrillary acidic protein or ionized calcium–binding adapter molecule 1 (Figure 1, D and E). Furthermore, we detected preferential RARB expression in calcium/calmodulin-dependent protein kinase II–positive (CaMKII-positive) neurons (Figure 1, F and G). Together, these data suggest a potential relationship between RARB and neuropathic pain as well as pain-related anxiodepression.

### RARB overexpression in the ACC relieves pain hypersensitivity and anxiodepression caused by nerve injury.

**Figure 2:**
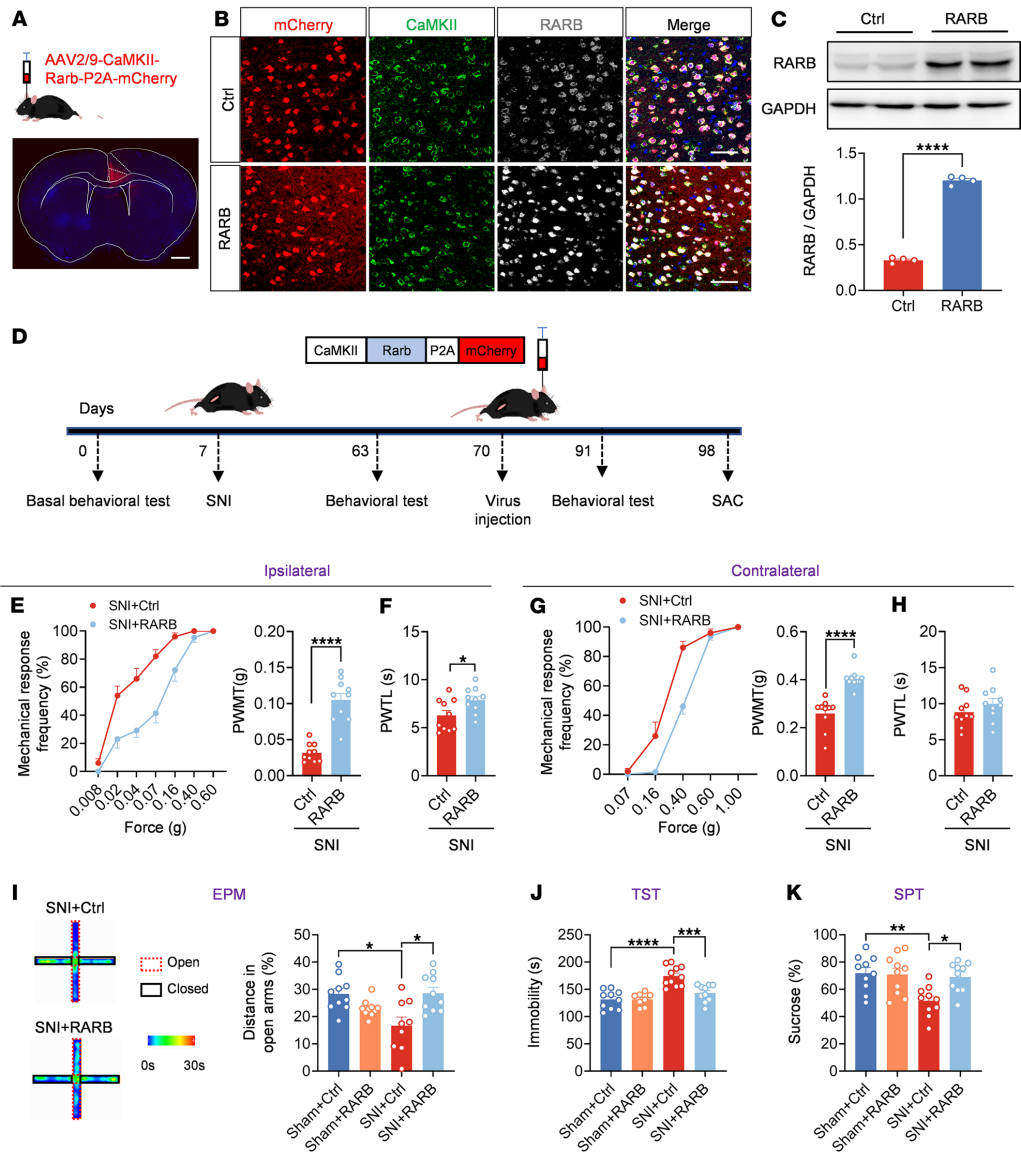
RARB overexpression in ACC relieves pain hypersensitivity and anxiodepression. (**A**) Schematic diagram showing intra-ACC virus injection. Scale bar: 1 mm. (**B** and **C**) Double immunofluorescence (**B**) and Western blotting (**C**) showing efficient RARB overexpression in ACC (*n* = 4). Scale bars: 30 μm in **B**. (**D**) Experimental schematic diagram showing virus injection in ACC and behavioral test. (**E** and **F**) Ipsilateral stimulus-response curve and mechanical threshold (**E**), and thermal sensitivity (**F**) in SNI-treated mice after ACC RARB overexpression (*n* = 10). (**G** and **H**) Contralateral stimulus-response curve and mechanical threshold (**G**), and thermal sensitivity (**H**) in SNI-treated mice after ACC RARB overexpression (*n* = 10). (**I**) Traveling trajectory in the EPM and quantitative summary of mice overexpressing RARB in the open arm (*n* = 9–10). (**J**) TST summary in mice after overexpression of RARB in the ACC (*n* = 8–11). (**K**) SPT in RARB-overexpressing Sham- and SNI-treated mice (*n* = 10). *P < 0.05, **P < 0.01, ***P < 0.001, ****P < 0.0001. Statistical analysis was performed by 2-tailed unpaired *t* test (**C**, **F**, and **H**), Mann-Whitney *U* test (**E** and **G**), 1-way ANOVA (**J** and **K**), and Kruskal-Wallis *H* test (**I**). PWMT, paw withdrawal mechanical threshold; PWTL, paw withdrawal thermal latency.

To address whether there is a causal relationship between activity-dependent changes of RARB and neuropathic pain and related anxiodepression, we generated a recombinant adeno-associated virions of serotype 2/9 (AAV2/9) expressing mCherry-tagged murine Rarb cDNA (designated as AAV-RARB) under the CaMKII promoter. The AAV2/9 expressing mCherry only served as control. The efficiency of RARB overexpression in the ACC was verified (Figure 2, A–C). We then assessed how overexpression of RARB in contralateral ACC affects pain sensitivity and anxiodepression-like behaviors (Figure 2D). Compared with control mice, overexpressing RARB in the right ACC in SNI-treated mice significantly reduced bilateral mechanical and ipsilateral thermal sensitivity (Figure 2, E–H). In contrast, basal mechanical and thermal nociception in bilateral hind paws was unaltered by cingulate RARB overexpression (Supplemental Figure 1, A–D; supplemental material available online with this article; https://doi.org/10.1172/JCI190539DS1).

Neuropathic pain is frequently comorbid with aversive emotions (1). We next observed whether RARB in the ACC relieves neuropathic pain–related anxiety and depression. In the elevated plus maze (EPM) test, SNI-treated mice expressing RARB exhibited frequent traveling in the open arm compared with control mice (Figure 2I and Supplemental Figure 1E). In the tail suspension test (TST) and sucrose preference test (SPT), overexpression of cingulate RARB reversed the longer immobility and reduction of sucrose preference in SNI-treated mice (Figure 2, J and K). In sham-treated mice, overexpression of RARB in the ACC did not alter traveling distance in the open arm of the EPM paradigm, immobility in the TST paradigm, or sucrose consumption in the SPT paradigm (Figure 2, I–K). These behavioral results suggest that RARB supplementation in the ACC relieves pain hypersensitivity and associated anxiodepression induced by peripheral neuropathy.

### Overexpression of RARB normalizes the abnormal spine remodeling and potentiated synaptic transmission in ACC pyramidal neurons after nerve injury.

**Figure 3:**
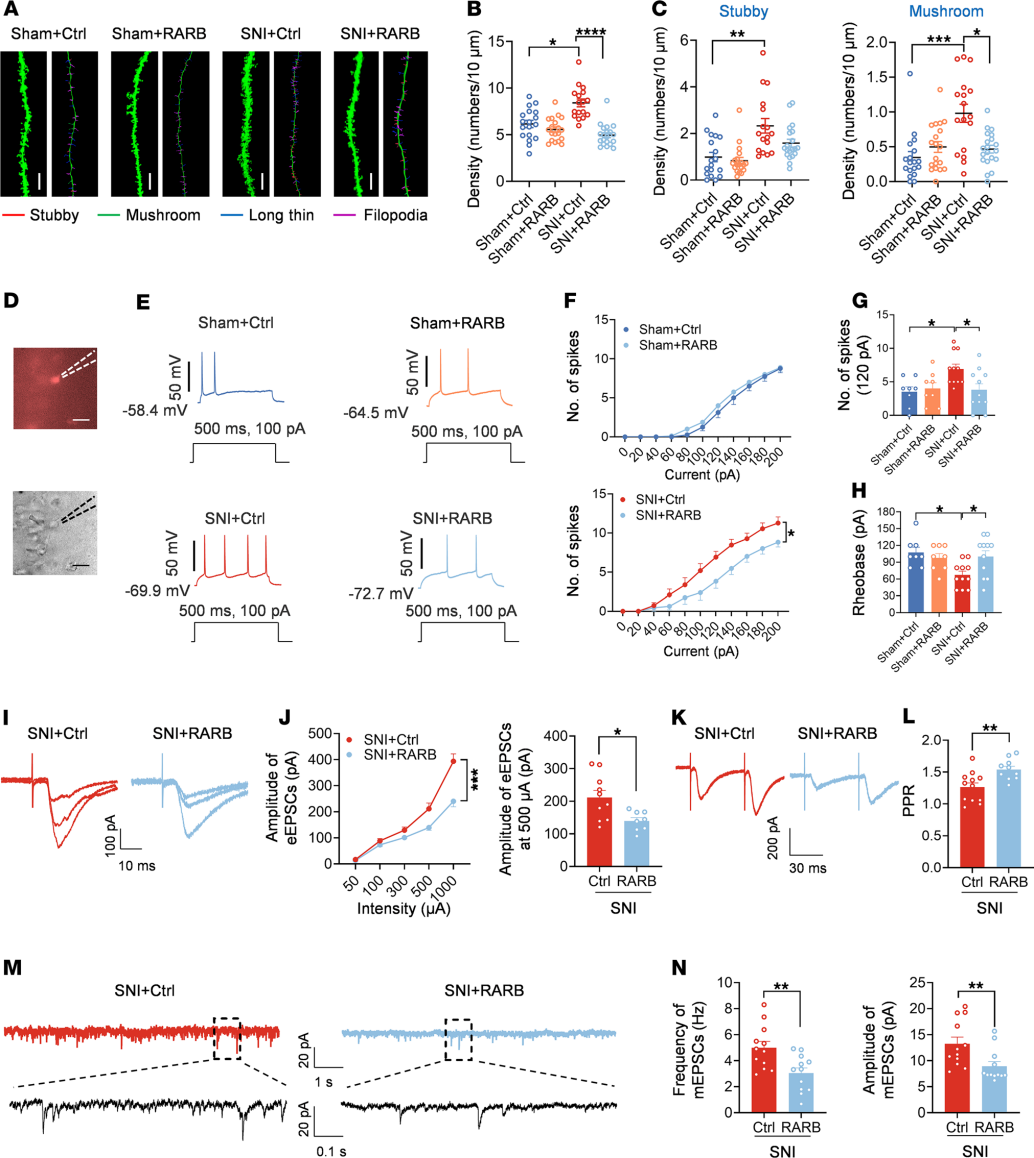
RARB overexpression in ACC normalizes abnormal structural and functional plasticity induced by SNI. (**A**) Representative images of apical dendrites of ACC pyramidal neurons obtained from mice overexpressing RARB and control virus in both sham and SNI conditions. Scale bar: 5 μm. (**B**) Summary of spine density on the apical dendrites in the above 4 conditions (*n* = 17–20). (**C**) Summary of the density of stubby and mushroom-shaped spines (*n* = 17–20). (**D**) Whole-cell patch-clamp recording from ACC layer II/III pyramidal neurons. Scale bar: 50 μm. (**E**) APs in neurons after overexpressing RARB in both genotypes of mice. (**F** and **G**) Input-output curve (**F**) and typical summary at intensity of 120 pA (**G**) after overexpressing RARB in sham- and SNI-treated mice (*n* = 8–11). (**H**) Rheobase after RARB overexpression in both types of mice (*n* = 8–11). (**I** and **J**) Representative traces (**I**) and input-output curve (**J**) of AMPAR-eEPSCs after overexpressing RARB in SNI-treated mice (*n* = 8–10). (**K** and **L**) Typical examples (**K**) and quantitative summary (**L**) of PPR of eEPSCs after RARB overexpression in the SNI condition (*n* = 10–12). (**M** and **N**) Representative traces (**M**), and mEPSC frequency and amplitude (**N**) after RARB overexpression in SNI-treated mice (*n* = 12). *P < 0.05, **P < 0.01, ***P < 0.001, ****P < 0.0001. Statistical analysis was performed by Kruskal-Wallis *H* test (**B** and **C**), 2-way ANOVA (**F** and left panel in **J**), 1-way ANOVA (**G** and **H**), 2-tailed unpaired *t* test (**L** and left panel in **N**), and Mann-Whitney *U* test (right panels in **J** and **N**).

Structural and functional synaptic plasticity in the ACC is assumed to be a cellular basis for the comorbidity of chronic pain and anxiodepression (2, 5, 8). Thus, we determined to examine whether supplementing RARB would normalize abnormal structural and functional changes in ACC pyramidal neurons after SNI. First, we examined the dendrite and spine structure of pyramidal neurons via Golgi staining in mice overexpressing RARB and mCherry alone. Sholl analysis revealed that the complexity of apical and basal dendrites of pyramidal neurons did not show obvious alterations after cingulate RARB overexpression in both sham and SNI conditions (Supplemental Figure 2, A–C). In contrast, overexpression of RARB eliminated the increase in the densities but not the length of total apical spines after SNI (Figure 3, A and B, and Supplemental Figure 2D). Further analysis of spine classification revealed that overexpression of RARB preferentially excluded the increased density of stubby and mushroom-shaped apical spines after SNI, with little influences on long, thin and filopodia-like apical spines (Figure 3C and Supplemental Figure 2E). Meanwhile, overexpression of RARB exerted similar effects on basal spines (Supplemental Figure 2, F–H). These results indicate that RARB contributes to the stabilization of synaptic spines in cingulate pyramidal neurons.

Next, we examined the functional influences of RARB on the intrinsic excitability and synaptic transmission in ACC pyramidal neurons using whole-cell patch-clamp recording (Figure 3D), which were identified by their morphological and firing properties (34). Passive membrane properties including resting membrane potential and membrane resistance as well as membrane capacitance of ACC pyramidal neurons were comparable between mice expressing RARB and mCherry alone in both sham and SNI conditions (Supplemental Figure 3A). However, the active membrane properties of ACC pyramidal neurons represented significant differences between 2 genotypes in SNI but not the sham condition (Figure 3, D–H). This was characterized by a reduced firing frequency and increased rheobase in pyramidal neurons of SNI-treated mice after overexpression of RARB in the ACC (Figure 3, D–H). The other parameters, such as action potential (AP) threshold, amplitude, and half-width, were unaltered by overexpression of cingulate RARB in both sham and SNI conditions (Supplemental Figure 3B).

Furthermore, we observed the depressed synaptic transmission in ACC pyramidal neurons derived from SNI-treated mice after overexpression of RARB. We recorded AMPA (α-amino-3-hydroxy-5-methyl-4-isoxazolepropionic) receptor–mediated evoked excitatory postsynaptic currents (AMPAR-eEPSCs) in pyramidal neurons from layers II/III in the ACC at a holding potential of –70 mV by applying local stimulation in layers V/VI in the presence of picrotoxin (100 μM), an antagonist of inhibitory synaptic transmission, and AP5 (50 μM), an antagonist of the NMDA receptor. The amplitude of AMPAR-eEPSCs was significantly reduced after overexpression of cingulate RARB (Figure 3, I and J). To elucidate whether a presynaptic or postsynaptic mechanism is involved, we first analyzed the paired-pulse ratio (PPR), i.e., EPSC2/EPSC1, a well-accepted indication of presynaptic mechanisms (35). Upon overexpression of cingulate RARB, the average amplitude of PPR was significantly increased in SNI-treated ACC pyramidal neurons, indicating a decrease in the transmitter release probability via a presynaptic mechanism (Figure 3, K and L). This was further confirmed by a decrease in miniature EPSC (mEPSC) frequency after overexpression of ACC RARB (Figure 3, M and N). In parallel, mEPSC amplitude was attenuated after supplementing RARB, indicating that a postsynaptic mechanism was involved as well (Figure 3, M and N). Overall, these results suggest that supplementation of cingulate RARB alleviates cingulate synaptic potentiation via both presynaptic and postsynaptic mechanisms.

### Overexpression of RARB reverses the exaggerated calcium transients in ACC pyramidal neurons after nerve injury.

**Figure 4:**
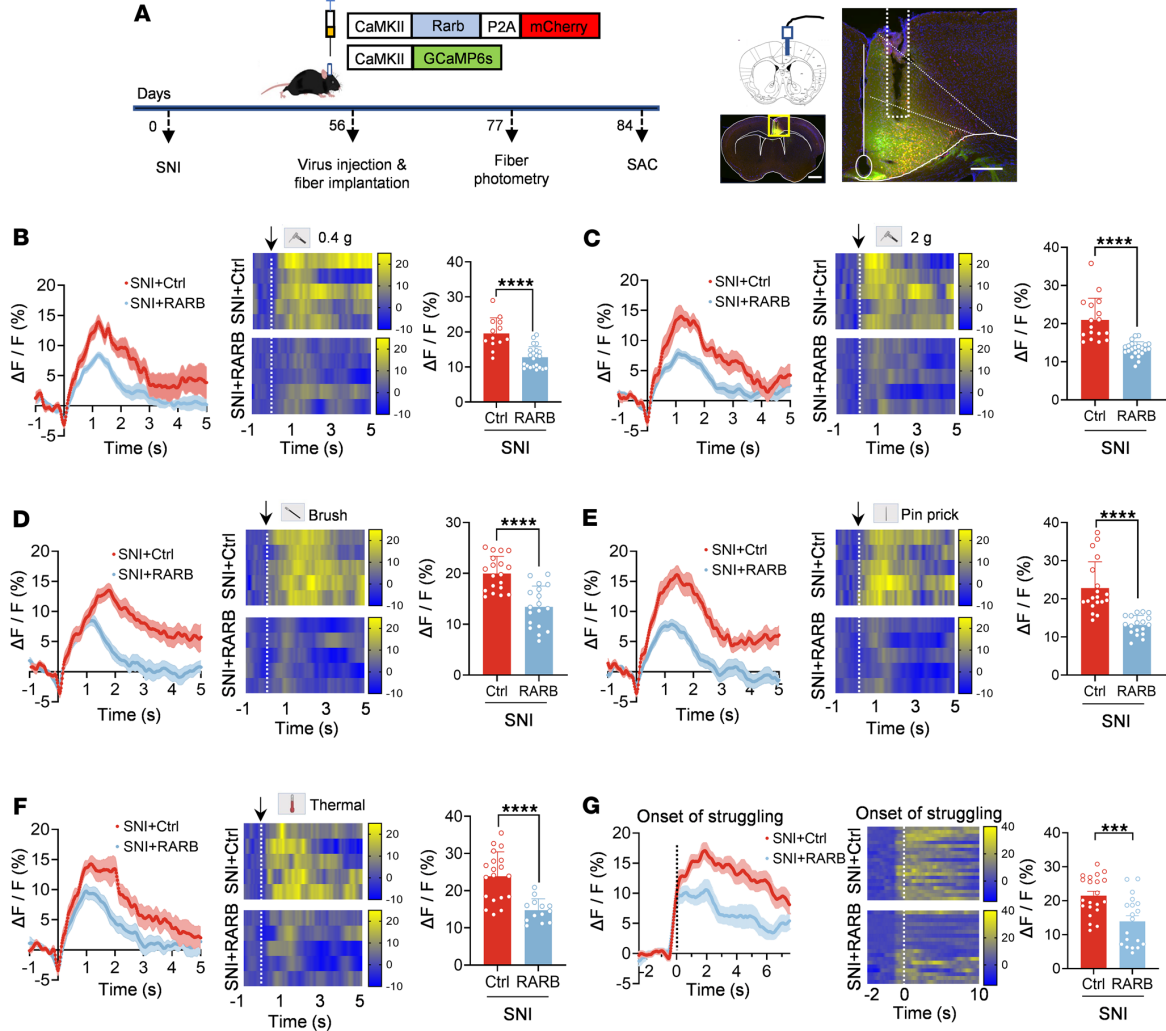
RARB overexpression in ACC alleviates neuronal hyperactivity induced by SNI. (**A**) Experimental schematic diagram showing virus injection, optical fiber implantation in the ACC, and fiber photometry recording during behavioral test in mice expressing control virus and RARB. SAC, sacrifice. Scale bar: 1 mm (left), 200 μm (right). (**B**–**G**) Representative photometry traces as shown in heat maps and quantitative summary from 5 independent experiments of peak GCaMP6s signals locked to the 0.4 g mechanical stimuli (**B**), 2 g mechanical stimuli (**C**), brush stimuli (**D**), pinprick nociception (**E**), and radiant heat stimulation (**F**) and the onset of struggling during tail suspension (**G**). ***P < 0.001, ****P < 0.0001. Statistical analysis was performed by Mann-Whitney *U* test (**B**, **C**, **E**, and **F**) and 2-tailed unpaired *t* test (**D** and **G**).

In further support of the crucial role of RARB in functional changes of ACC pyramidal neurons after nerve injury, we performed fiber photometry recording to monitor the activity of GCaMP6s-expressing pyramidal neurons in response to a wide range of external stimuli applied to a cutaneous receptive field and during tail suspension (Figure 4A). Overexpression of cingulate RARB significantly relieved the activity of ACC pyramidal neurons, as characterized by lower calcium transients evoked by peripheral mechanical stimuli, such as von Frey filaments, brush, and pinprick as well as by radiant heat stimuli in SNI-treated mice expressing RARB compared with control mice (Figure 4, B–F). Meanwhile, during tail suspension, we observed a reduced calcium transient in ACC pyramidal neurons after overexpression of RARB (Figure 4G). Taken together, we infer that overexpression of cingulate RARB contributes to normalizing the abnormal structural and functional plasticity of pyramidal neurons after peripheral neuropathy.

### RARB knockdown in the ACC induces pain hypersensitivity and anxiodepression.

**Figure 5:**
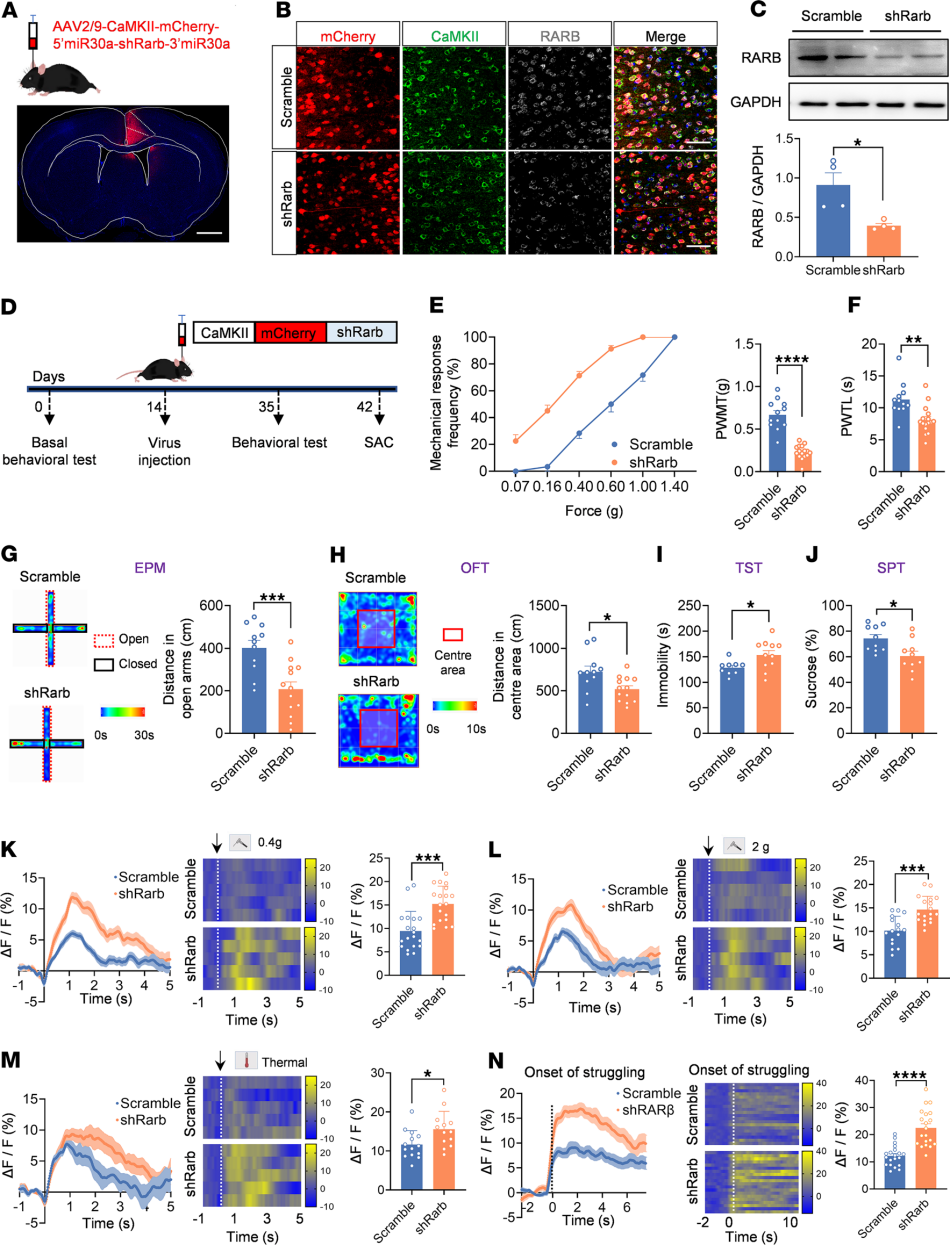
RARB knockdown in ACC induces pain hypersensitivity and anxiodepression. (**A**) Schematic diagram showing intra-ACC virus injection. Scale bar: 1 mm. (**B** and **C**) Double immunofluorescence (**B**) and Western blotting (**C**) showing efficient RARB knockdown (*n* = 4). Scale bars: 30 μm in **B**. (**D**) Schematic diagram showing virus injection in ACC and behavioral test in mice expressing scrambled shRNA and shRarb. (**E** and **F**) Ipsilateral stimulus-response curve and mechanical threshold (**E**), and thermal sensitivity (**F**) after ACC RARB knockdown in sham-treated mice (*n* = 12–16). (**G**) Traveling trajectory in the EPM and quantitative summary of sham-treated mice expressing shRarb in the open arm (*n* = 11–13). (**H**) Traveling trajectory in the OFT and quantitative summary of sham-treated mice expressing shRarb in the center area (*n* = 11–13). (**I**) TST after expression of AAV-shRarb in sham-treated mice (*n* = 9–11). (**J**) SPT in shRarb-expressing Sham-treated mice (*n* = 10). (**K**–**N**) Representative photometry traces as shown in heat maps and quantitative summary from 5 independent experiments of peak GCaMP6s signals locked to von Frey hair stimuli (0.4–2 g) (**K** and **L**), radiant heat stimuli (**M**), and the onset of struggling during tail suspension (**N**). *P < 0.05, **P < 0.01, ***P < 0.001, ****P < 0.0001. Statistical analysis was performed by Mann-Whitney *U* test (**C**, **E**, **I**, **K**, and **N**) and 2-tailed unpaired *t* test (**F**–**H**, **J**, **L**, and **M**).

To further address whether RARB is necessary and sufficient for neuropathic pain and related anxiodepression, we generated a recombinant AAV2/9 vector expressing an shRNA targeted against RARB (shRarb) and verified its knockdown efficiency (Figure 5, A–C). We first assessed how loss of unilateral cingulate RARB influences nociceptive sensitivity (Figure 5D). Compared with mice expressing scrambled shRNA, those expressing shRarb in the right ACC showed a greater response to von Frey hairs and thermal stimuli in the bilateral hind paw under the sham condition (Figure 5, E and F, and Supplemental Figure 4, A and B). We then examined whether exogenous knockdown of RARB causes pain-related aversive emotion. In EPM and open field test (OFT) paradigms, sham-treated mice expressing shRarb traveled a shorter distance than control mice in the open arm and center area (Figure 5, G and H, and Supplemental Figure 4, C and D). Meanwhile, mice expressing shRarb displayed longer immobility in the TST paradigm and less sucrose preference in the SPT paradigm compared with controls (Figure 5, I and J). Thus, these results further show that cingulate RARB negatively regulates pain sensitivity and related anxiodepression.

We further addressed whether cingulate RARB knockdown–evoked pain hypersensitivity and comorbid anxiodepression are associated with upregulated activity in ACC pyramidal neurons using in vivo fiber photometry recording. In the sham condition, GCaMP6s-expressing pyramidal neurons derived from mice expressing shRarb exhibited a higher calcium response in response to a wide range of mechanical stimuli, thermal stimuli, and tail suspension than those expressing scramble shRNA (Figure 5, K–N, and Supplemental Figure 4, E–G). These data further confirm that downregulation of RARB in the ACC leads to pain hypersensitivity and comorbid anxiodepression as well as exaggerated cingulate pyramidal neuronal activity.

### RARB in the ACC regulates ECM homeostasis via modulation of LAMB1 transcription after nerve injury.

**Figure 6:**
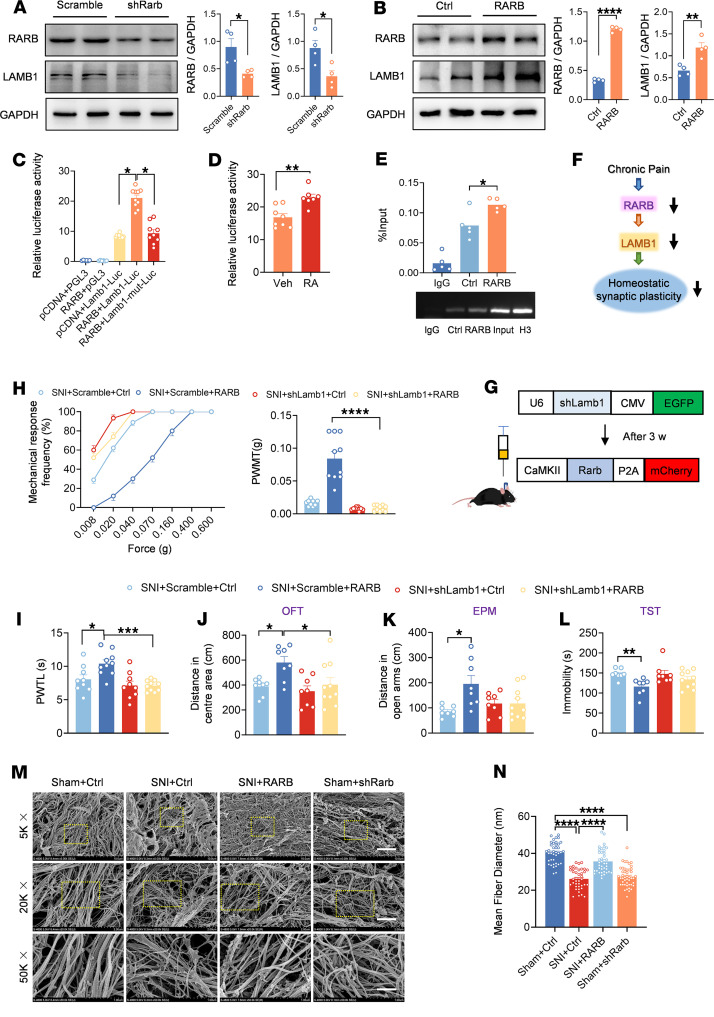
RARB regulates ECM remodeling via LAMB1 to modulate pain sensitivity and anxiodepression. (**A** and **B**) Representative immunoblots and quantitative summary of RARB and LAMB1 levels in ACC from mice expressing scrambled shRNA and shRarb (*n* = 4) (**A**) as well as control virus and RARB (*n* = 4) (**B**). (**C**) Luciferase activity after cotransfection of RARB-overexpressing plasmid and luciferase reporter plasmid connected with *Lamb1* promoter/*Lamb1* promoter mutant (*n* = 6–10). (**D**) Luciferase activity of vehicle and RA addition after cotransfection of RARB and *Lamb*1-Luc (*n* = 7–8). (**E**) ChIP assay of levels of RARB binding with the *Lamb1* promoter fragment in the ACC from mice expressing control (Ctrl) and RARB (*n* = 5). (**F**) A schematic model proposing the RARB regulatory mechanism in the process of chronic pain. (**G**) Schematic diagram showing virus injection in ACC. (**H** and **I**) Stimulus-response curve and mechanical threshold (**H**) and thermal hyperalgesia (**I**) in SNI-treated mice followed by shLamb1 and/or RARB overexpression treatment (*n* = 9–10). (**J**–**L**) OFT (**J**), EPM (**K**), and TST (**L**) in SNI-treated mice expressing shLamb1 and/or RARB (*n* = 8–10). (**M** and **N**) Representative scanning electron micrographs (**M**) and fiber diameter (**N**) in control mice, SNI-treated mice, SNI-treated mice overexpressing RARB, and sham-treated mice expressing shRarb (*n* = 3 mice per group). Scale bars: 5 μm (original magnification, ×5,000), 1.2 μm (original magnification, ×20,000), and 500 nm (original magnification, ×50,000). *P < 0.05, **P < 0.01, ***P < 0.001, ****P < 0.0001. Statistical analysis was performed by Mann-Whitney *U* test (**A** for RARB), 2-tailed unpaired *t* test (**A** for LAMB1, **B**, and **D**), Kruskal-Wallis *H* test (**C**, **H**, **K**, **L**, and **N**), and 1-way ANOVA (**E**, **I**, and **J**).

RARB functions as a potential transcription factor of LAMB1, a key component of ECM (12). Our recent study shed new light on the key significance of LAMB1 in chronic pain and comorbid anxiodepression (11). We were therefore interested to know whether RARB influences neuropathic pain and related aversive emotion via regulation of LAMB1 expression. First, we examined the changes in LAMB1 level after exogenous intervention of RARB expression in the ACC. Immunoblotting analysis revealed that knockdown or overexpression of cingulate RARB leads to downregulation or upregulation of LAMB1 correspondingly (Figure 6, A and B). Meanwhile, a luciferase assay indicated that overexpression of RARB significantly increased luciferase activity, which was modulated by the *Lamb1* promoter. A RARE has been identified within the –477 to –432 region of the *Lamb1* gene (12). We further constructed the specific mutant *Lamb1* promoter on the luciferase reporter and observed that luciferase activity was significantly decreased after cotransfection of RARB (Figure 6C). Administration of RA, a ligand of RARB, elevated luciferase activity after cotransfection of RARB and *Lamb1*-Luc plasmids (Figure 6D). In addition, a ChIP assay further confirmed that RARB binds to the *Lamb1* promoter, and the binding level was significantly increased after overexpressing RARB (Figure 6E). These data collectively suggest that RARB regulates the expression of LAMB1 as an upstream transcription factor. To further confirm the regulatory relationship between RARB and LAMB1 in neuropathic pain, we infected the ACC with AAV-shLamb1 and overexpressed RARB at an interval of 3 weeks in SNI-treated mice (Figure 6, F and G). We observed that knockdown of LAMB1 excludes the pain relief evoked by overexpression of RARB in SNI-treated mice (Figure 6, H and I). In parallel, the RARB-induced anxiolytic effect observed in the OFT paradigm was significantly inhibited in shLamb1-infected mice with SNI surgery (Figure 6J and Supplemental Figure 5F). A similar trend was observed in the EPM and TST paradigms, although it did not reach significance (Figure 6, K and L, and Supplemental Figure 5G). These results indicate that RARB regulates neuropathic pain and comorbid anxiodepression via modulation of LAMB1 transcription.

Structural changes of the ECM in the CNS are known to be associated with synaptic plasticity and various pathophysiological processes (10, 36). Given the role of LAMB1 as a key element of the ECM and a pivotal determinant in chronic pain and comorbid anxiodepression, we were interested in knowing whether this RARB-LAMB1 transcriptional interaction influences the abnormal ECM structural plasticity in the ACC after nerve injury. First, we assessed whether ECM abnormalities in the ACC occur in response to pain. Following nerve injury, WT mice displayed altered ECM microstructure in the ACC, manifesting as thinner and disordered fibers compared with sham controls (Figure 6, M and N). These ECM abnormalities in microstructure were normalized after overexpression of cingulate RARB (Figure 6, M and N), indicating a crucial role of RARB-LAMB1 signaling in maintaining the stability of ECM microstructure. In further support of this assumption, knockdown of cingulate RARB was shown to provoke the altered ECM microarchitecture in sham mice, which mimics the abnormal ECM structural alterations observed after nerve injury (Figure 6, M and N).

### RA levels are decreased in patients and mice with chronic pain and comorbid anxiodepression.

**Figure 7:**
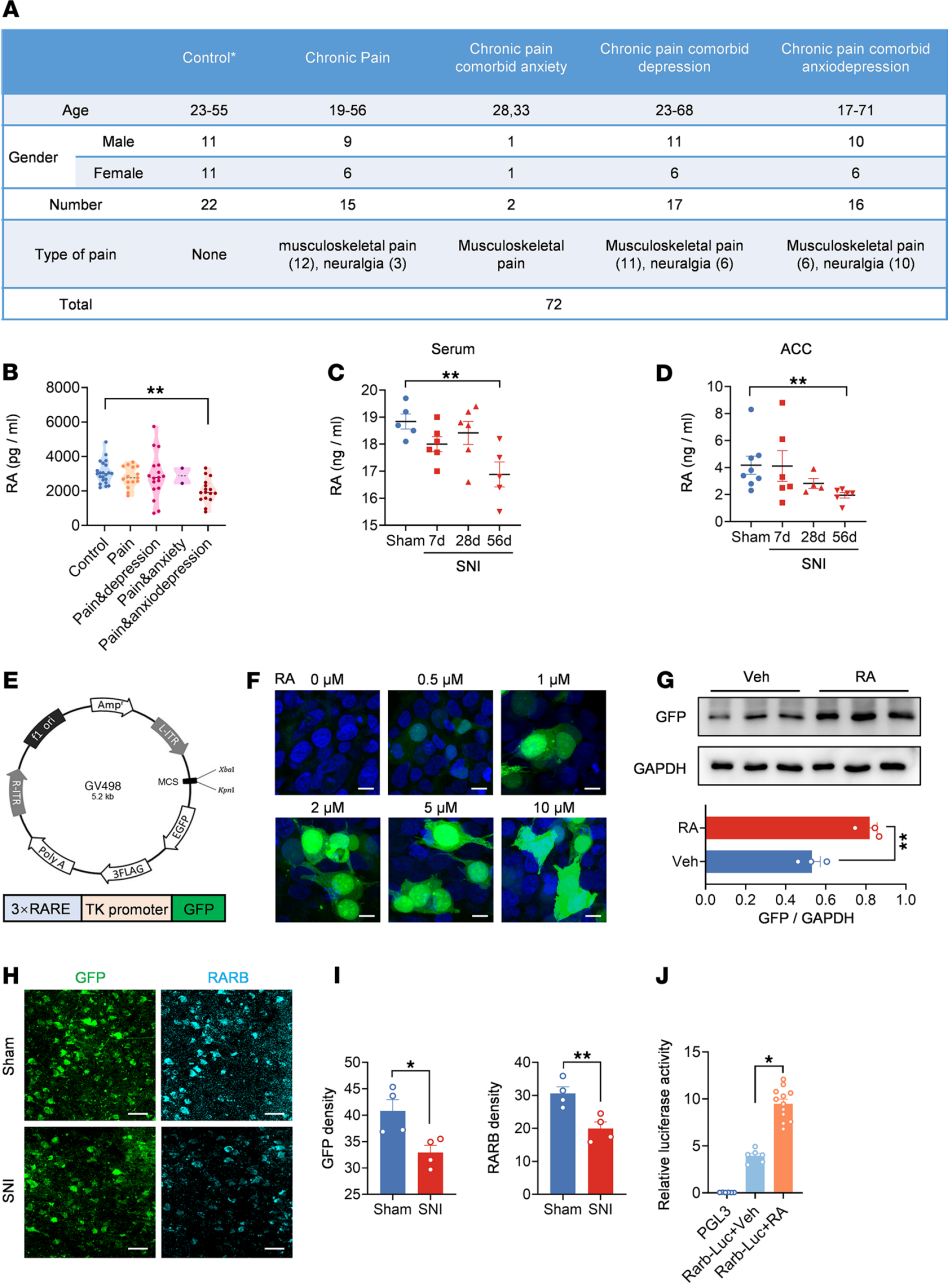
RA levels are decreased in chronic pain comorbid with anxiodepression. (**A**) A table summarizing the patient information. The groups met the following conditions: healthy volunteers: NRS < 3, GAD7 ≤ 4, HAMA ≤ 6, HAMD ≤ 8, PHQ-9 ≤ 4; chronic pain: NRS ≥ 3, GAD7 ≤ 4, HAMA ≤ 6, HAMD ≤ 8, PHQ-9 ≤ 4; chronic pain comorbid with anxiety: NRS ≥ 3, GAD7 > 4 and/or HAMA > 6, HAMD ≤ 8, PHQ-9 ≤ 4; chronic pain comorbid with depression: NRS ≥ 3, GAD7 ≤ 4, HAMA ≤ 6, HAM > 8, and/or PHQ-9 > 4; and chronic pain comorbid with anxiodepression: NRS ≥ 3, GAD7 > 4 and/or HAMA > 6, HAMD > 8 and/or PHQ9 > 4. (**B**) ELISA of RA level in serum from patients (*n* = 2–22). (**C** and **D**) ELISA of RA level in serum (**C**) (*n* = 5–6) and the ACC (**D**) (*n* = 4–8) after SNI surgery. (**E**) Schematic diagram showing construction of AAV2/9 expressing RARE–TK promoter–EGFP. TK, thymidine kinase. (**F**) Fluorescence images of GFP expression in 293FT cells transfected with AAV-RARE plasmid after RA treatment. Scale bars: 20 μm. (**G**) Immunoblots and quantitative summary of GFP expression in 293FT cells transfected with AAV-RARE plasmid after RA (5 μM) treatment (*n* = 3). Veh, vehicle. (**H** and **I**) Immunofluorescence (**H**) and quantitative summary (**I**) of GFP and RARB expression in ACC from SNI-treated mice expressing AAV-RARE (*n* = 4). Scale bars: 30 μm. (**J**) Luciferase activity of vehicle and RA addition in the transfection of *Rarb*-Luc (*n* = 6–12). *P < 0.05, **P < 0.01. Statistical analysis was performed by Kruskal-Wallis *H* test (**B**, **D**, and **J**), 1-way ANOVA (**C**), 2-tailed unpaired *t* test (**G** and **I** for RARB density), and Mann-Whitney *U* test (**I** for GFP density).

RA acts as an endogenous agonist for RARB, exerting a key role not only in CNS development, but also in regulating synaptic plasticity homeostasis (21–25, 32, 33, 37). Several clinical studies demonstrated the reduced serum RA levels in ischemic stroke patients comorbid with depressive symptoms (38–40), suggesting a negative correlation between RA levels and depressive comorbidities. Then, what changes occur in RA levels under chronic pain and comorbid anxiodepression? To address this question, we collected 72 blood samples from healthy volunteers and patients with chronic pain. All of them were assessed by a pain scale (numerical rating scale [NRS]), a depression scale (Patient Health Questionnaire-9 Items [PHQ-9] and Hamilton Depression Scale [HAMD]), and an anxiety scale (Generalized Anxiety Disorder-7 [GAD7] and Hamilton Anxiety Scale [HAMA]) and divided into different groups according to the assessment results (Figure 7A). Interestingly, the serum level of RA from patients with the comorbidity of pain and affective disorders was significantly decreased compared with the control group, while that from patients with only pain was not different from controls (Figure 7B). Consistently, a lower RA level was also observed in both the serum and ACC in mice at 56 d after SNI, when pain hypersensitivity and anxiodepression were fully established (Figure 7, C and D). These data indicate that RA metabolic disorder is closely related to anxiodepressive comorbidities associated with pain. To further visualize RA changes, we constructed a GFP-expressing AAV2/9 vector with RARE in the promoter, which could be activated by RA to express GFP for RA visualization (Figure 7E). At 24 h after transfection in 293FT cell lines, expression of GFP was stimulated upon application of increasing concentrations of RA (Figure 7F). Western blotting analysis verified the elevated GFP expression after RA challenge (Figure 7G). Three weeks after infection with AAV-RARE in the ACC of sham- and SNI-treated mice, immunofluorescence staining revealed that both GFP and RARB expression was significantly lowered after SNI treatment (Figure 7, H and I), further confirming the downregulated RA and RARB after nerve injury. Then, does RA directly regulate RARB expression? To address this question, we constructed a luciferase reporter plasmid containing the *Rarb* promoter. The luciferase assay indicated that addition of RA significantly increased luciferase activity after transfection of *Rarb-*Luc plasmid (Figure 7J). In support of our observation, a previous report has shown that RA specifically increases RARB mRNA level in human hepatoma cells (41). Taken together, these results suggest that RA homeostasis is imbalanced and directly affects RARB level in chronic pain state.

### Administration of RA relieves established pain hypersensitivity and anxiodepression after SNI.

**Figure 8:**
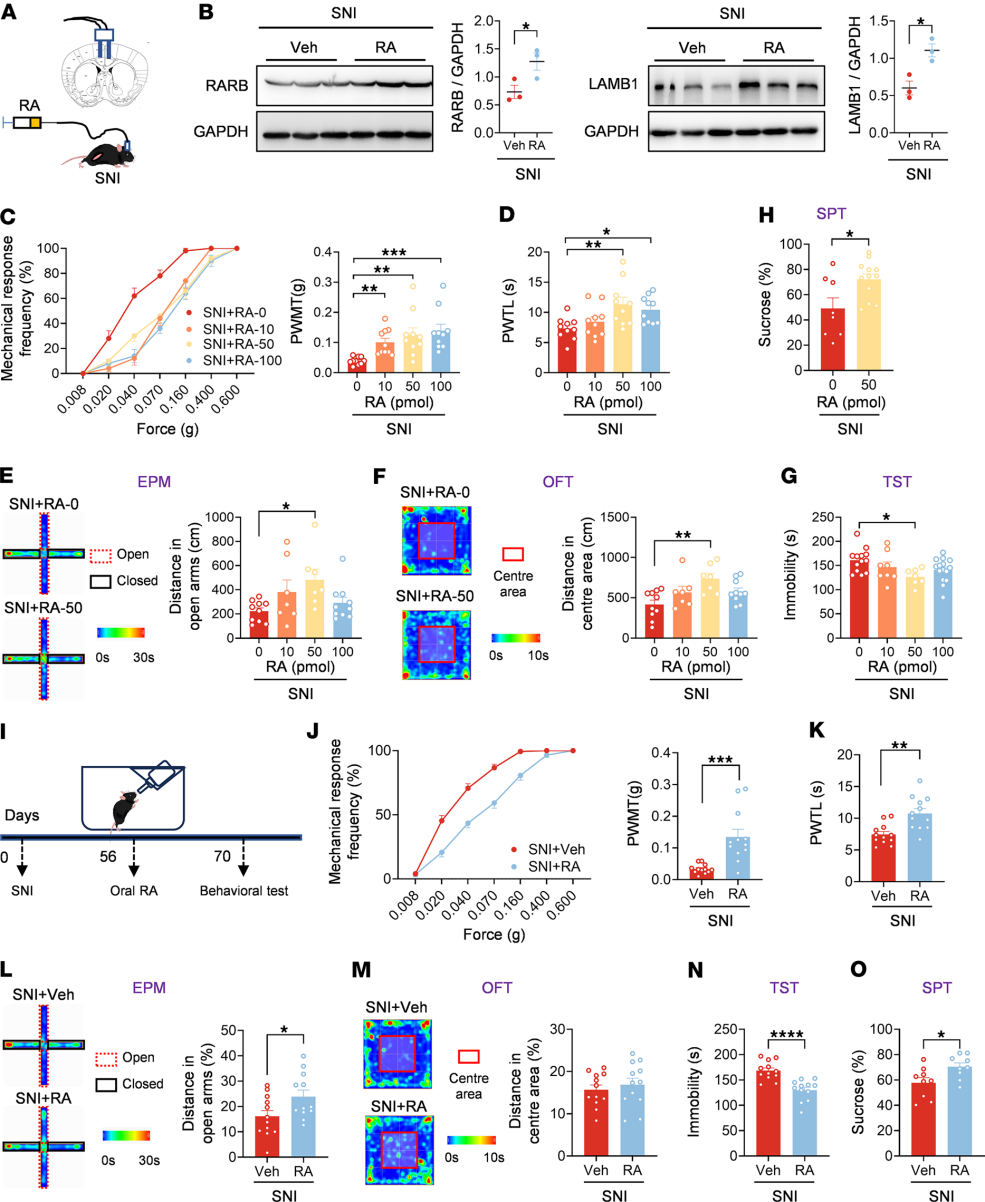
Administration of RA relieves established pain hypersensitivity and anxiodepression after SNI. (**A**) Schematic diagram of intra-ACC injection of RA in SNI-treated mice. (**B**) Immunoblots and quantitative summary of LAMB1 and RARB protein level after ACC injection of RA. Veh, vehicle. (**C** and **D**) Ipsilateral stimulus-response curves and mechanical threshold (**C**), and thermal latency (**D**) in SNI-treated mice followed by intra-ACC injection of RA (*n* = 10). (**E**) Open-arm exploring in the EPM test of SNI-treated mice after ACC delivery of RA (*n* = 7–10). (**F**) Center area exploring in the OFT of SNI-treated mice after ACC delivery of RA (*n* = 7–10). (**G** and **H**) TST (**G**) and SPT (**H**) in SNI-treated mice after intra-ACC injection of RA (*n* = 7–15). (**I**) Schematic diagram of oral intake of RA (0.6 mg/kg) in SNI-treated mice. (**J** and **K**) Ipsilateral stimulus-response curves and mechanical threshold (**J**), and thermal latency (**K**) in SNI-treated mice followed by oral intake of RA (*n* = 12). (**L**) Open-arm exploring in EPM test in SNI-treated mice after oral RA (*n* = 12). (**M**) Center area exploring in the OFT of SNI-treated mice after RA intake (*n* = 12). (**N** and **O**) TST (**N**) and SPT (**O**) of SNI-treated mice after RA intake (*n* = 9–12). *P < 0.05, **P < 0.01, ***P < 0.001, ****P < 0.0001. Statistical analysis was performed by 2-tailed unpaired *t* test (**B**, **H**, and **K**–**O**), Kruskal-Wallis *H* test (**C** and **E**), 1-way ANOVA (**D**, **F**, and **G**), and Mann-Whitney *U* test (**J**).

Finally, we asked whether RA as a kind of vitamin A metabolite has therapeutic effects on neuropathic pain and psychiatric comorbidity. We first delivered RA into ACC at 56 d after SNI (Figure 8A). Protein levels of LAMB1 and RARB were both upregulated after RA delivery in ACC (Figure 8B). Intracingulate RA delivery dose-dependently inhibited bilateral mechanical allodynia and ipsilateral thermal hyperalgesia induced by SNI (Figure 8, C and D, and Supplemental Figure 6, A–C). In addition, intra-ACC administration of RA increased open-arm exploration in the EPM, enhanced center area traveling in the OFT, decreased the immobility in the TST, and increased sucrose preference at a dose of 50 pmol (Figure 8, E–H, and Supplemental Figure 6, D and E), indicative of anxiolytic and antidepressive effects in the neuropathic state. In contrast, bilateral ACC administration of RA (50 pmol) neither altered basal nociception (Supplemental Figure 6, F–I) nor induced anxiety or depression-like behavior (Supplemental Figure 6, J–M).

Considering the potential clinical implication, we further sought to assess the potential analgesic effect of oral RA (Figure 8I). We found that oral intake of RA at 56 d after SNI significantly alleviated bilateral mechanical allodynia and thermal hyperalgesia (Figure 8, J and K, and Supplemental Figure 7, A–C). In addition, the reduced open arm exploring in the EPM, shortened traveling time in the OFT, elongated immobility in the TST, and reduced sucrose preference in the SPT were all normalized after oral RA delivery in SNI-treated mice (Figure 8, L–O, and Supplemental Figure 7, D and E). In contrast, in the basal state, oral administration of RA had no effect on mechanical and thermal threshold or on anxiodepressive behavior (Supplemental Figure 7, F–M). Overall, these results suggest that supplementation of RA via intracingulate injection or oral intake alleviates neuropathic pain and comorbid aversive emotion. Given the systematic effects of oral administration, oral intake of RA may exert analgesic, anxiolytic, and antidepressive effects via multiple sites in the CNS and PNS. Although we cannot exclude the possible roles of other central and peripheral sites, the essential role of the ACC in the beneficial effects of oral RA was established.

**Figure 9:**
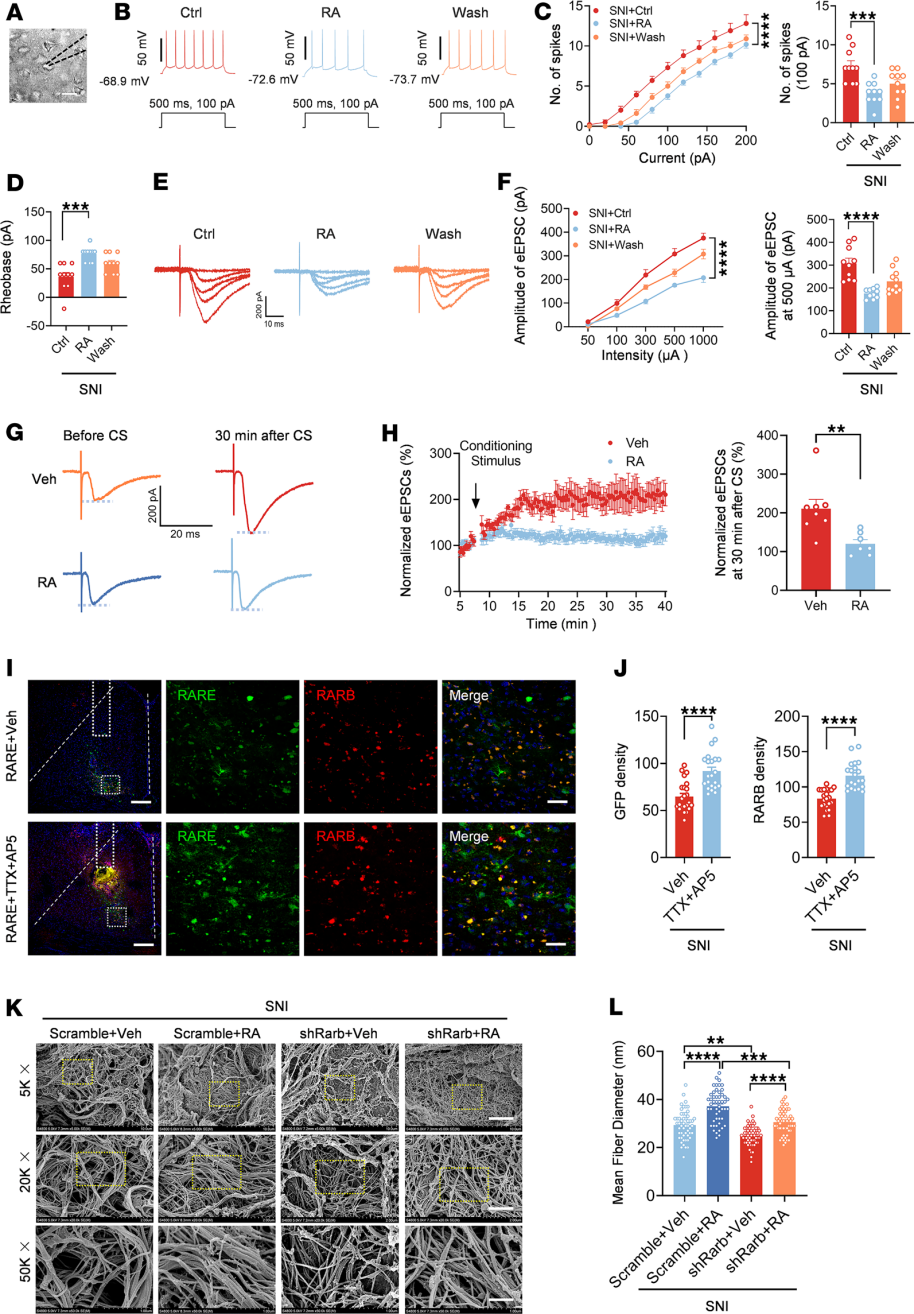
RA alleviates neuronal overexcitation by regulating ECM microstructure through RARB after SNI. (**A**) Whole-cell patch-clamp recording from ACC layer II/III pyramidal neurons. Scale bar: 50 μm. (**B** and **C**) APs at 100 pA (**B**) and input-output curve (**C**) after bath-applied RA (20 μM) (*n* = 10). (**D**) Rheobase of neurons after delivery of RA (*n* = 10). (**E** and **F**) Representative traces (**E**) and input-output curve (**F**) of AMPAR-eEPSCs in SNI-treated mice prior to, during, and after washout of RA (*n* = 10–11). (**G** and **H**) Representative traces (**G**), and time course and quantitative summary (**H**) of ACC LTP evoked by conditioning stimulus (CS) in the presence of RA (20 μM) and vehicle (Veh) (*n* = 7–8). (**I** and **J**) Confocal images (**I**) and quantitative summary (**J**) of GFP and RARB expression after delivery of TTX + AP5 in ACC from SNI-treated mice expressing AAV-RARE (*n* = 4). Scale bars: 200 μm (left), 30 μm (right). (**K** and **L**) Representative scanning electron micrographs (**K**) and quantitative summary (**L**) in SNI-treated mice with treatments at different magnification (*n* = 4 mice per group). Scale bars: 5 μm (original magnification, × 5,000), 1.2 μm (original magnification, ×20,000), and 500 nm (original magnification, ×50,000). **P < 0.01, ***P < 0.001, ****P < 0.0001. Statistical analysis was performed by 2-way ANOVA (left panels in **C** and **F**), 1-way ANOVA (right panel in **C**), Kruskal-Wallis *H* test (**D**, right panel in **F** and **L**), and 2-tailed unpaired *t* test (**H** and **J**).

We then addressed whether RA relieves neuropathic pain and related anxiodepression via regulating cingulate synaptic plasticity. Bath application of RA (20 μM) reversibly suppressed neuronal hyperexcitability and synaptic transmission of ACC pyramidal neurons in SNI-treated mice, as characterized by reduction of AP frequencies, elevation of AP rheobase, and decrease of AMPAR-eEPSCs (Figure 9, A–F). Furthermore, cingulate LTP induced by pairing training conditioning stimuli was normalized by RA delivery as well (Figure 9, G and H). These results suggest that RA alleviates abnormal synaptic plasticity. To further confirm whether RA level is modulated by neuronal activity, intra-ACC delivery of tetrodotoxin (TTX) (1 μM) and AP5 (100 μM) for 24 h was performed to block APs and NMDA receptors in SNI-treated mice expressing AAV-RARE. Immunofluorescence staining revealed that both GFP and RARB expression were significantly upregulated after administration of TTX and AP5 (Figure 9, I and J), suggesting the modulation of RA by neuronal activity. Taken together, we infer that RA significantly relieves pain and associated aversion by regulating cingulate synaptic potentiation. Next, we assessed whether RA regulates ECM microstructure via RARB. Following intra-ACC delivery of RA, ECM abnormalities in the microstructure after SNI were normalized (Figure 9, K and L). This normalization of dysregulated ECM homeostasis by RA was excluded after knockdown of cingulate RARB (Figure 9, K and L), indicating a vital role of RA in maintaining the stability of ECM microstructure by partially regulating RARB.

### Intervention of endogenous RA homeostasis regulates neuropathic pain and pain-related anxiodepression.

**Figure 10:**
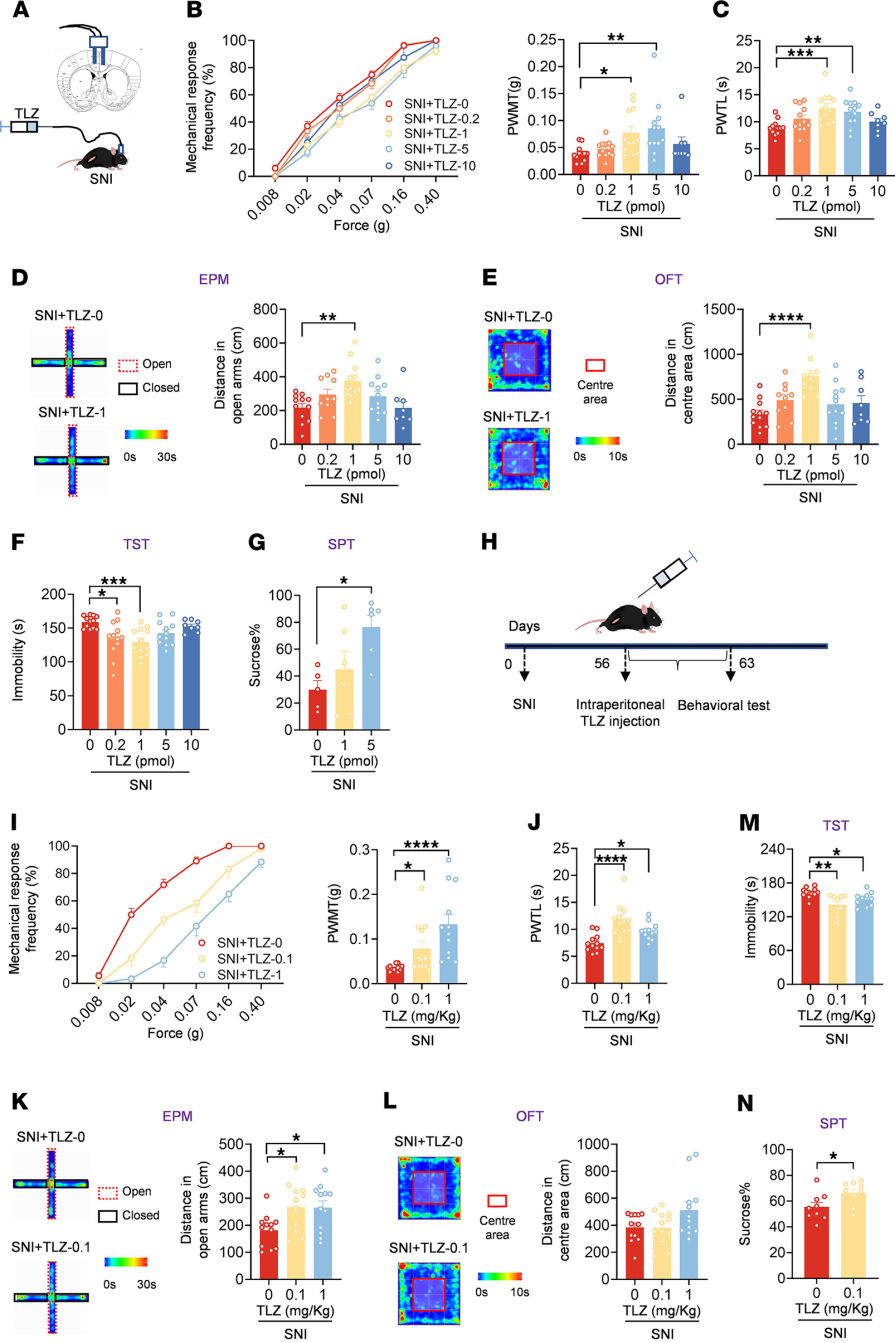
Administration of TLZ relieves established pain hypersensitivity and anxiodepression after SNI. (**A**) Schematic diagram of intra-ACC injection of TLZ in SNI-treated mice. (**B** and **C**) Ipsilateral stimulus-response curves and PWMT (**B**), and PWTL (**C**) in SNI-treated mice followed by intra-ACC injection of TLZ (*n* = 8–12). (**D**) Open-arm exploring in EPM test of SNI-treated mice after ACC delivery of TLZ (*n* = 8–12). (**E**) Center area exploring in the OFT of SNI-treated mice after ACC delivery of TLZ (*n* = 8–12). (**F** and **G**) TST (**F**) (*n* = 8–12) and SPT (**G**) (*n* = 5–6) in SNI-treated mice after intra-ACC injection of TLZ. (**H**) Schematic diagram of i.p. injection of TLZ in SNI-treated mice. (**I** and **J**) Ipsilateral stimulus-response curves and PWMT (**I**), and PWTL (**J**) in SNI-treated mice followed by administration of TLZ (*n* = 12). (**K**) Open-arm exploring in EPM test in SNI-treated mice after injection of TLZ (*n* = 12). (**L**) Traveling trajectory in the OFT and quantitative summary after TLZ injection in SNI-treated mice (*n* = 12). (**M** and **N**) TST (**M**) and SPT (**N**) in SNI-treated mice after i.p. TLZ (*n* = 9–12). *P < 0.05, **P < 0.01, ***P < 0.001, ****P < 0.0001. Statistical analysis was performed by Kruskal-Wallis *H* test (**B**, **F**, **I**, **L**, and **M**), 1-way ANOVA (**C**–**E**, **G**, **J**, and **K**), and 2-tailed unpaired *t* test (**N**).

RA maintains homeostasis through enzyme synthesis and metabolism. The RA-metabolizing enzyme CYP26 eliminates RA by hydroxylation of polar metabolites. Talarozole (TLZ), a specific CYP26 inhibitor, has become a potential therapeutic target in many fields due to its ability to block RA metabolism (27, 42, 43). We then asked whether modulation of endogenous of RA by TLZ alleviates neuropathic pain and related aversion. Intra-ACC delivery of TLZ dose-dependently relieved bilateral mechanical allodynia and ipsilateral thermal hyperalgesia at 56 d after SNI (Figure 10, A–C, and Supplemental Figure 8, A–C). Moreover, bilateral ACC injection of TLZ increased open-arm exploration in the EPM, center area traveling distance in the OFT, struggle time in the TST, and sucrose preference in SNI-treated mice (Figure 10, D–G, and Supplemental Figure 8, D and E), indicative of its desirable anxiolytic and antidepressive effects in the neuropathic state. In contrast, TLZ in the ACC rarely affected basal nociception and anxiodepressive behavior (Supplemental Figure 8, F–M). Furthermore, we adopted systemic administration of TLZ via i.p. injection (Figure 10H). In SNI-treated mice, i.p. TLZ significantly alleviated mechanical allodynia and thermal hyperalgesia as well as comorbid anxiodepression in a dose-dependent manner (Figure 10, I–N, and Supplemental Figure 9, A–E). In contrast, i.p. TLZ had no effect on the basal nociception (Supplemental Figure 9, F–I). In sum, these results indicate that intervention of endogenous RA metabolism relieves neuropathic pain and related anxiodepression.

For the safety profile study, mice were treated with oral RA for 2 consecutive weeks or i.p. TLZ for 7 consecutive days. The animals showed no changes of any liver transaminases (alanine transaminase [ALT] and aspartate transaminase [AST]) or biomarkers of renal dysfunction (blood urea nitrogen [BUN] and creatinine) (Supplemental Figure 10, A–D and F-I). H&E staining showed no obvious damage in the lung, liver, kidney, and heart of mice treated with chronic RA or TLZ, suggesting that RA and TLZ induced little toxicity in mice (Supplemental Figure 10, E and J).

As mentioned above, synthetase also plays an important role in RA homeostasis. As a key element of RA synthetase, ALDH1A2 is involved in various pathophysiological processes (27, 28, 44). We next generated AAV2/9 expressing ALDH1A2 to explore whether there is a causal relationship between ALDH1A2 and neuropathic pain and anxiodepressive consequences (Supplemental Figure 11A). Western blot analysis revealed that RARB and LAMB1 were both significantly upregulated after ALDH1A2 overexpression (Supplemental Figure 11B). Three weeks after infection of AAV2/9-ALDH1A2, SNI-induced mechanical allodynia and thermal hyperalgesia were significantly relieved compared with control virus (Supplemental Figure 11, C–H). Meanwhile, decreased sucrose preference in the SPT and struggle time in the TST after SNI were largely recovered after overexpressing ALDH1A2 (Supplemental Figure 11, K and L). However, SNI-induced anxiety-like behavior was rarely reversed after ALDH1A2 overexpression (Supplemental Figure 11, I and J). Altogether, we inferred that activation of RA synthetase exerts desirable analgesic and antidepressive effects in the neuropathic state, with little anxiolytic effect.

Finally, we determined whether RARB in ACC is involved in depression without chronic pain. We used 2 rodent models of depression: one involving chronic exposure to corticosterone (CORT) and the other involving chronic restraint stress (CRS). No significant changes in RARB expression were observed in the ACC after chronic CORT or CRS exposure (Supplemental Figure 12, A and B), suggesting specific involvement of ACC RARB in the development of chronic pain and associated depression but not in non–pain-related depression. Moreover, we found little change in ACC RARB levels in another chronic inflammatory pain model induced by unilateral hind paw injection of complete Freund’s adjuvant (CFA) (Supplemental Figure 12C). Additionally, the changes in ACC RARB levels following SNI were not sex specific, as female mice also showed the downregulated RARB in ACC after SNI (Supplemental Figure 12D). Overall, we conclude that activation of ACC RA/RARB signaling may represent a potential therapeutic target for treatment of neuropathic pain and related anxiodepression.

## Discussion

**Figure 11:**
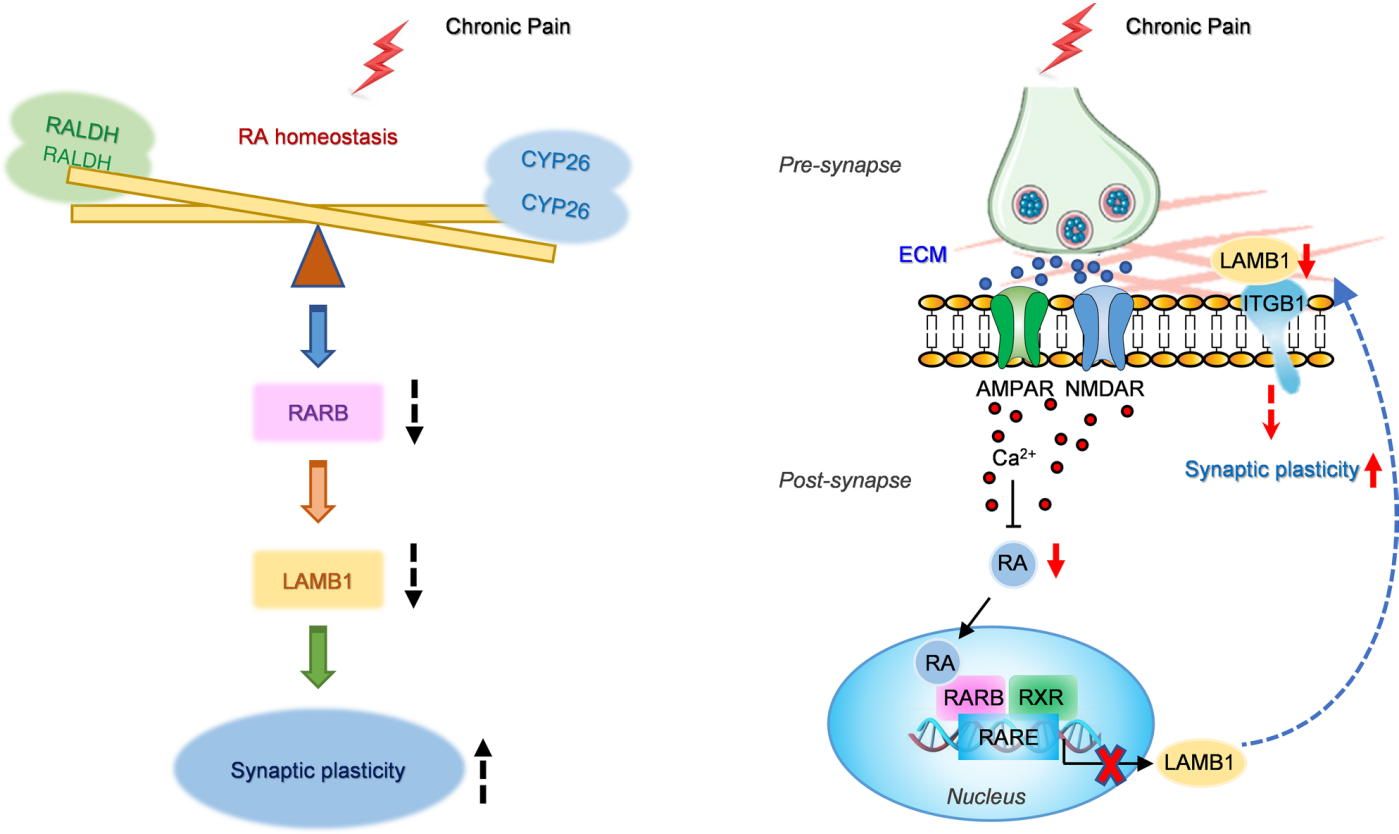
A schematic model proposing how cingulate RA/RARB homeostasis modulates neuropathic pain and associated anxiodepression via interaction with ECM LAMB1 through an intracellular-extracellular-intracellular feed-forward regulatory network. See Discussion for details.

The results of the present study led us to propose a working model, as schematically illustrated in Figure 11. Following nerve injury, enhanced neuronal activity in ACC pyramidal neurons disturbs RA metabolic homeostasis, causing the reduced RA in the ACC. This brings about the downregulation of its corresponding receptor, RARB, which causes a reduction in LAMB1 transcription. This in turn results in disturbance of ECM microstructure, which further leads to abnormal spine remodeling and synaptic potentiation of ACC pyramidal neurons, collectively contributing to exaggerated pain response and associated anxiodepression. In sum, this study primarily clarifies the role of RA in regulating ECM microstructure by acting on RARB. More importantly, these results infer that maintaining RA homeostasis may be a promising therapeutic strategy for treatment of neuropathic pain and related aversive emotion.

The most striking finding of this study was the identification of RARB, a RAR in the ACC, as a key intracellular upstream trigger for ECM remodeling in regulating neuropathic pain and related anxiodepression. Much progress has been made in elucidating the role of ACC in neuropathic pain and comorbid affective disorders, with a major focus on intracellular plasticity, but extracellular alterations have long been overlooked. It is only recently that we uncovered a new mechanism by which a key element of ECM, LAMB1, conveys extracellular alterations to intracellular structural and functional plasticity and thus underlies neuropathic pain and anxiodepressive consequences (11). However, it remains elusive what triggers activity-dependent changes of LAMB1 and further ECM remodeling after nerve injury. Using RNA-Seq, we detected 10 differential changes of transcription factors related with *Lamb1* in the ACC after SNI treatment. It is noteworthy that among which, RARB showed the strongest association with *Lamb1*. As a nuclear receptor superfamily, RARs, consisting of α, β, and γ subunits (13, 19), are assumed to function as transcription factors by binding to the RARE in the promoters of target genes, which are involved in neuronal development, synaptic plasticity, and homeostasis, ultimately affecting multiple brain functions, e.g., learning, memory, and affective cognition (13, 16, 18, 19, 45). In support of our observation, in vitro studies using murine and human cell lines identified a RARE in the 5′-flanking region of the *Lamb1* gene and reported a preferential binding of RARB to the RARE of the *Lamb1* promoter, triggering *Lamb1* gene transcription (12, 17). However, it remains unclear whether RARB regulates transcription of LAMB1 in the ACC and how this transcriptional interaction contributes to the development of neuropathic pain and comorbid anxiodepression. Consistent with downregulated LAMB1 as reported previously (11), we verified similar downregulation of RARB at transcriptional, mRNA, and protein levels after SNI. Western blotting analysis showed that overexpression or knockdown of RARB in the ACC correspondingly leads to upregulation or downregulation of LAMB1 expression. Luciferase and ChIP assays further demonstrated the transcription-promoting capacity of potential RARB-binding sites in *Lamb1* genes. These data suggest that RARB may act as an upstream transcription factor for LAMB1 in the ACC.

It is well known that structural changes of the ECM in the CNS are associated with synaptic plasticity and various pathophysiological processes (10, 36). Given the role of LAMB1 as a key element of the ECM and a pivotal determinant in chronic pain and comorbid anxiodepression, an interesting question arises regarding whether this RARB-LAMB1 signaling influences abnormal structural plasticity of the ECM in the ACC after nerve injury. In the present study, we demonstrated that nerve injury induces altered ECM microstructure in the ACC, which was normalized after overexpression of cingulate RARB, indicating a crucial role of RARB-LAMB1 signaling in maintaining the stability of the ECM microstructure.

To further determine the causal relationship between plastic changes of cingulate RARB and neuropathic pain, we overexpressed RARB in the ACC and found that supplementation of RARB significantly alleviated pain hypersensitivity and related anxiety and depression in SNI-treated mice. In contrast, knocking down cingulate RARB exaggerated pain sensitivity and induced anxiodepression in control mice. More importantly, the above actions of RARB were excluded by blockade of LAMB1. Thus, we inferred that RARB in the ACC may negatively regulate neuropathic pain and anxiodepressive consequences via regulation of LAMB1 transcription. Additionally, we found no sex difference in the role of cingulate RARB on neuropathic pain and affective disorders. In contrast, RARB in the ACC may not be involved in depression with no pain, since we found no significant alteration of cingulate RARB in rodent models of chronic CORT and CRS exposure. These results are consistent with changes in ACC LAMB1 in our previous study (11). In support of this assumption, previous studies have shown an increased RARA and its transcriptional regulation of corticotrophin-releasing hormone gene expression in the paraventricular nucleus in patients with affective disorders (46). A reduced level of RARA and its transcriptional target gene TrkB has been reported in the dorsolateral prefrontal cortex of elderly depressed patients (47). These data indicate that depression comorbid with neuropathic pain and depression without pain may involve different types of RARs and transcriptional regulation of different target genes in different brain regions. Additionally, RARB in the hippocampus was reported to be associated with learning and memory (19, 45).

Then, how does RARB in the ACC accomplish regulation of neuropathic pain and comorbid anxiodepression? Structural and functional plasticity in the ACC is assumed to be a cellular basis for neuropathic pain and associated anxiodepression (5–8). Emerging evidence has documented the key significance of RAR signaling in synaptic transmission and homeostatic synaptic plasticity in many brain regions, e.g., the hippocampus (19, 30, 48, 49), the somatosensory cortex (50), the visual cortex (51), the spinal cord (52), etc. Another intriguing finding of this study entails the contribution of RARB to the ACC in negatively orchestrating cingulate structural and functional plasticity. In the present study, we observed that overexpression of cingulate RARB normalized abnormal spine remodeling after SNI. These results confirm that RARB plays a pivotal role in synaptic spine stabilization in ACC pyramidal neurons. Functional synaptic plasticity is closely related to synaptic spine remodeling, both of which collectively contribute to various pathophysiological processes, including chronic pain (53). Consistent with spine remodeling, patch-clamp recordings revealed that overexpression of RARB significantly relieved SNI-induced hyperexcitability and AMPAR-mediated synaptic potentiation in ACC pyramidal neurons. Further mechanistic analysis revealed that both pre- and postsynaptic mechanisms were involved in the above-described synaptic modulation by RARB. This crucial role of RARB in cingulate functional plasticity was further strengthened by alternative in vivo evidence by using fiber photometry recording. GCaMP6s-based imaging showed that overexpression of ACC RARB inhibited photometric Ca^2+^ transients of ACC pyramidal neurons in response to peripheral stimuli and tail suspension after SNI, while knockdown of cingulate RARB sensitized Ca^2+^ responses. Overall, we inferred that exogenous supplementation of RARB normalized abnormal structural and functional plasticity of ACC pyramidal neurons after peripheral neuropathy, which in turn produced analgesic, anxiolytic, and antidepressive effects.

How RARB as a nuclear receptor is regulated upon nerve injury and further modulates neuropathic pain and related anxiodepression is not entirely understood. Strikingly, here, we revealed the involvement of activity-dependent dysregulation of RA homeostasis in the above process. Mounting evidence has shown that RA functions as an endogenous ligand for nuclear RARs to directly regulate genomic transcription (28, 54, 55). RA is a metabolic product of vitamin A. Early studies of vitamin A and RA have mainly focused on the eye, skin, and immune and reproductive systems (15, 28, 29). However, emerging studies showed that controlled RA synthesis is essential for regulating synaptic plasticity and homeostasis, and RA deficiency is closely associated with several psychiatric and developmental disorders as well as cognitive dysfunction (21, 30, 32, 56, 57). Several clinical studies have inferred that reduced vitamin A or RA level is related to a higher risk of depression in patients with ischemic stroke and solvent-induced neuropathy, while supplementation of vitamin A or RA is inversely associated with depression and sensory abnormality (39, 58, 59). However, it remains unknown whether RA homeostasis is disturbed under neuropathic pain states comorbid with affective disorders. Interestingly, using ELISA we observed a significantly lower RA level in the serum in patients with neuropathic pain and related anxiodepression compared with healthy controls. Consistently, the reduced RA level was also seen in both the serum and ACC in SNI-treated mice with pain hypersensitivity and anxiodepression. These data indicate that dysregulated RA homeostasis in ACC is closely related to depressive comorbidities associated with chronic pain.

Then, what causes dysregulated RA homeostasis after nerve injury? It is well established that RA plays an important role in homeostatic synaptic plasticity (30, 31, 60). The neuronal activity blockade, which induces homeostatic plasticity, strongly stimulates RA synthesis in neurons and rapidly enhances synaptic strength (30). In addition to homeostatic plasticity, RA signaling is also involved in multiple forms of synaptic plasticity, e.g., LTP and/or LTD (19, 61, 62). In the present study, we observed that blockade of cingulate neuronal activity with TTX and AP5 reversed the reduction of RA level in ACC after nerve injury. This suggests that the reduced RA level after nerve injury might be partly dependent on neuronal hyperactivity in the ACC. Nevertheless, we cannot exclude the possible contribution of peripheral RA depletion in ACC RA reduction after nerve injury. The relative contributions of central versus systemic RA signaling to pain and affective comorbidities remain to be further investigated. Further mechanistic analysis demonstrated that RA synthesis is Ca^2+^ dependent; that is, in synaptically active neurons, modest basal levels of postsynaptic Ca^2+^ physiologically suppress RA synthesis, whereas in synaptically inactive neurons, decreases in the resting Ca^2+^ levels induce homeostatic plasticity by stimulating synthesis of RA that then acts in a cell-autonomous manner to increase AMPAR function (63). In the present study, our observation that the intracellular Ca^2+^ level in cingulate pyramidal neurons increased significantly after nerve injury suggests that the reduced RA level observed in patients and mice with chronic pain and comorbid affective disorders might result from further suppression of RA synthesis by increased Ca^2+^ level.

Considering the dysregulated RA homeostasis after nerve injury, we thought that rectifying this dysregulation would produce beneficial effects on neuropathic pain and comorbid affective disorders. In support of this assumption, we provide 3 lines of evidence. First, direct exogenous supplementation of RA via a different route of administration, e.g., intracingulate injection and oral delivery, had a strong ability to relieve SNI-induced pain hypersensitivity and anxiodepression. Further mechanistic analysis revealed that this analgesic as well as anxiolytic and antidepressive effects are achieved by suppression of pyramidal neuronal hyperexcitability after SNI. Second, intervention of endogenous RA homeostasis improved neuropathic pain and related anxiodepression. It is well known that RA maintains homeostasis in vivo through enzyme synthesis and metabolism (28). The RA-metabolizing enzyme CYP26 eliminates RA by hydroxylation of polar metabolites (42, 43). TLZ, a specific CYP26 inhibitor, has been widely used in clinics, and its therapeutic effects have been shown in several diseases by blocking RA metabolism (27, 42, 43). Here, our results extended a new role of TLZ in treatment of neuropathic pain and related affective disorders. Systemic or intracingulate administration of TLZ showed potent efficacy in alleviating pain hypersensitivity and anxiodepression in the neuropathic state. In parallel, enhancing endogenous RA synthetase by overexpression of ALDH1A2 in the ACC exerted desirable analgesic and antidepressive effects. These lines of evidence collectively indicate that daily supplementation of RA or vitamin A may be beneficial for pain relief and mood improvement after nerve injury. Nevertheless, there is also evidence linking RA signaling and depression-like behaviors that showed that long-term use of high-dose isotretinoin in patients with acne has potential risk of depression, and the mechanism may be related to activation of HPA axis (29, 38, 64, 65). By contrast, other studies presented the opposite view that acne causes depression and that treatment leads to an improvement in depression (29, 66, 67). Overall, there is no consistent evidence to prove the relationship between isotretinoin and affective disorders in patients treated for acne.

In summary, this study shows how cingulate RA/RARB homeostasis modulates neuropathic pain and associated anxiodepression via interaction with ECM LAMB1. Taken together with our previous study in which ECM LAMB1 conveys extracellular alterations to intracellular structural and functional plasticity (11), we revealed an intracellular-extracellular-intracellular feed-forward regulatory network underlying the comorbidity of neuropathic pain and anxiodepression. Namely, nerve injury induces dysregulated RA homeostasis and subsequent downregulated RARB/LAMB1 transcriptional signaling (intracellular), which leads to ECM abnormalities in the microstructure (extracellular) and further triggers the abnormal structural and functional plasticity via intracellular signaling cascades (intracellular), ultimately resulting in pain chronicity and related affective disorders. Moreover, our results imply that RA may act as a potential indicator for the comorbidity of neuropathic pain and affective disorders and present a promising therapeutic target for treatment of this disorder.

## Methods

### Sex as a biological variable

Sex was not considered as a biological variable.

### Animals

Adult C57BL/6 mice (6–8 weeks old) were used in all experiments except for patch-clamp recording (4–5 weeks old mice) and raised under a temperature-controlled environment with a 12 h light-dark cycle. Except for the detection of RARB level after SNI injury with both sexes of mice, all the other experiments used male mice. All tests were done in a double-blind manner.

### Animal models

#### SNI surgery.

SNI is a well-established model of peripheral nerve injury, as previously described (11). See Supplemental Methods for details.

#### Chronic CORT and CRS model.

See Supplemental Methods for details.

#### Chronic inflammatory pain model.

CFA (20 μL) was unilaterally injected into the intraplantar surface of mouse hind paws, as described previously (11).

### Clinical studies

#### Human serum samples.

Seventy-two blood samples from patients and healthy volunteers were collected within 1 year. The blood samples were collected from inpatient patients with chronic pain in the rehabilitation department at Xijing Hospital. See Supplemental Methods for the clinical sample collection criteria.

#### Grouping of blood samples.

All the participants were further assessed by pain scale (NRS), depression scale (PHQ-9 and HAMD), and anxiety scale (GAD7 and HAMA) and divided into different groups according to the assessment results.

### RA level detection

The collected blood samples from humans and mice were all centrifuged at 1,000*g* for 10 min. Serums were obtained and stored at –80°C. RA level was tested using the Human Retinoic Acid ELISA kit (CSB-E16712h; CUSABIO) and the Mouse Retinoic Acid ELISA kit (CSB-EQ028019MO; CUSABIO) separately in accordance with the manufacturer’s protocol.

### Transcription factor prediction

The potential transcription factors with significant differences from *Lamb1* were further analyzed according to previous RNA-Seq data (Gene Denovo Biotechnology) (11).

### Real-time PCR, Western blotting, and immunofluorescence staining

See the Supplemental Methods for details, including Supplemental Table 1 for the list of antibodies used.

### Stereotaxic surgery

See the Supplemental Methods for details, including Supplemental Table 1 for the viruses used.

### Behavioral analyses

All mice were allocated randomly in experimental groups. Before behavioral tests, mice were allowed to acclimatize to the behavioral testing room for 1 day, and analyses were performed in a blind manner. Mechanical stimulation threshold was assessed using von Frey hairs on the plantar surface. Thermal stimulation latency was assessed by a radiant heat source applied to the plantar surface. Anxiodepressive-like behaviors were analyzed by EPM, OFT, TST, and SPT paradigms. See the Supplemental Methods for detailed procedures.

### Golgi staining

See the Supplemental Methods for details.

### Electrophysiology

Whole-cell patch-clamp recording was performed as described previously (11). The electrophysiological properties of the recorded neurons were assessed with an Axon700B amplifier (Molecular Devices Corporation) and pCLAMP10.0 software. The input-output of AMPAR-eEPSCs was recorded from layer II/III neurons, and the stimulations were delivered by a field stimulating electrode placed in layer V/VI of the ACC. The PPR of AMPAR-eEPSCs was calculated as the amplitude of the second eEPSCs divided by that of the first eEPSCs in a pair. For membrane property analysis, depolarizing current steps (500 ms in duration and 20 pA increments) were used to detect the AP under current-clamp mode. mEPSCs were recorded at a holding potential of –70 mV in the presence of AP5 (50 μM), picrotoxin (100 μM), and TTX (0.5 μM). LTP was induced by 80 paired presynaptic pulses at 2 Hz coupled with postsynaptic depolarization at +30 mV, as reported previously (68). See the Supplemental Methods for details, including Supplemental Table 1 for the reagents used.

### Calcium imaging

Fiber photometry was used to record calcium-dependent activity dynamics during behavioral tests with a multi-channel fiber photometry recording system (ThinkerTech) as described previously (11). See the Supplemental Methods for details.

### Dual-luciferase assay

The 293FT cell line (R70007; Invitrogen) was transfected with a plasmid mixture of luciferase reporter vector, transcription factor plasmid, and Renilla luciferase vector (10:9:1) using a Lipofectamine 3000 kit (L3000075; Invitrogen). At 48 hours after transfection, luciferase was measured with the Dual-Lucy Assay kit (D0010; Solarbio) in accordance with the manufacturer’s protocol. The final results were normalized to Renilla luciferase activity. See Supplemental Table 1 for the plasmids used.

### ChIP

See the Supplemental Methods for details.

### Scanning electron microscopy

Mice were anesthetized with isoflurane and transcardially perfused with ice-cold PBS. Coronal slices (1.5 mm thickness) containing the injected ACC were prepared and decellularized as previously described (69). The obtained slices were decellularized in three cycles employing de-mineralized water, sodium deoxycholate (D8331; Solarbio), and DNase I (D8071; So-larbio) diluted in 1 M NaCl solution and Triton X-100. Then, the decellularized slices were fixed with glutaraldehyde at 4°C for 24 h. The samples were rinsed in dH_2_O and gradually dehydrated in gradient with increasing concentrations of ethanol. Samples were then stacked horizontally on a wire mesh divider to keep them flat and dried using hexamethyl disilazane (283134; Sigma-Aldrich) for about 30 min. The dried samples were mounted on an aluminum scanning electron microscopy rod with conductive copper tape and sputtered coating, then imaged with a Hitachi S-4800 scanning electron microscope using InLens SE (Hitachi). at an operating voltage of 5 kV. ECM fiber diameter was analyzed using ImageJ (NIH). See Supplemental Table 1 for the list of reagents used.

### Rescue experiments

#### Intracingulate drug delivery.

The ACCs of mice were implanted with a bilateral 26-gauge stainless steel guide cannula (0.8 mm separation; RWD Life Science) according to the above coordinates. See the Supplemental Methods for details.

#### Systemic administration.

See the Supplemental Methods for details.

### Statistics

Data were analyzed in GraphPad Prism version 8.0 (GraphPad Software) and Statistical Program for Social Sciences 21.0 software (SPSS, Inc.). The normality test was performed by the Shapiro-Wilk test. Homogeneity of variance was tested using Levene’s test. Data that met these 2 conditions were analyzed using a 2-tailed unpaired or paired *t* test, 1- and 2-way ANOVA followed by Tukey’s multiple-comparison test or Dunnett’s multiple-comparison test. Datasets that were not normally distributed were analyzed with a nonparametric test (Supplemental Table 2). Data are reported as mean ± SEM. A *P* value less than 0.05 was considered statistically significant.

### Study approval

All animal procedures were reviewed and approved by the IACUC of the Fourth Military Medical University (FMMU). All clinical samples were collected according to Declaration of Helsinki principles and approved within the Medical Ethics of the First Affiliated Hospital of FMMU (Ethics Committee approval number KY20232185-F-1) and the Chinese Clinical Trial Registry (registration number ChiCTR2300076022). All participants read and signed informed consents prior to inclusion in the study. All testing was done in a double-blind manner.

### Data availability

RNA-Seq data have been deposited in the National Center for Biotechnology Information Sequence Read Archive under accession code SRP323752. Values for all data points in graphs are reported in the Supporting Data Values file. The data generated in this study are available upon request from the corresponding authors.

## Author contributions

ZZL, WNL, KXL, and ZWD performed animal preparation. ZZL designed and performed RNA-Seq analysis. ZZL, WNL, and KXL conducted Western blotting. ZZL, FW, WJH, and RGX performed brain slice patch-clamp recording. ZZL, XLS, HY, and RZ designed and conducted clinical trials. KXL, ZWD, and YC performed and analyzed the Golgi staining. WNL, ZWD, and KXL performed immunofluorescence staining. ZZL, WNL, and RZ performed scanning electron microscopy, dual-luciferase assay, and RA level detection. ZZL, WNL, KXL, TZW, and WGC conducted behavioral and pharmacological testing. ZZL, WNL, KXL, and MMW conducted stereotaxic surgery and fiber photometry. ZZL, KXL, and XXZ analyzed data. CL, SXW, ZZL, XFJ, and XLS designed studies. CL and ZZL wrote the draft manuscript. CL, SXW, and ZZL supervised the experiments and revised the manuscript. All the authors read and approved the final manuscript.

## Supplementary Material

Supplemental data

Unedited blot and gel images

Supplemental table 2

Supporting data values

## Version 1 07/01/2025

In-Press Preview

## Version 2 09/02/2025

Electronic publication

## References

[1] Bushnell MC (2013). Cognitive and emotional control of pain and its disruption in chronic pain. Nat Rev Neurosci.

[2] Barthas F (2015). The anterior cingulate cortex is a critical hub for pain-induced depression. Biol Psychiatry.

[3] Vogt BA (2005). Pain and emotion interactions in subregions of the cingulate gyrus. Nat Rev Neurosci.

[4] Meng F (2023). Neural mechanisms of social empathy in the anterior cingulate cortex. Adv Neurol.

[5] Bliss TV (2016). Synaptic plasticity in the anterior cingulate cortex in acute and chronic pain. Nat Rev Neurosci.

[6] Koga K (2015). Coexistence of two forms of LTP in ACC provides a synaptic mechanism for the interactions between anxiety and chronic pain. Neuron.

[7] Zhuo M (2016). Neural mechanisms underlying anxiety-chronic pain interactions. Trends Neurosci.

[8] Sellmeijer J (2018). Hyperactivity of anterior cingulate cortex areas 24a/24b drives chronic pain-induced anxiodepressive-like consequences. J Neurosci.

[9] Archibald J (2020). Metabolite activity in the anterior cingulate cortex during a painful stimulus using functional MRS. Sci Rep.

[10] Dityatev A (2010). The dual role of the extracellular matrix in synaptic plasticity and homeostasis. Nat Rev Neurosci.

[11] Li ZZ (2021). Extracellular matrix protein laminin β1 regulates pain sensitivity and anxiodepression-like behaviors in mice. J Clin Invest.

[12] Vasios GW (1989). A retinoic acid-responsive element is present in the 5’ flanking region of the laminin B1 gene. Proc Natl Acad Sci U S A.

[13] Hashimoto Y (1991). Retinobenzoic acids and nuclear retinoic acid receptors. Cell Struct Funct.

[14] Mic FA (2003). Retinoid activation of retinoic acid receptor but not retinoid X receptor is sufficient to rescue lethal defect in retinoic acid synthesis. Proc Natl Acad Sci U S A.

[15] Das BC (2014). Retinoic acid signaling pathways in development and diseases. Bioorg Med Chem.

[16] Goncalves MB (2019). RARβ agonist drug (C286) demonstrates efficacy in a pre-clinical neuropathic pain model restoring multiple pathways via DNA repair mechanisms. iScience.

[17] Vasios G (1991). The late retinoic acid induction of laminin B1 gene transcription involves RAR binding to the responsive element. EMBO J.

[18] Ciancia M (2022). Retinoic acid receptor beta protects striatopallidal medium spiny neurons from mitochondrial dysfunction and neurodegeneration. Prog Neurobiol.

[19] Chiang MY (1998). An essential role for retinoid receptors RARbeta and RXRgamma in long-term potentiation and depression. Neuron.

[20] Bianchi MG (2009). The ATRA-dependent overexpression of the glutamate transporter EAAC1 requires RARbeta induction. Biochim Biophys Acta.

[21] Woloszynowska-Fraser MU (2020). Vitamin A and retinoic acid in cognition and cognitive disease. Annu Rev Nutr.

[22] Kouchmeshky A, McCaffery P (2020). Use of fixatives for immunohistochemistry and their application for detection of retinoic acid synthesizing enzymes in the central nervous system. Methods Enzymol.

[23] Hu P (2020). Retinoic acid and depressive disorders: Evidence and possible neurobiological mechanisms. Neurosci Biobehav Rev.

[24] Wallace JL, Pollen AA (2021). The genetic symphony underlying evolution of the brain’s prefrontal cortex. Nature.

[25] Shibata M (2021). Regulation of prefrontal patterning and connectivity by retinoic acid. Nature.

[26] Lane MA, Bailey SJ (2005). Role of retinoid signalling in the adult brain. Prog Neurobiol.

[27] Zhu L (2022). Variants in *ALDH1A2* reveal an anti-inflammatory role for retinoic acid and a new class of disease-modifying drugs in osteoarthritis. Sci Transl Med.

[28] Ghyselinck NB, Duester G (2019). Retinoic acid signaling pathways. Development.

[29] Bremner JD (2012). Retinoic acid and affective disorders: the evidence for an association. J Clin Psychiatry.

[30] Aoto J (2008). Synaptic signaling by all-trans retinoic acid in homeostatic synaptic plasticity. Neuron.

[31] Arendt KL (2015). Retinoic acid and LTP recruit postsynaptic AMPA receptors using distinct SNARE-dependent mechanisms. Neuron.

[32] Reay WR (2020). Polygenic disruption of retinoid signalling in schizophrenia and a severe cognitive deficit subtype. Mol Psychiatry.

[33] Zhou W, Li S (2018). Decreased levels of serum retinoic acid in chinese children with autism spectrum disorder. Psychiatry Res.

[34] Tsvetkov E (2002). Fear conditioning occludes LTP-induced presynaptic enhancement of synaptic transmission in the cortical pathway to the lateral amygdala. Neuron.

[35] Schulz PE (1995). Using paired-pulse facilitation to probe the mechanisms for long-term potentiation (LTP). J Physiol Paris.

[36] Tajerian M (2018). The hippocampal extracellular matrix regulates pain and memory after injury. Mol Psychiatry.

[37] Xu X (2018). Excessive UBE3A dosage impairs retinoic acid signaling and synaptic plasticity in autism spectrum disorders. Cell Res.

[38] Al-Fartusie FS (2019). Evaluation of some trace elements and vitamins in major depressive disorder patients: a case-control study. Biol Trace Elem Res.

[39] Yang CD (2020). Association of serum retinoic acid with depression in patients with acute ischemic stroke. Aging (Albany NY).

[40] Zhang Y (2022). Associations of dietary vitamin A and beta-carotene intake with depression. A meta-analysis of observational studies. Front Nutr.

[41] de The H (1989). Differential expression and ligand regulation of the retinoic acid receptor alpha and beta genes. EMBO J.

[42] Cao DL (2024). Cytochrome P450 26A1 contributes to the maintenance of neuropathic pain. Neurosci Bull.

[43] Stevison F (2017). Inhibition of the *all-trans* retinoic acid (*at*RA) hydroxylases CYP26A1 and CYP26B1 results in dynamic, tissue-specific changes in endogenous *at*RA signaling. Drug Metab Dispos.

[44] Goncalves MB (2018). Retinoic acid synthesis by NG2 expressing cells promotes a permissive environment for axonal outgrowth. Neurobiol Dis.

[45] Nomoto M (2012). Dysfunction of the RAR/RXR signaling pathway in the forebrain impairs hippocampal memory and synaptic plasticity. Mol Brain.

[46] Chen XN (2009). The involvement of retinoic acid receptor-alpha in corticotropin-releasing hormone gene expression and affective disorders. Biol Psychiatry.

[47] Qi XR (2015). Abnormal retinoid and TrkB signaling in the prefrontal cortex in mood disorders. Cereb Cortex.

[48] Hsu YT (2019). Synaptic retinoic acid receptor signaling mediates mTOR-dependent metaplasticity that controls hippocampal learning. Proc Natl Acad Sci U S A.

[49] Sarti F (2013). Rapid suppression of inhibitory synaptic transmission by retinoic acid. J Neurosci.

[50] Park E (2018). Postnatal ablation of synaptic retinoic acid signaling impairs cortical information processing and sensory discrimination in mice. J Neurosci.

[51] Zhong LR (2018). Retinoic acid receptor RARα-dependent synaptic signaling mediates homeostatic synaptic plasticity at the inhibitory synapses of mouse visual cortex. J Neurosci.

[52] Cao B (2022). Spinal cord retinoic acid receptor signaling gates mechanical hypersensitivity in neuropathic pain. Neuron.

[53] Kuner R, Flor H (2017). Structural plasticity and reorganisation in chronic pain. Nat Rev Neurosci.

[54] Chambon P (1996). A decade of molecular biology of retinoic acid receptors. FASEB J.

[55] Cunningham TJ, Duester G (2015). Mechanisms of retinoic acid signalling and its roles in organ and limb development. Nat Rev Mol Cell Biol.

[56] Luo T (2009). Integrating retinoic acid signaling with brain function. Dev Psychol.

[57] Xu D (2023). WDR62-deficiency causes autism-like behaviors independent of microcephaly in mice. Neurosci Bull.

[58] Hu B (2022). Dietary zinc intake affects the association between dietary vitamin A and depression: a cross-sectional study. Front Nutr.

[59] Hamed EA (2016). Role of monocyte chemoattractant protein-1, stromal derived factor-1 and retinoic acid in pathophysiology of neuropathic pain in rats. J Basic Clin Physiol Pharmacol.

[60] Lenz M (2021). All-trans retinoic acid induces synaptic plasticity in human cortical neurons. Elife.

[61] Misner DL (2001). Vitamin A deprivation results in reversible loss of hippocampal long-term synaptic plasticity. Proc Natl Acad Sci U S A.

[62] Cocco S (2002). Vitamin A deficiency produces spatial learning and memory impairment in rats. Neuroscience.

[63] Wang HL (2011). Decrease in calcium concentration triggers neuronal retinoic acid synthesis during homeostatic synaptic plasticity. J Neurosci.

[64] Bremner JD, McCaffery P (2008). The neurobiology of retinoic acid in affective disorders. Prog Neuropsychopharmacol Biol Psychiatry.

[65] Hu P (2013). All-trans retinoic acid-induced hypothalamus-pituitary-adrenal hyperactivity involves glucocorticoid receptor dysregulation. Transl Psychiatry.

[66] Schrom K (2016). Depression screening using health questionnaires in patients receiving oral isotretinoin for acne vulgaris. J Am Acad Dermatol.

[67] [No authors listed] (2003). Acne, isotretinoin and depression. Drug Ther Bull.

[68] Guo B (2019). Anterior cingulate cortex dysfunction underlies social deficits in Shank3 mutant mice. Nat Neurosci.

[69] De Waele J (2015). 3D culture of murine neural stem cells on decellularized mouse brain sections. Biomaterials.

