# Supplementary material for: Cingulate retinoic acid signaling regulates neuropathic pain and comorbid anxiodepression via extracellular matrix homeostasis
Source: J Clin Invest. 2025 Jul 1;135(17):e190539. doi: 10.1172/JCI190539 (PMC12404743; doi:10.1172/JCI190539)
Supplement: Supplemental data [file jci-135-190539-s168.pdf]

Supplemental Methods

Animal models

Spared nerve injury surgery. Mice were anesthetized by isoflurane. The left sciatic nerve branches (sural, common peroneal and tibial nerves) were isolated under aseptic surgical conditions by blunt dissection of the femoral biceps muscle. The peroneal and tibial nerves were both tightly ligated and transected distal to the ligation, leaving the sural nerve intact. The muscle and skin were then sutured and sterilized with entoiodine after surgery. Sham mice were subjected to all preceding procedures without nerve ligation and transection.

Chronic CORT. Mice were exposed to 0.1 mg/ml Corticosterone (CORT) (Dissolved by ethanol to 10 mg/ml and added in animal drinking water) for 21 d. A control group was exposed to regular drinking water with 1% ethanol.

Chronic restraint stress model. Mice were restrained in tubes full of holes for ~4 h per day for 21 d. Except for these 4 hours, mice had regular activity and diet for the remainder of the day. A control group had gentle handling for ~15 min per day for 21 d.

Chronic inflammatory pain model. Unilateral injection of complete Freund's adjuvant (CFA) (20 μl) was performed into the intraplantar surface of mouse hindpaw, as described previously. Western blotting was performed to examine the changes of RARB in the ACC at 14 d following mice suffered with hyperalgesia and anxiodepression induced by CFA inflammation.

Clinical samples collection criteria

The patients need to meet inclusion criteria as below: (1) Age: Over 18 years old (the boundary value), gender is not limited; (2) Patients with spinal cord injury or peripheral pain (soft tissue injury or bone and joint injury causing pain); (3) Patients with NRS score > 3 and pain lasting for more than 1 month. While, patients with any of the following conditions are not eligible for clinical trials: (1) Women whose pregnancy tests (only women of childbearing age) are positive; (2) Patients with severe cardiovascular, lung, immune system, skin, skeletal muscle, and nervous system diseases that the researchers considered unsuitable for inclusion; (3) Patients addicted to alcohol or drugs (long-term use and dependence on alcohol or drugs); (4) Patients with neurological diseases (stroke, epilepsy) and family history of anxiety and depression; (5) Patients with vitamin A allergy; (6) Patients who have used acne cream (containing isotretinoin) and vitamins (containing vitamin A) in the past three months; (7) Participants who are considered inappropriate to participate in this clinical trial for other reasons.

Behavioral analyses

Mechanical allodynia. All mice were placed in the individual test compartments on an elevated mesh-bottomed platform at least 1 h before test. Mechanical withdrawal threshold test was performed using von Frey filaments (0.008, 0.02,

0.04, 0.07, 0.16, 0.4, 0.6, 1.0, 1.4 and 2.0 g). From 0.008 g to 2.0 g filament, filaments were touched to the lateral plantar skin with just enough force to blend the fiber and hold for 1 s. A positive response to the mechanical stimuli was defined as foot lift under von Frey filament treatment. Each filament was applied 5 times and the paw response was recorded. The mechanical threshold was performed by nonlinear fitting followed by cumulative Gaussian test.

Thermal nociception. This experiment was performed using a device (IITC model 400, Woodland Hills, CA, USA) to identify thermal sensitivity of mice. Mice were placed in the individual test compartments on a temperature-controlled glass platform kept to 30°C, and the lateral plantar skin of hindpaw were stimulated with a radiant heat source (50 W halogen bulb) directed through an aperture. The duration to the stimulus until paw withdrawal was recorded as paw withdrawal thermal latency. Each hindpaw was tested 5 times with 5-min intervals, and the withdrawal latency values averaged. To avoid tissue damage by prolonged thermal stimuli, cut off latency was set as 30 s.

Open field test. All tests were performed during the dark phase, and the light intensity was controlled in the same condition. The open field chamber was a 50 × 50 × 40 cm^3 box, and the mice were placed into the central area at the start of the test. The activity of the mouse was video-recorded 10 min. Between sessions, the chamber was thoroughly cleaned with 70% ethanol to remove residual odors. The parameters measured included the time and distance traveled in the central area and the total distance moved. The zone crossings

were analyzed with SMART v.3.0 software (Panlab Harvard Apparatus, Newbury Park, CA, USA).

Elevated plus-maze test. All tests were performed during the dark phase, and the light intensity was controlled in the same condition. This test was performed in a maze consisting of two open arms ($30 \times 5\ cm^2$) and two enclosed arms ($30 \times 5 \times 30\ cm^3$) that extended from a central platform ($5 \times 5\ cm^2$) at 90°. The mice were placed into the central platform facing an open arm at the start of the test. The activity of the mouse was video-recorded 5 min. Between animals, the maze was thoroughly cleaned with 70% ethanol to remove residual odors. The parameters measured included the time and distance traveled in the open arm area and the total distance moved. The zone crossings were analyzed using SMART v.3.0 software.

Tail suspension test. Mice were suspended about 15 cm from the top of a compartment using lab tape and video-recorded for 6 min. Meanwhile, a small plastic tube was placed around the tail to avoid tail climbing. The behavioral apparatus was thoroughly cleaned with 70% ethanol to remove residual odors between animals. Immobility time was calculated with consistent criteria by observer blind to the experimental condition of the animals.

Sucrose preference test. Before the test, each mouse was temporarily single-housed in a cage, and trained to adapt to 1% sucrose solution (W/V): two bottles of 1% sucrose solution were placed in each cage. After 24 h, one sucrose bottle was replaced with a bottle of water for 24 h. Then mice were deprived of water

and food for 24 h. After that, a bottle of 1% sucrose solution and a bottle of water were placed in each cage for 12 h. Sucrose solution and water consumption were calculated by weighing the bottle before and after 12 h testing session, and the sucrose reference was calculated as follows: sucrose preference (%) = sucrose consumption/ (sucrose consumption + water consumption).

Real-time PCR

Total RNA was extracted from cells or tissues using TRIzol Reagent (#15596018, Invitrogen, Carlsbad, CA, USA) in accordance with the manufacturer's protocol. RNA extracts were reverse-transcribed by PrimeScript RT Master Mix (#RR036A, TaKaRa, Dalian, China) at 37 °C for 15 min and at 85 °C for 5 s. Targets were then amplified in triplicate by quantitative RT-PCR using TB Premix Ex Taq (#RR820A, TaKaRa) on a StepOnePlus Real-Time PCR System (Applied Biosystems, Foster City, CA, USA), normalized to GAPDH, and quantified by the comparative cycle threshold method ($2^{-\Delta\Delta CT}$). Primer sequences as followed. RARB: Forward sequences, CAATGCTGGCTTCGGTCCTCTG; Reverse sequences, CCTCAAGGTCCTGGCGGTCTC. GAPDH: Forward sequences, TGTGTCCGTCGTGGATCTGA; Reverse sequences, TTGCTGTTGAAGTCGCAGGAG.

Western blot

ACC were dissected from brain of mice and lysed using RIPA lysis buffer by sonication on ice, and centrifuged at 12,000 rpm for 10 min. Total protein was measured by BCA Protein Assay Kit (#23225, Thermo Scientific, Waltham, MA, USA), mixed with 5× SDS-PAGE loading buffer (#CW0027, CWBIO, Beijing, China), and heated at 100 °C for 10 min. Protein samples were resolved by SDS-PAGE, and immunoblotted with corresponding antibodies. Proteins were visualized with enhanced chemiluminescence detection methods. The scanned images were quantified using ImageJ software. Specific bands for each protein were normalized to its respective GAPDH loading control.

Immunofluorescent staining

Mice were anesthetized by isoflurane, and perfused intracardially with ice cold 0.9% salt saline and 4% paraformaldehyde phosphate buffer. Brain tissues were removed and dehydrated by 30% sucrose. Serial sections (16 μm in thickness) were cut. The sections were dried at room temperature for 4-6 h, and blocked with PBS containing 0.1% triton X-100 and 5% bovine serum albumin (BSA) for 1 h. After that, corresponding primary antibodies were incubated overnight at 4 °C. After wash 3 times, corresponding secondary antibodies for 2-4 h in the dark at room temperature. The nuclei were stained by DAPI. All immunostained sections were captured under a confocal microscope (FV3000, Olympus, Ishikawa, Japan) with the same setting.

Stereotaxic surgery

Mice were anesthetized with isoflurane, and their heads were fixed in a stereotactic frame (RWD Life Science Inc., Shenzhen, China). Their eyes were applied to erythromycin to prevent corneal drying. All skull calibrations were made relative to Bregma, and virus was injected into the ACC at a rate of 30 nl/min using a 10 μl microsyringe (Gaoge, Shanghai, China) with a microelectrode to deliver the virus using a microsyringe pump (Kd Scientific, Holliston, MA, USA). After viral injection, the microelectrode was kept in place for 10 min to allow diffusion of the virus. The stereotaxic coordinates for ACC injection were anterior posterior (AP) 1.00, medial lateral (ML) 0.25 and dorsal ventral (DV) 1.80 mm. For the conditional interfering gene expression experiments, 300 nl of AAV virus was unilaterally injected into the right ACC. For calcium imaging experiments, 300 nl mixture of a 1:2 ratio of AAV2/9-CaMKIIa-GCaMP6s-EGFP and rAAV2/9-CaMKIIα-Rarb-3×FLAG-P2A-mCherry-WPRE-hGH polyA or rAAV2/9-CaMKIIα-mCherry-5'miR-30a-shRNA(Rarb)-3'miR-30a-WPREs was injected unilaterally into the right ACC, and fiber optical cannula (250 μm optical density (OD), 0.37 numerical aperture (NA), Thorlabs) were implanted in the contralateral ACC (from Bregma: AP +1.00, ML 0.25, DV 1.6). Data were excluded in experiments when the viral injections were inaccurate.

Golgi staining

Animals were perfused with 0.9% saline solution. The brain tissue was isolated and immersed in Golgi-cox solution (5% potassium chromate, 5% potassium dichromate, and 5% mercuric chloride) for fixation, and maintained in the dark at room temperature for 2-3 d. Then, the brains were transferred to a fresh Golgi-Cox Solution for 14 d. After that, the brains were transferred to 30% sucrose for 2 d. Coronal sections (150 mm) were dissected serially. For staining, brain sections were washed in deionized water for 1 min, placed in 50% NH_4OH for 30 min, and subsequently in fixing solution (Kodak; Rochester, NY, USA) for an additional 30 min. The sections were then incubated in 5% sodium thiosulfate for 10 min. After washing with distilled water, dehydrated with gradient ethanol. The sections were finally mounted and captured using an FV1200 confocal microscopy (Olympus, Ishikawa, Japan) and Imaris software (v.7.7.1, Bitplane, Switzerland) was used to reconstruct neuronal dendritic arbors and dendritic spines.

Calcium imaging

An optic fiber cannula was implanted into the ACC of mouse as described above. To excite GCaMP6s, a 473-nm LED (Cree XP-E LED, Shenzhen, China) was reflected off a dichroic mirror (MD498, Thorlabs, Newtown, NJ, USA) that was focused by a ×20 objective lens (0.4 NA; Olympus, Ishikawa, Japan) and coupled to an optical commutator (Doris Lenses). An optical fiber (230 μm OD,

0.37 NA) guided the light between the commutator and the implanted optical fiber. The laser power at the tip of the optical fiber was adjusted to 0.01-0.02 mW to decrease laser bleaching. Fluorescence was bandpass-filtered (MF525-39, Thorlabs), and an amplifier was used to convert the CMOS (DCC3240M, Thorlabs) current output to a voltage signal. The analog voltage signals were digitalized at 50 Hz and recorded by the multi-channel fiber photometry recording system (ThinkerTech). Data analyses were used MATLAB software (Mathworks). Fluorescence signals were recorded continuously during behavioral test and normalized (ΔF/F) by calculating the median signal across the recording period, subtracting this from each data point, and dividing by the median signal.

Electrophysiology

Whole-cell patch-clamp recording was performed as described previously. Briefly, viral injection of AAV2/9-RARB was performed into unilateral ACC. After three weeks, mice were anesthetized with isoflurane and transcardially perfused with ice-cold oxygenated (95% O_2, 5% CO_2) incubation solution (in mM: NaCl, 95; KCl, 1.8; KH_2PO_4, 1.2; $CaCl_2$, 0.5; $MgSO_4$, 7; $NaHCO_3$, 26; glucose, 15; sucrose, 50; pH 7.4). The brain was removed and fixed on a vibratome (Leica1200s, Solms, Germany), coronal slices (300 μm-thick) containing the injected ACC (bregma 1.42 to 0.50 mm, determined by the shapes of lateral ventricles and corpus callosum) were prepared using the

vibratome and incubated at 32 ± 1°C for at least 1 h. A slice was then transferred into a recording chamber and superfused with oxygenated recording solution at 2 ml / min. The recording solution was identical to the incubation solution except for (in mM): NaCl 127, $CaCl_2$ 2.4, $MgSO_4$ 1.3 and sucrose 0. Standard whole-cell patch clamp recordings were performed with glass pipettes having a resistance of 3-5 MΩ in fluorescence-labelled pyramidal neurons in layer II/III of ACC. Recorded pyramidal neurons were identified based on their morphological properties and their ability to show spike frequency adaptation in response to the prolonged depolarizing-current injection. The pipette solution consisted of (in mM): K-gluconate, 135; KCl, 5; $CaCl_2$, 0.5; $MgCl_2$, 2; EGTA, 5; HEPES, 5 and Mg-ATP, 5, pH 7.4 with KOH, measured osmolarity 300 mOsm. The electrophysiological properties of the recorded neurons were acquired with an Axon700B amplifier (Molecular Devices Corporation, CA, USA) and pCLAMP10.0 software. Except for AP recording, QX-314 (5 mM) was added to the pipette solution to prevent discharge of AP. Data were excluded when the resting membrane potential of neurons were positive than −55 mV and AP did not have overshoot. Signals were low-pass filtered at 5 kHz, sampled at 10 kHz and analysed offline.

Input-output of AMPAR-mediated eEPSCs. The cell membrane potential was held at -70 mV. AMPAR-mediated evoked EPSCs (eEPSCs) were recorded from layer II/III neurons, and the stimulations were delivered by a field stimulating electrode placed in layer V/VI of the ACC. AMPAR-mediated

eEPSCs were induced by repetitive stimulations at 0.05 Hz with increasing intensity in the presence of AP5 (50 μM), an antagonist at NMDA receptors and picrotoxin (100 μM), blocker for inhibitory synaptic transmission.

Paired-pulse ratio (PPR) analysis. In a subset of experiments, paired-pulse stimuli with an inter-stimulus interval of 50 ms (0.1 ms pulse duration, 1 mA intensity, every 30 s) were applied to layer V/VI of the ACC. Paired-pulse ratio (facilitation or depression) of AMPARs-mediated eEPSCs was calculated as the amplitude of the second eEPSCs divided by that of the first eEPSCs in a pair.

Intrinsic membrane properties. For membrane properties analysis, depolarizing current steps (500 ms in duration and 20 pA increments) were used to detect the AP under current-clamp mode. The AP threshold was determined by differentiating the AP waveform and setting a rising rate of 10 mV/ms as the AP inflection point. The AP amplitude was measured from the equipotential point of the threshold to the spike peak. The AP duration was measured at the half-width of the spike.

Miniature EPSCs (mEPSCs) analysis. mEPSCs were recorded at a holding potential of −70 mV in the presence of AP5 (50 μM), picrotoxin (100 μM) and tetrodotoxin (TTX) (0.5 μM). mEPSCs were analyzed using Mini Analysis (Synaptosoft Inc.).

Chromatin immunoprecipitation (ChIP)

Chromatin immunoprecipitation (ChIP) assays were performed by a

SimpleChIP® Plus Enzymatic Chromatin IP Kit (9005 S, Cell Signaling Technology, Boston, MA, USA). In brief, chromatin from 25 mg ACC were chemically cross-linked with 1.5% formaldehyde solution for 20 min at room temperature and quenched with glycine. The tissue was homogenized to generate a single-cell suspension, and lysised DNA with micrococcal nuclease at 37 °C for 20 min, which was followed by an appropriate amount of sonication. The supernatants were immunoprecipitated with 2 μg of Anti-RARB antibody, Normal Rabbit IgG as negative control or 10 ul Histone H3 Rabbit mAb as positive control at 4 °C for 12-16 h. After that, the immunocomplexes were incubated with 30 μl ChIP-Grade Protein G Magnetic Beads at 4 °C for 2 h. Chromatin was eluted by ChIP elution buffer at 65 °C for 30 min, and reversed cross-links with 5 M NaCl and proteinase K at 65 °C for 2 h. ChIP DNA was purified and subsequently quantified by PCR and quantitative real-time PCR. Data analysis was finally presented as percentages of the input DNA. The primer sequences from Lamb1 promotor were used as followed: forward: GGCTGGAGAGATGGCATTGT; Reverse: ATGGTTGTGAGCCACCATGT.

Rescue experiments

Intracingulate drug delivery. The ACC of mice were implanted with bilateral 26 gauge (Ga) stainless steel guide cannula (0.8 mm separation, RWD Life Science) according to the above coordinates. The removable obturators (33 Ga OD) at the full length of guide cannula were inserted into guide cannula to limit

obstruction. After surgery, animals were housed separately and recovered for ~7 d. Mice were lightly anesthetized using isoflurane and obturators were removed. Sterile 33 Ga microinjection needles were connected to two 10 μl microsyringes (Hamilton, Bonaduz, Switzerland) via PE-50 tube. 500 nl Retinoic acid, Talarozole, or vehicle was infused per side over a 2 min period. Then, the microinjection needles were kept in place for additional 10 min for drug diffusion. After the diffusion, the injection needle was removed and the obturator was replaced. Behavioral tests were performed after microinjection ~1 h.

Systemic administration. Sham or SNI-operated mice were treated with retinoic acid (0.6 mg/kg) or vehicle via oral administration for 2 consecutive weeks, behavioral tests were then performed. On the other hand, mice were administered with Talarozole (0.1, 1 mg/Kg) via intraperitoneal injection, and behavioral tests were performed 1 h later.

Supplemental figure legends:

Supplemental Figure 1: RARB overexpression in ACC relieves pain hypersensitivity and anxiodepression induced by SNI model. (A-D) Bilateral mechanical sensitivity and thermal sensitivity were unaltered by RARB overexpression in Sham-treated state (n = 10). 2-tailed unpaired *t* test. (E) Quantitative summary showing distance and time staying in the open arm and total distance in Sham- and SNI-treated mice (n = 9-10). 1-way ANOVA. Data are presented as mean ± S.E.M. $^*P < 0.05$, $^{**}P < 0.01$, $^{***}P < 0.001$. PWMT, paw withdrawal mechanical threshold; PWTL, paw withdrawal thermal latency.

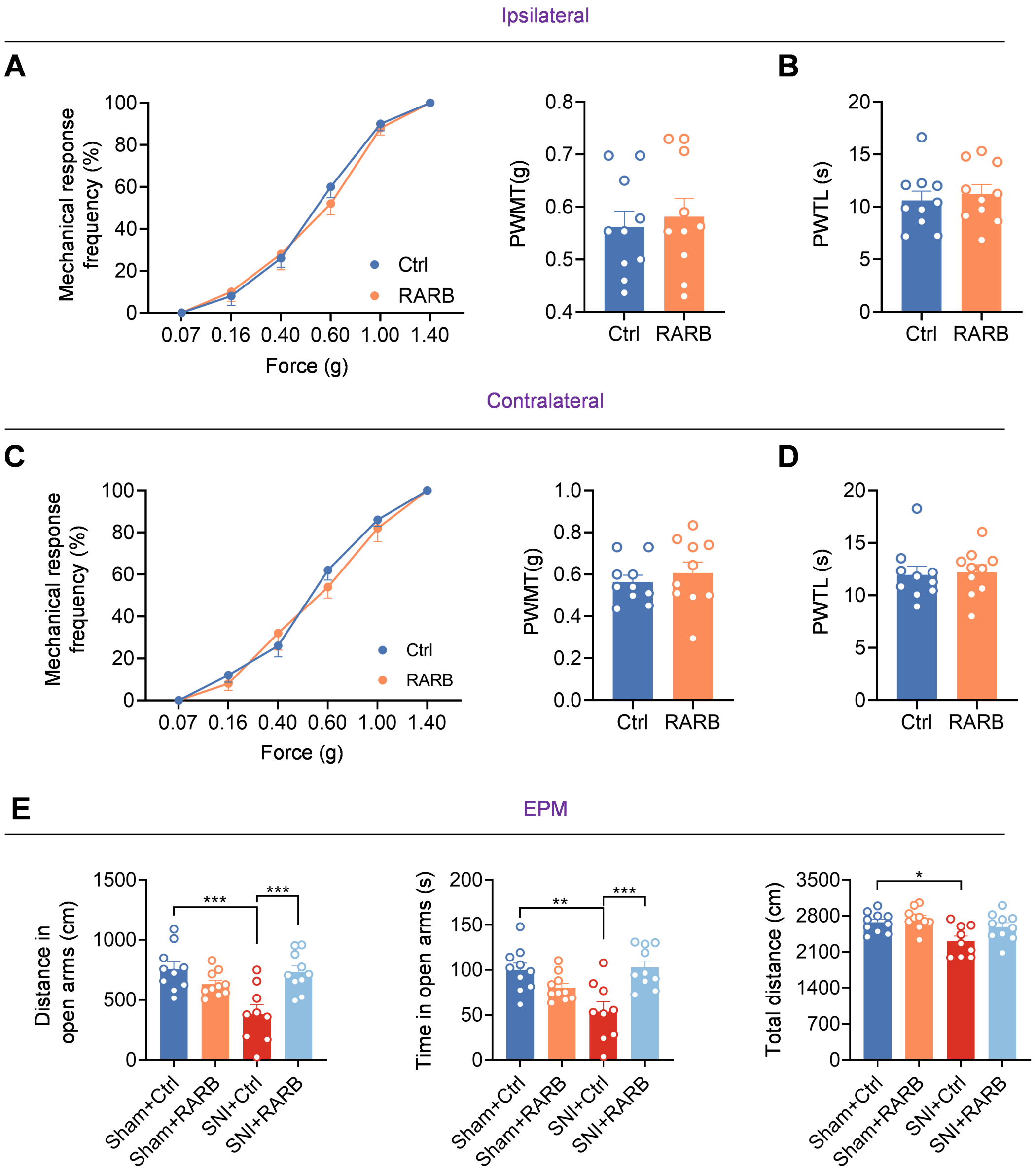
Ipsilateral
A
Mechanical response frequency (%)
Force (g)
0.07 0.16 0.40 0.60 1.00 1.40
Ctrl
RARB
PWMT(g)
B
PWTL (s)
Contralateral
C
D
E
EPM
Distance in open arms (cm)
Time in open arms (s)
Total distance (cm)
Sham+Ctrl
Sham+RARB
SNI+Ctrl
SNI+RARB
***
**
*

Supplemental Figure 2: (A) Representative images of pyramidal neurons in the ACC derived from Sham- and SNI-treated mice overexpressing RARB (n = 18-23). Scale bars: 100 μm. (B and C) Sholl analysis of dendritic branching complexity in the basal and apical dendrites of Sham- or SNI-operated mice overexpressing RARB (n = 18-23). 1-way ANOVA. (D) Summary of spine length of apical dendrites of ACC pyramidal neurons obtained from mice overexpressing RARB in both Sham and SNI conditions (n = 17-20). Kruskal-Wallis *H* test. (E) Summary of the density of long thin- and filopodia- shaped spines on apical dendrites of ACC pyramidal neurons from mice of the above 4 groups (n = 17-20). Kruskal-Wallis *H* test. (F) Representative confocal stacks and three-dimensional reconstruction images of the basal dendrites of ACC pyramidal neurons obtained from mice overexpressing RARB in both Sham and SNI conditions (n = 20-21). Scale bar: 5 μm. (G) Summary of spine length and density on the basal dendrites of ACC pyramidal neurons obtained from mice overexpressing RARB in both Sham and SNI conditions (n = 20-21). Length: 1-way. Density: Kruskal-Wallis *H* test. (H) Summary of the density of stubby-, mushroom-, long thin- and filopodia-shaped spines on the basal dendrites of ACC pyramidal neurons in the above four groups (n = 20-21). Kruskal-Wallis *H* test for density of stubby-, mushroom- and long thin-shaped spines. 1-way ANOVA for filopodia-shaped spines. Data are presented as mean ± S.E.M. *$P < 0.05$, **$P < 0.01$, ***$P < 0.001$, ****$P < 0.0001$.

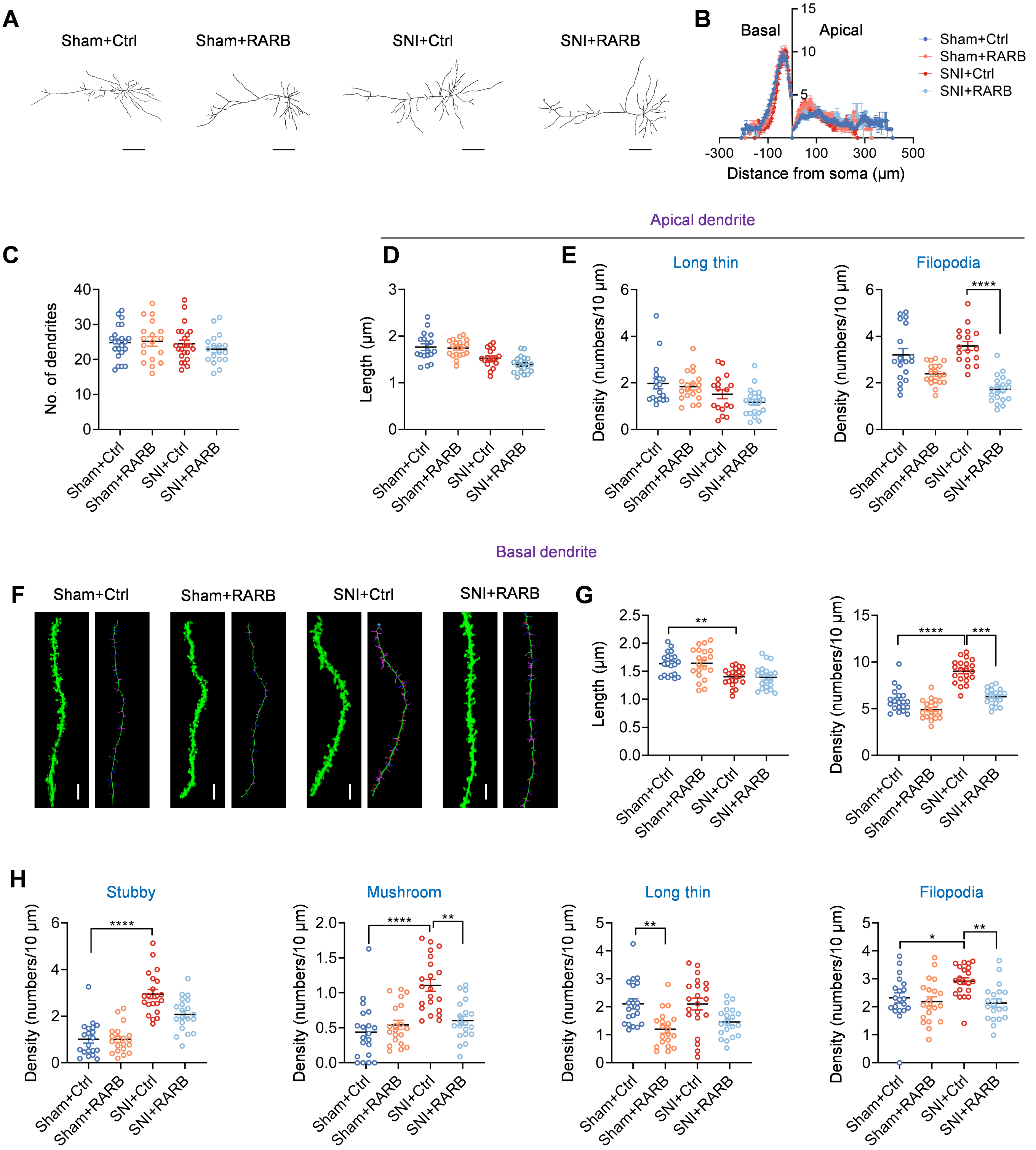
A
Sham+Ctrl
Sham+RARB
SNI+Ctrl
SNI+RARB
B
Basal
Apical
Sham+Ctrl
Sham+RARB
SNI+Ctrl
SNI+RARB
Distance from soma (μm)
Apical dendrite
C
No. of dendrites
D
Length (μm)
E
Long thin
Filopodia
Density (numbers/10 μm)
Basal dendrite
F
G
H
Stubby
Mushroom
Long thin
Filopodia
Density (numbers/10 μm)

Supplemental Figure 3: (A) Detailed analysis of resting membrane potential (RMP), membrane resistance (Rm) and membrane capacitance (Cm) between mice expressing RARB and mCherry alone in both Sham- and SNI-injured state (n=8-11). RMP and Rm: Kruskal-Wallis *H* test. Cm: 1-way ANOVA. (B) Detailed analysis of action potential (AP) properties (n = 8-11), such as amplitude, threshold, half-width and afterhyperpolarization (AHP) in both Sham- and SNI-treated mice overexpressing RARB. AP amplitude and AHP: Kruskal-Wallis *H* test. AP threshold and half-width: 1-way ANOVA. Data are presented as mean ± S.E.M.

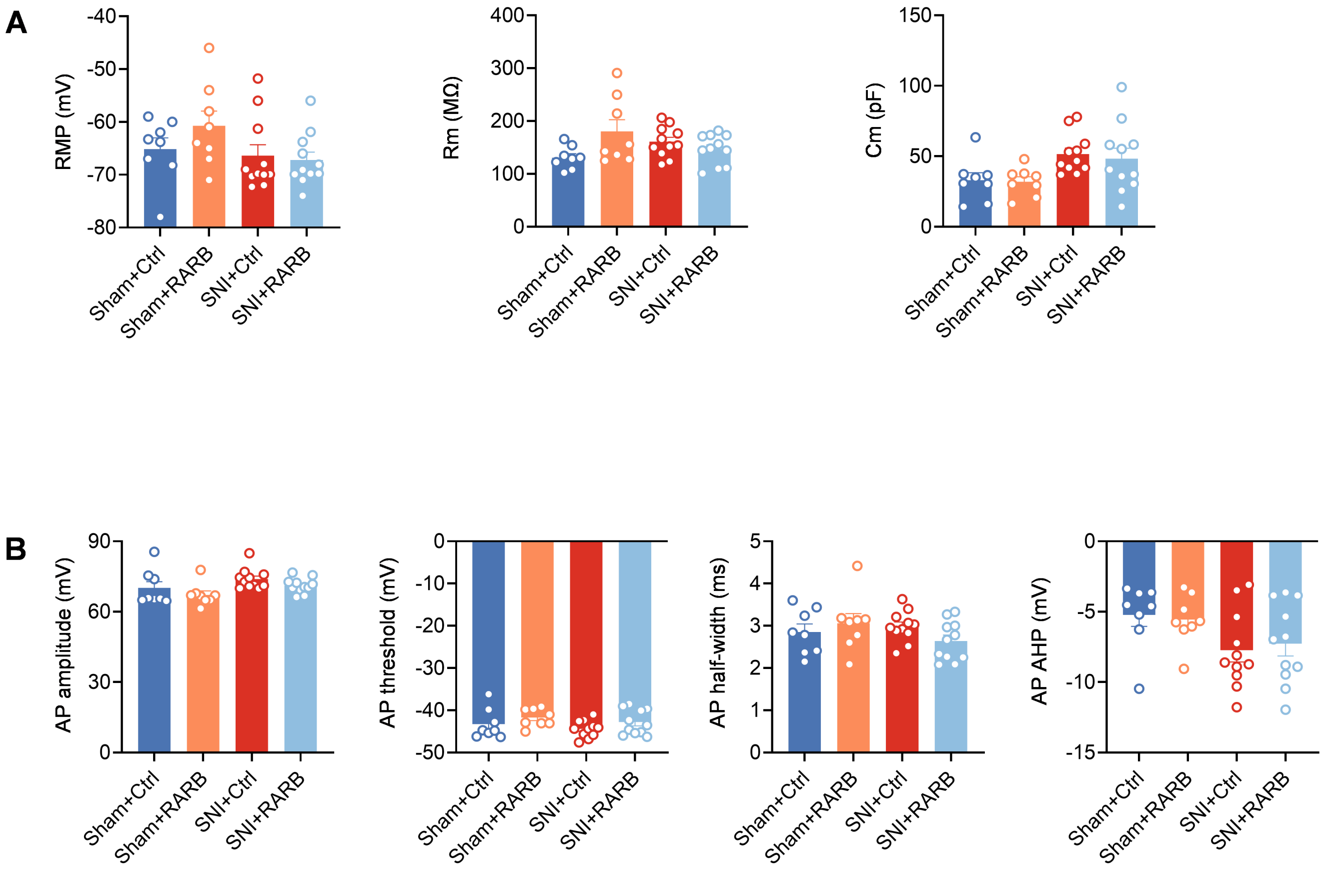
A
RMP (mV)
Rm (MΩ)
Cm (pF)
B
AP amplitude (mV)
AP threshold (mV)
AP half-width (ms)
AP AHP (mV)
Sham+Ctrl
Sham+RARB
SNI+Ctrl
SNI+RARB

Supplemental Figure 4: RARB knockdown in ACC induces pain hypersensitivity and anxiodepression. (A) Stimulus-response curve and mechanical threshold showing ACC RARB knockdown exacerbates contralateral mechanical sensitivity in Sham-treated mice (n = 12-16). Mann-Whitney *U* test. (B) Contralateral thermal sensitivity was aggravated by RARB knockdown in Sham-treated state (n = 12-16). 2-tailed unpaired *t* test. (C) Quantitative summary showing time staying in the open arm and total distance in Sham-treated mice expressing shRarb (n = 11-14). 2-tailed unpaired *t* test. (D) Quantitative summary showing time staying in the centre area and total distance in Sham-treated mice expressing shRarb (n = 11-13). 2-tailed unpaired *t* test. (E) Quantitative summary of AUC in 5 independent experiments of peak GCaMP6s signals from the Figure 5 K-N. Mann-Whitney *U* test for 0.4g and 2 g mechanical stimuli record, and struggling record. 2-tailed unpaired *t* test for thermal nociception. (F-G) Representative photometry traces as shown in heat maps and quantitative summary from 5 independent experiments of peak GCaMP6s signals locked to the brush stimuli (F) and pinprick nociception (G). $\Delta F / F$: 2-tailed unpaired *t* test. AUC: Mann-Whitney *U* test. Data are presented as mean ± S.E.M. *$P < 0.05$, **$P < 0.01$, ***$P < 0.001$, ****$P < 0.0001$.

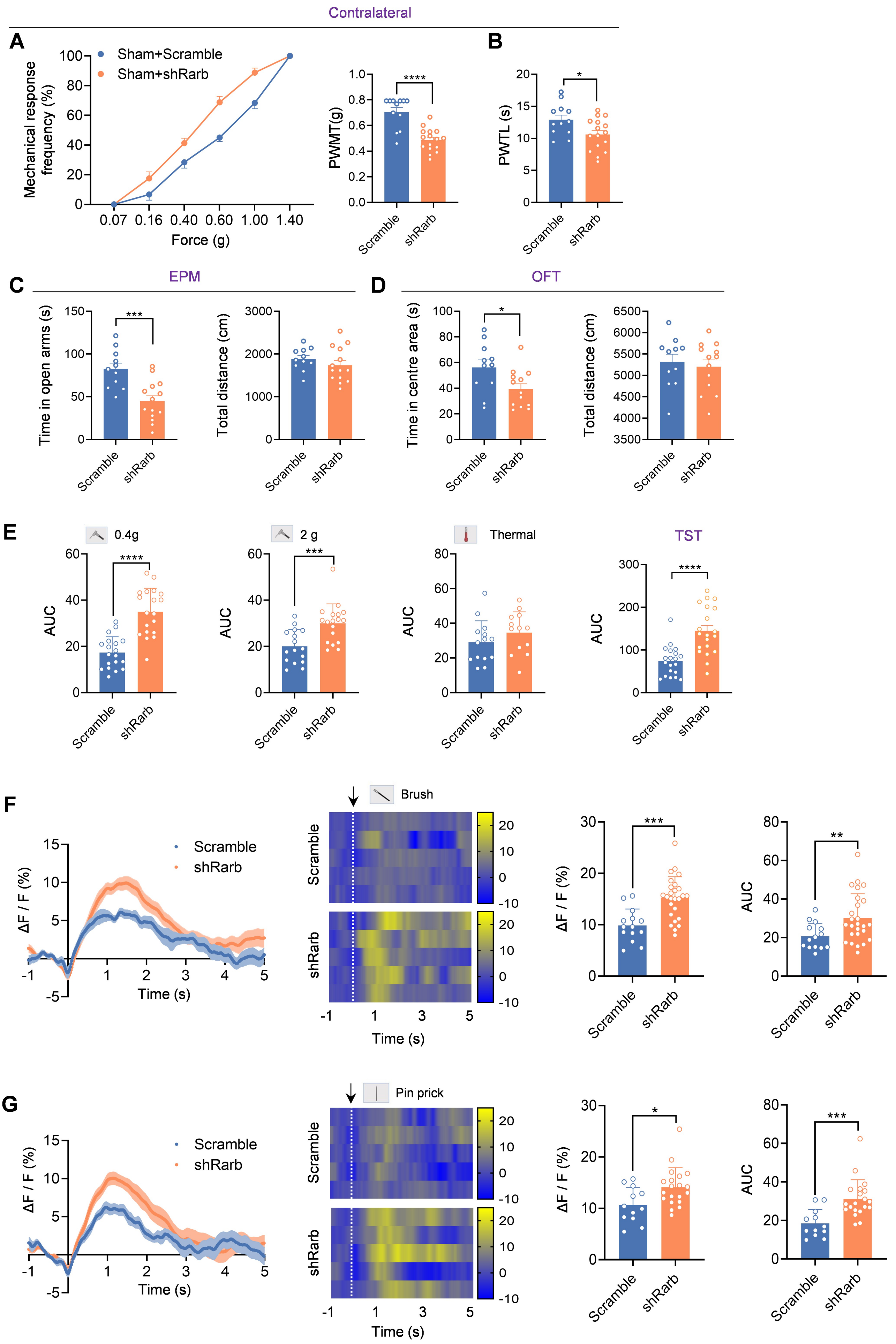
Contralateral
A
Sham+Scramble
Sham+shRarb
Mechanical response frequency (%)
Force (g)
0.07 0.16 0.40 0.60 1.00 1.40
PWMT(g)
****
Scramble
shRarb
B
PWTL (s)
*
C
EPM
Time in open arms (s)
***
Total distance (cm)
D
OFT
Time in centre area (s)
*
Total distance (cm)
E
0.4g
2 g
Thermal
TST
AUC
****
***
****
F
Brush
ΔF / F (%)
Scramble
shRarb
Time (s)
***
**
AUC
G
Pin prick
ΔF / F (%)
Time (s)
*
***
AUC

Supplemental Figure 5: (A and B) Stimulus-response curve and mechanical threshold showing ACC LAMB1 knockdown exacerbates ipsilateral mechanical sensitivity (A) and thermal sensitivity (B) in SNI-treated mice (n = 19). Mann-Whitney *U* test (A) and 2-tailed unpaired *t* test (B). (C) Quantitative summary showing distance and time staying in the centre area and total distance in SNI-treated mice expressing shLamb1 (n = 16-18). 2-tailed unpaired *t* test for Distance in centre area and Total distance. Mann-Whitney *U* test for Time in centre area. (D) Quantitative summary showing distance and time staying in the open arms and total distance in SNI-treated mice expressing shLamb1 (n = 16-18). Mann-Whitney *U* test (Distance in open arms) and 2-tailed unpaired *t* test (Time in open arms and Total distance). (E) TST showing that expression of AAV-shLamb1 resulted in longer immobility in SNI-treated mice (n = 16-18). Mann-Whitney *U* test. (F) Quantitative summary of time staying in the centre area and total distance in the OFT paradigm in SNI-treated mice expressing shLamb1 and/or RARB (n = 8-10). 1-way ANOVA. (G) Quantitative summary of time staying in the open arm and total distance in the EPM paradigm in SNI-treated mice expressing shLamb1 and/or RARB (n = 8-10). 1-way ANOVA. Data are presented as mean ± S.E.M. *P < 0.05, **P < 0.01, ****P < 0.0001.

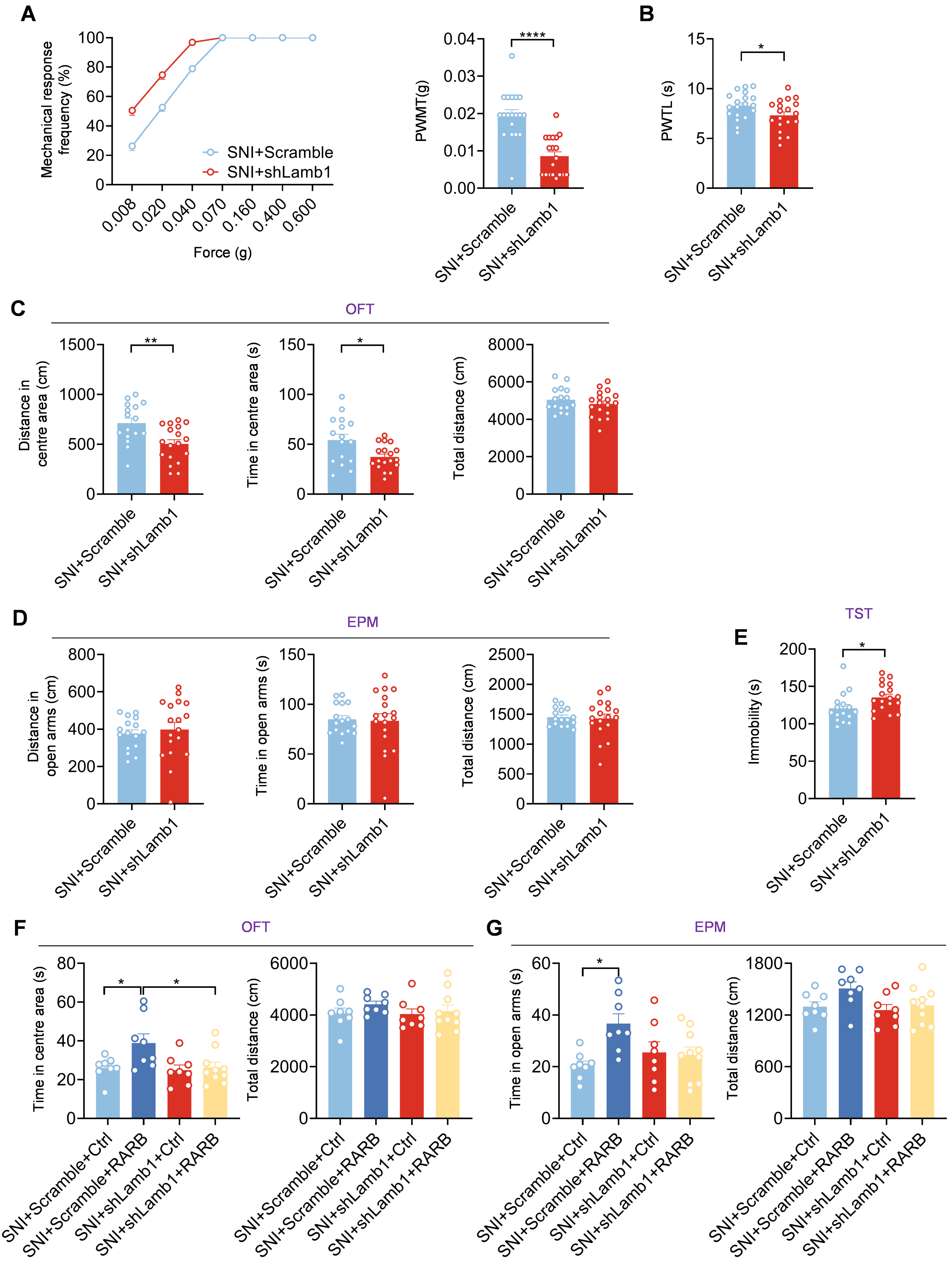

A
Mechanical response frequency (%)
SNI+Scramble
SNI+shLamb1
Force (g)
0.008
0.020
0.040
0.070
0.160
0.400
0.600
PWMT(g)
****
B
PWTL (s)
*
C
OFT
Distance in centre area (cm)
**
Time in centre area (s)
*
Total distance (cm)
D
EPM
Distance in open arms (cm)
Time in open arms (s)
Total distance (cm)
E
TST
Immobility (s)
*
F
OFT
Time in centre area (s)
Total distance (cm)
SNI+Scramble+Ctrl
SNI+Scramble+RARB
SNI+shLamb1+Ctrl
SNI+shLamb1+RARB
G
EPM
Time in open arms (s)
Total distance (cm)

Supplemental Figure 6: (A) Schematic diagram of Intra-ACC injection of RA in mice. (B, C) Contralateral stimulus-response curves, mechanical threshold (B) and thermal latency (C) showing intra-ACC injection of RA in SNI-treated mice (n = 10). PWMT: Kruskal-Wallis *H* test. PWTL: 1-way ANOVA. (D) Time staying in the open arm and total distance in SNI-treated mice after ACC delivery of RA (n = 7-10). Time in open arms: Kruskal-Wallis *H* test. Total distance: 1-way ANOVA. (E) Time staying in the centre area and total distance in SNI-treated mice after ACC delivery of RA (n = 7-10). Time in centre area: Kruskal-Wallis *H* test. Total distance: 1-way ANOVA. (F, G) Ipsilateral stimulus-response curve, mechanical threshold (F) and thermal latency (G) as shown after intra-ACC injection of RA (50 pmol) in Sham-treated mice (n = 7). 2-tailed unpaired *t* test. (H, I) Contralateral stimulus-response curve, mechanical threshold (H) and thermal latency (I) as shown after intra-ACC injection of RA (50 pmol) in Sham-treated mice (n = 7). 2-tailed unpaired *t* test. (J) EPM in Sham-treated mice after intra-ACC injection of RA (50 pmol) (n = 6-7). 2-tailed unpaired *t* test. (K) OFT after intra-ACC injection of RA (50 pmol) in Sham-treated mice (n = 7). Distance in centre area: 2-tailed unpaired *t* test. Time in centre area and total distance: Mann-Whitney *U* test. (L, M) Sucrose preference in SPT (L) and immobility in TST (M) as shown after Intra-ACC injection of RA (50 pmol) in Sham-operated mice (n = 7). 2-tailed unpaired *t* test. Data are presented as mean ± S.E.M. *$P < 0.05$.

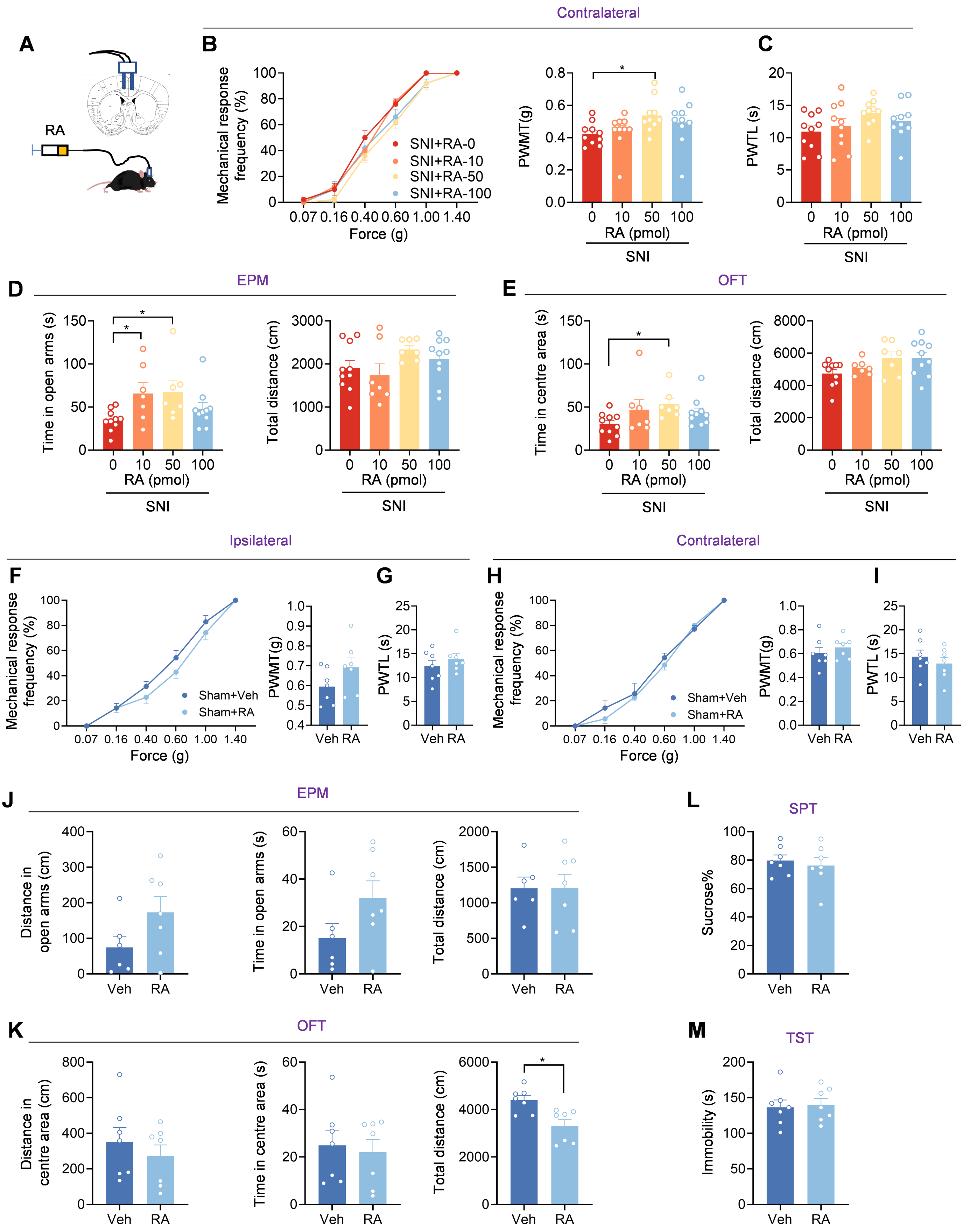

Contralateral
A
RA
B
Mechanical response frequency (%)
Force (g)
SNI+RA-0
SNI+RA-10
SNI+RA-50
SNI+RA-100
PWMT(g)
RA (pmol)
SNI
C
PWTL (s)
D
EPM
Time in open arms (s)
Total distance (cm)
E
OFT
Time in centre area (s)
Total distance (cm)
Ipsilateral
F
Sham+Veh
Sham+RA
G
PWMT(g)
PWTL (s)
Veh RA
H
I
J
Distance in open arms (cm)
Time in open arms (s)
Total distance (cm)
L
SPT
Sucrose%
K
Distance in centre area (cm)
Time in centre area (s)
M
TST
Immobility (s)

Supplemental Figure 7: (A) Schematic diagram of oral administration of RA (0.6 mg/Kg) in Sham- and SNI-treated mice. (B, C) Contralateral stimulus-response curves, mechanical threshold (B) and thermal latency (C) in SNI-treated mice after oral intake of RA (n = 12). PWMT: Mann-Whitney *U* test. PWTL: 2-tailed unpaired *t* test. (D) EPM in SNI-treated mice after oral intake of RA (n = 12). Time in open arms: 2-tailed unpaired *t* test. Distance in open arm and total distance: Mann-Whitney *U* test. (E) OFT in SNI-treated mice after oral administration RA (n = 12). Time in centre area: Mann-Whitney *U* test. Distance in centre area and total distance: 2-tailed unpaired *t* test. (F, G) Ipsilateral stimulus-response curve, mechanical threshold (F) and thermal latency (G) as shown after oral RA in Sham-treated mice (n = 10). 2-tailed unpaired *t* test. (H, I) Contralateral stimulus-response curve, mechanical threshold (H) and thermal latency (I) as shown after oral RA in Sham-treated mice (n = 10). PWMT: Mann-Whitney *U* test. PWTL: 2-tailed unpaired *t* test. (J) EPM in Sham-treated mice after oral RA (n =15). Distance in open arms and total distance: 2-tailed unpaired *t* test. Time in open arms: Mann-Whitney *U* test. (K) OFT after oral RA in Sham-treated mice (n = 15). Mann-Whitney *U* test. (L, M) Sucrose preference in SPT (L) and immobility in TST (M) as shown after oral RA in Sham-operated mice (n = 10). 2-tailed unpaired *t* test. Data are presented as mean ± S.E.M. $^*P < 0.05$, $^{**}P < 0.01$, $^{****}P < 0.0001$.

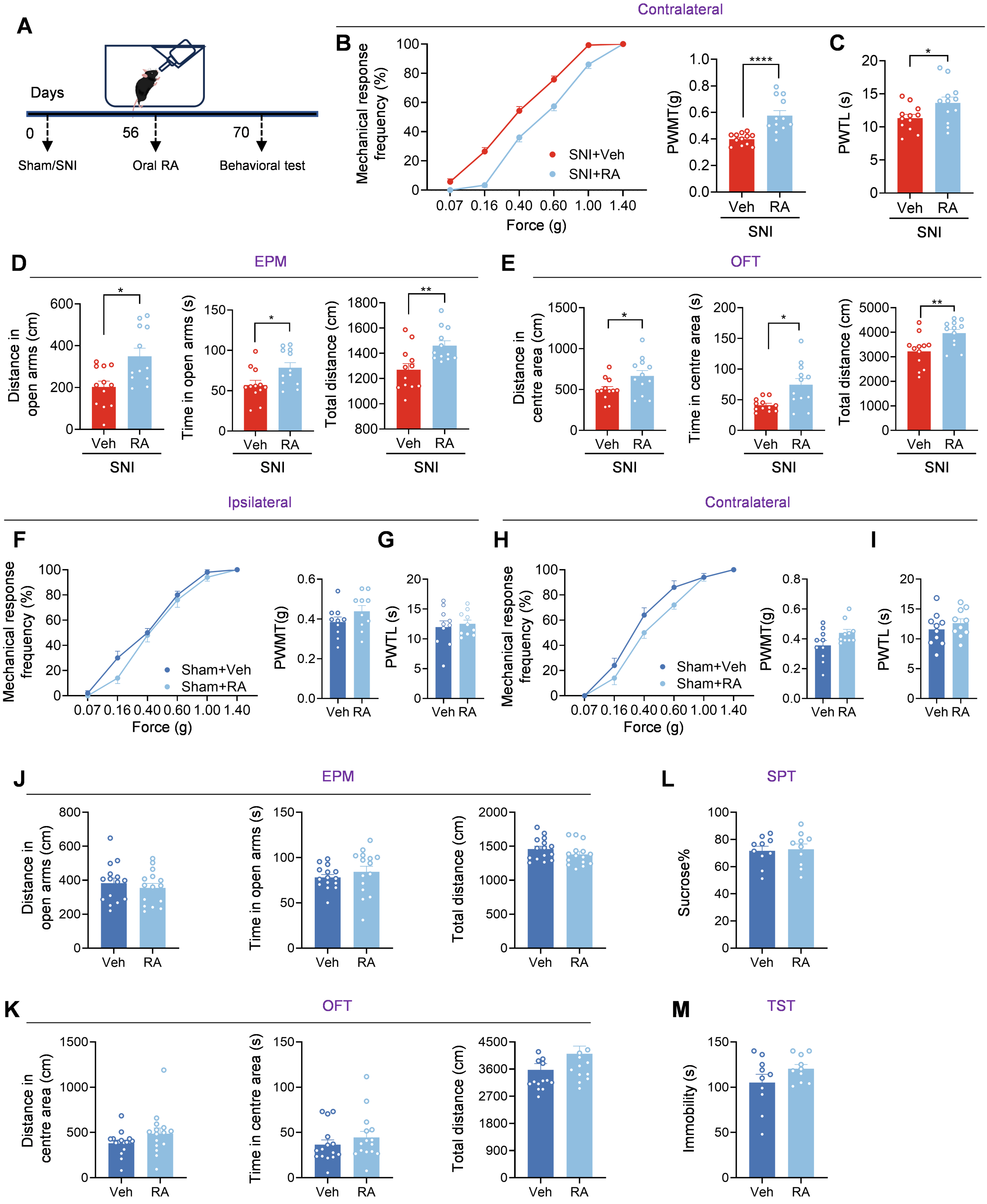

A
Days
0
56
70
Sham/SNI
Oral RA
Behavioral test
Contralateral
B
Mechanical response frequency (%)
Force (g)
SNI+Veh
SNI+RA
PWMT(g)
****
Veh RA
SNI
C
PWTL (s)
*
D
EPM
Distance in open arms (cm)
Time in open arms (s)
Total distance (cm)
E
OFT
Distance in centre area (cm)
Time in centre area (s)
Total distance (cm)
Ipsilateral
Contralateral
F
G
H
I
Sham+Veh
Sham+RA
J
EPM
L
SPT
Sucrose%
K
OFT
M
TST
Immobility (s)

Supplemental Figure 8: (A) Schematic diagram of intra-ACC injection of TLZ in Sham- and SNI-treated mice. (B, C) Contralateral stimulus-response curves, PWMT (B) and PWTL (C) in SNI-treated mice after intra-ACC injection of TLZ (n = 8-12). PWMT: Kruskal-Wallis *H* test. PWTL: 1-way ANOVA. (D) Time staying in the open arm and total distance in SNI-treated mice after intra-ACC injection of TLZ (n = 8-12). Time in open arms: 1-way ANOVA. Total distance: Kruskal-Wallis *H* test. (E) Time staying in the centre area and total distance in SNI-treated mice after intra-ACC injection of TLZ (n = 8-12). Time in centre area: Kruskal-Wallis *H* test. Total distance: 1-way ANOVA. (F, G) Ipsilateral stimulus-response curve, PWMT (F) and PWTL (G) as shown after intra-ACC injection of TLZ (5 pmol) in Sham-treated mice (n = 8). Mann-Whitney *U* test. (H, I) Contralateral stimulus-response curve, mechanical threshold (H) and thermal latency (I) in Sham-treated mice after intra-ACC injection of TLZ (5 pmol) (n = 8). PWMT: Mann-Whitney *U* test. PWTL: 2-tailed unpaired *t* test. (J) EPM in Sham-treated mice after intra-ACC injection of TLZ (5 pmol) (n = 8). Distance in open arms and total distance: 2-tailed unpaired *t* test. Time in open arms: Mann-Whitney *U* test. (K) OFT after intra-ACC injection of TLZ (5 pmol) in Sham-treated mice (n = 8). Distance and time in centre area: Mann-Whitney *U* test. Total distance: 2-tailed unpaired *t* test. (L, M) SPT (L) and TST (M) after intra-ACC injection of TLZ (5 pmol) in Sham-operated mice (n = 8). SPT: Mann-Whitney *U* test. TST: 2-tailed unpaired *t* test. Data are presented as mean ± S.E.M. *$P < 0.05$, **$P < 0.01$, ***$P < 0.001$, ****$P < 0.0001$.

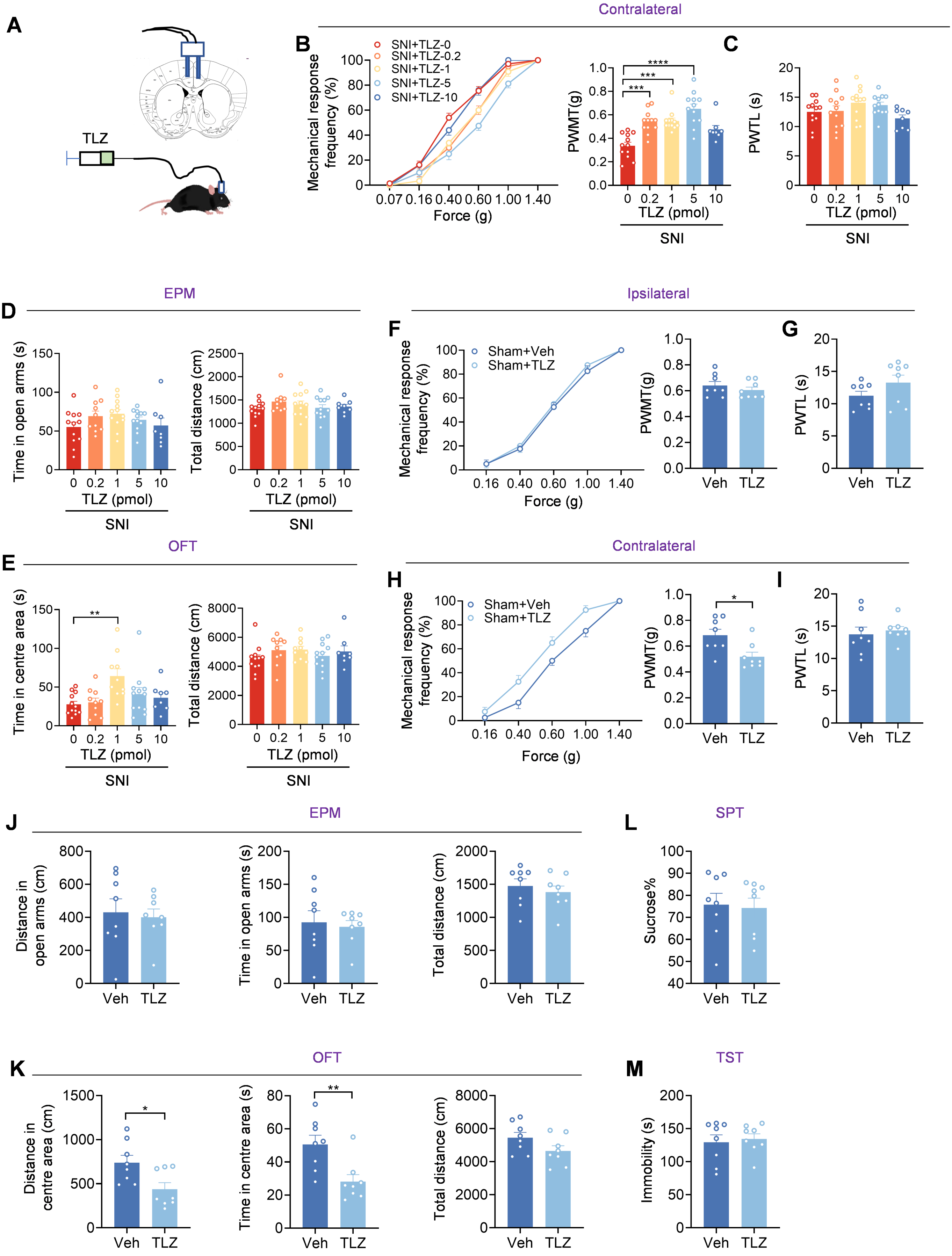
A
TLZ
B
Contralateral
SNI+TLZ-0
SNI+TLZ-0.2
SNI+TLZ-1
SNI+TLZ-5
SNI+TLZ-10
Mechanical response frequency (%)
Force (g)
C
PWMT(g)
PWTL (s)
TLZ (pmol)
SNI
D
EPM
Time in open arms (s)
Total distance (cm)
E
OFT
Time in centre area (s)
Ipsilateral
F
Sham+Veh
Sham+TLZ
G
H
I
Veh
TLZ
J
Distance in open arms (cm)
Sucrose%
L
SPT
K
Distance in centre area (cm)
M
TST
Immobility (s)

Supplemental Figure 9: (A) Schematic diagram of i.p. injection of TLZ in SNI-treated mice. (B, C) Stimulus-response curves, mechanical threshold (B) and thermal latency (C) showing i.p. injection of TLZ dose-dependently relieved contralateral SNI-induced mechanical allodynia and thermal hyperalgesia (n = 12). PWMT: Kruskal-Wallis *H* test. PWTL: 1-way ANOVA. (D) Quantitative summary showing time staying in the open arm and total distance in SNI-treated mice after i.p. injection of TLZ (n = 12). Time in open arms: Kruskal-Wallis *H* test. Total distance: 1-way ANOVA. (E) Quantitative summary showing time staying in the centre area and total distance in SNI-treated mice after i.p. injection of TLZ (n = 12). 1-way ANOVA. (F, G) Ipsilateral stimulus-response curve, mechanical threshold (F) and thermal latency (G) as shown after i.p. injection of TLZ (0.1 mg/Kg) in Sham-treated mice (n = 9-10). 2-tailed unpaired *t* test. (H, I) Contralateral stimulus-response curve, mechanical threshold (H) and thermal latency (I) as shown after i.p. injection of TLZ (0.1 mg/Kg) in Sham-treated mice (n = 9-10). 2-tailed unpaired *t* test. Data are presented as mean ± S.E.M. *$P < 0.05$, **$P < 0.01$, ****$P < 0.0001$.

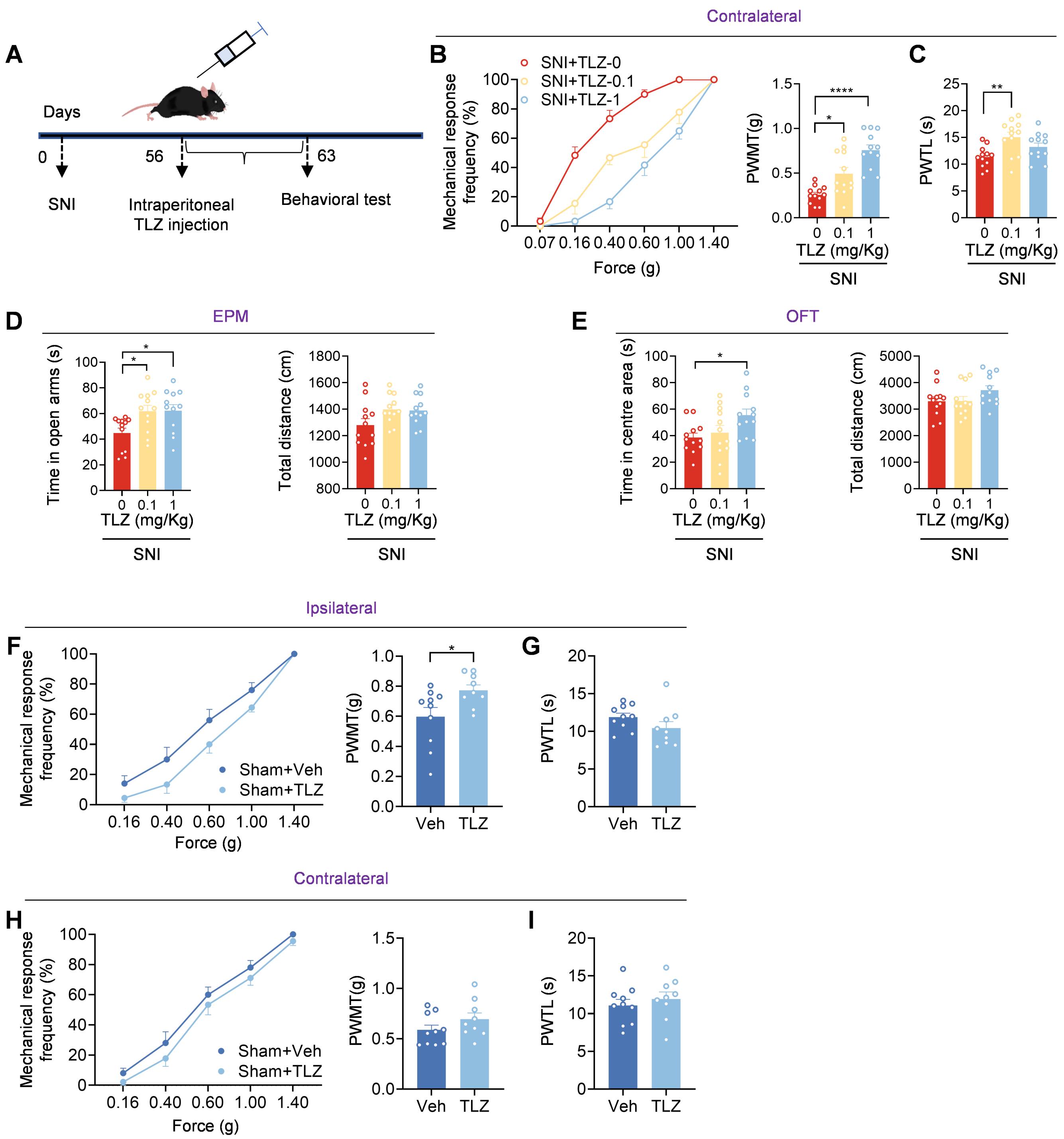

A
Days
0
56
63
SNI
Intraperitoneal
TLZ injection
Behavioral test
Contralateral
B
SNI+TLZ-0
SNI+TLZ-0.1
SNI+TLZ-1
Mechanical response frequency (%)
Force (g)
0.07 0.16 0.40 0.60 1.00 1.40
PWMT(g)
TLZ (mg/Kg)
SNI
C
PWTL (s)
TLZ (mg/Kg)
SNI
D
EPM
Time in open arms (s)
Total distance (cm)
TLZ (mg/Kg)
SNI
E
OFT
Time in centre area (s)
Total distance (cm)
TLZ (mg/Kg)
SNI
Ipsilateral
F
Mechanical response frequency (%)
Sham+Veh
Sham+TLZ
0.16 0.40 0.60 1.00 1.40
Force (g)
PWMT(g)
Veh TLZ
G
PWTL (s)
Veh TLZ
Contralateral
H
Mechanical response frequency (%)
Sham+Veh
Sham+TLZ
0.16 0.40 0.60 1.00 1.40
Force (g)
PWMT(g)
Veh TLZ
I
PWTL (s)
Veh TLZ

Supplemental Figure 10: Toxicity of RA and TLZ *in vivo*. (A-D) ELISA assay showing little changes of ALT (A), AST (B), BUN (C) and creatinine (D) level in serum from mice after oral administration of H_2O and RA for 2 consecutive weeks, separately (n = 5). 2-tailed unpaired *t* test for ALT, AST and creatinine. Mann-Whitney *U* test for BUN. (E) HE staining of lung, liver, kidney and heart as shown after oral administration of H_2O and RA for 2 consecutive weeks (n=3). Scale bar: 50 μm. (F-I) ELISA assay showing no significant change of ALT (F), AST (G), BUN (H) and creatinine (I) level in serum from mice after intraperitoneal injection of saline or TLZ 7 d (n = 5). F and I: 2-tailed unpaired *t* test. G and H: Mann-Whitney *U* test. (J) HE staining of lung, liver, kidney and heart as shown after intraperitoneal injection of saline or TLZ 7 d (n=3). Scale bar: 50 μm.

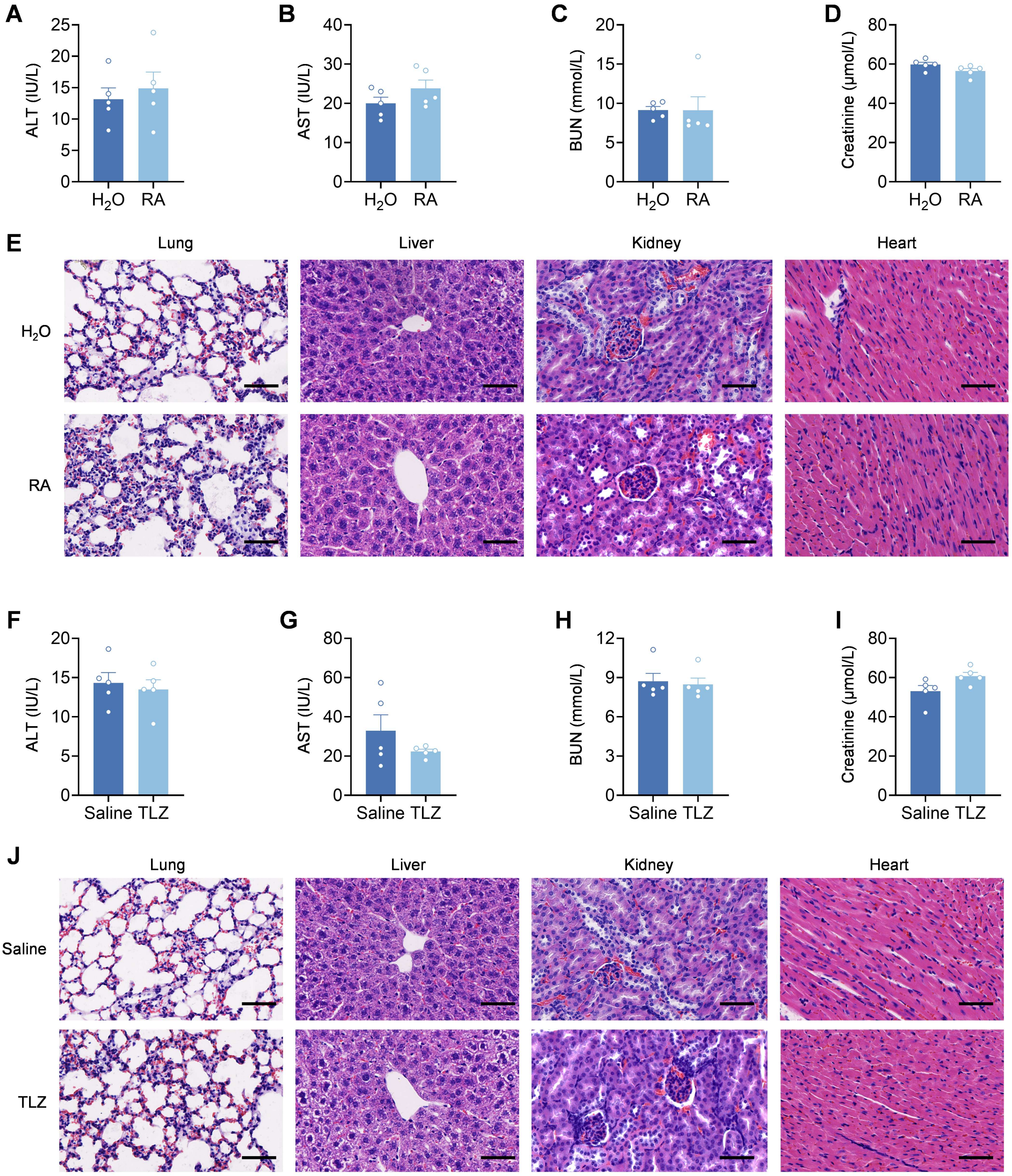

A
ALT (IU/L)
25
20
15
10
5
0
H_2O
RA
B
AST (IU/L)
40
30
20
10
0
H_2O
RA
C
BUN (mmol/L)
20
15
10
5
0
H_2O
RA
D
Creatinine (μmol/L)
80
60
40
20
0
H_2O
RA
E
Lung
Liver
Kidney
Heart
H_2O
RA
F
ALT (IU/L)
20
15
10
5
0
Saline
TLZ
G
AST (IU/L)
80
60
40
20
0
Saline
TLZ
H
BUN (mmol/L)
12
9
6
3
0
Saline
TLZ
I
Creatinine (μmol/L)
80
60
40
20
0
Saline
TLZ
J
Lung
Liver
Kidney
Heart
Saline
TLZ

Supplemental Figure 11: ALDH1A2 overexpression in ACC relieves pain hypersensitivity and depression. (A) Schematic diagram showing intra-ACC virus injection in mice. Scale bar: 1 mm. (B) Western blotting showing efficient ALDH1A2 overexpression as well as RARB and LAMB1 in ACC (n = 3). 2-tailed unpaired *t* test. (C, D) Ipsilateral stimulus-response curve and mechanical threshold as shown after ALDH1A2 overexpression in ACC in Sham- (C) and SNI- (D) treated mice (n = 16). C: 2-tailed unpaired *t* test. D: Mann-Whitney *U* test. (E) Ipsilateral thermal sensitivity was relieved by ALDH1A2 overexpression in SNI-treated state (n = 16). Kruskal-Wallis *H* test. (F, G) Contralateral stimulus-response curve and mechanical threshold as shown after ALDH1A2 overexpression in ACC in Sham- (F) and SNI- (G) treated mice (n = 16). F: Mann-Whitney *U* test. G: 2-tailed unpaired *t* test. (H) Contralateral thermal sensitivity was unaltered by ALDH1A2 overexpression in the Sham- or SNI-treated mice (n = 16). 1-way ANOVA. (I) EPM in Sham- and SNI-treated mice after overexpressing ALDH1A2 in ACC (n = 13-14). Distance in open arms and total distance: 1-way ANOVA. Time in open arms: Kruskal-Wallis *H* test. (J) OFT in Sham- and SNI-treated mice after overexpressing ALDH1A2 in ACC (n = 12-14). Kruskal-Wallis *H* test. (K, L) SPT (K) (n = 10) and TST (L) (n = 15) in mice after overexpressing ALDH1A2 in ACC. SPT: 1-way ANOVA. TST: Kruskal-Wallis *H* test. Data are presented as mean ± S.E.M. *$P < 0.05$, **$P < 0.01$, ***$P < 0.001$, ****$P < 0.0001$.

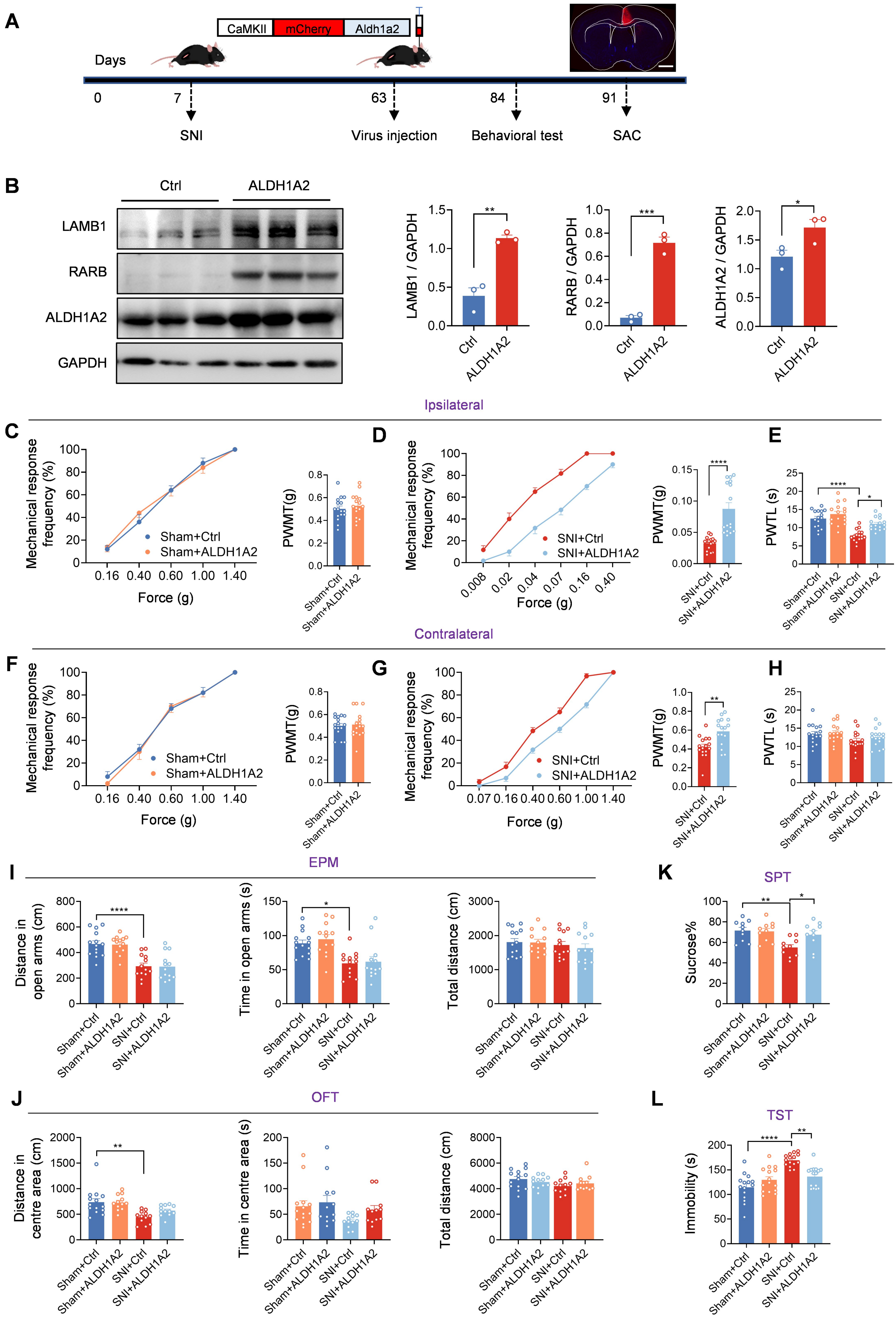

A
CaMKII
mCherry
Aldh1a2
Days
0
7
63
84
91
SNI
Virus injection
Behavioral test
SAC
B
Ctrl
ALDH1A2
LAMB1
RARB
ALDH1A2
GAPDH
LAMB1 / GAPDH
RARB / GAPDH
ALDH1A2 / GAPDH
Ipsilateral
C
Mechanical response frequency (%)
Sham+Ctrl
Sham+ALDH1A2
Force (g)
PWMT(g)
D
SNI+Ctrl
SNI+ALDH1A2
E
PWTL (s)
Contralateral
F
G
H
I
EPM
Distance in open arms (cm)
Time in open arms (s)
Total distance (cm)
K
SPT
Sucrose%
J
OFT
Distance in centre area (cm)
Time in centre area (s)
L
TST
Immobility (s)

Supplemental Figure 12: (A) Immunoblots and quantitative summary showing that RARB expression was not altered in the ACC of CORT-treated mice as compared with the control group (n = 4). 2-tailed unpaired *t* test. (B) Immunoblots and quantitative summary showing that RARB expression was unchanged in the ACC in CRS-treated mice as compared with the control group (n = 5). 2-tailed unpaired *t* test. (C) Representative immunoblots and quantitative summary showing that RARB expression was unaltered in the contralateral ACC of CFA inflammation state as compared with the control group (n = 6). 2-tailed unpaired *t* test. (D) Typical blots and quantitative summary showing that RARB expression in female mice after SNI surgery (n = 3). Kruskal-Wallis *H* test. Data are presented as mean ± S.E.M. *$P < 0.05$.

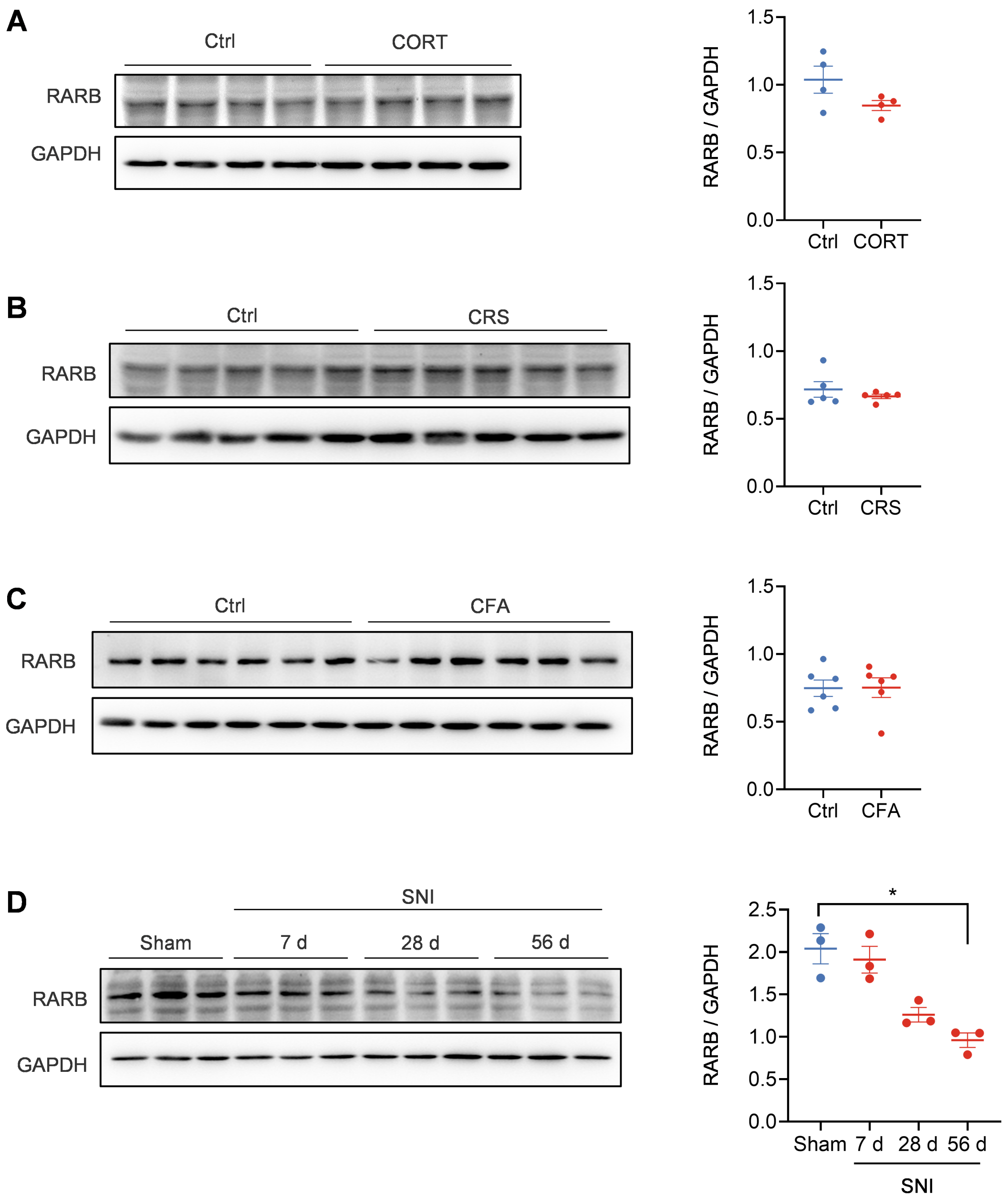

A
Ctrl
CORT
RARB
GAPDH
1.5
1.0
0.5
0.0
RARB / GAPDH
Ctrl
CORT
B
Ctrl
CRS
RARB
GAPDH
1.5
1.0
0.5
0.0
RARB / GAPDH
Ctrl
CRS
C
Ctrl
CFA
RARB
GAPDH
1.5
1.0
0.5
0.0
RARB / GAPDH
Ctrl
CFA
D
SNI
Sham
7 d
28 d
56 d
RARB
GAPDH
*
2.5
2.0
1.5
1.0
0.5
0.0
RARB / GAPDH
Sham
7 d
28 d
56 d
SNI

SUPPLEMENTAL TABLE 1: KEY RESOURCES TABLE

REAGENT or RESOURCE	SOURCE	IDENTIFIER	Dilution
Antibodies			
Rabbit anti-LAMB1	Proteintech, Wuhan, China	23498-1-AP	1:500 (WB)
Rabbit anti-RARB	Abcam, Cambridgeshire, UK	ab124701	1:1000 (WB)<br>1:200 (IF)
Rabbit anti-RARA	Proteintech, Wuhan, China	10331-1-AP	1:1000 (WB)
Rabbit anti- ALDH1A2	Proteintech, Wuhan, China	13951-1-AP	1:1000 (WB)
Mouse anti-GFP	Proteintech, Wuhan, China	66002-1-Ig	1:1000 (WB)
Mouse anti-GAPDH	Proteintech, Wuhan, China	60004-1-Ig	1:4000 (WB)
Mouse anti-NeuN	Abcam, Cambridgeshire, UK	ab104224	1:200 (IF)
Mouse anti-GFAP	Abcam, Cambridgeshire, UK	ab4648	1:200 (IF)
Goat anti-Iba1	Abcam, Cambridgeshire, UK	ab5076	1:200 (IF)
Mouse anti-CaMKII	Abcam, Cambridgeshire, UK	ab22609	1:200 (IF)
Mouse anti-GAD67	Abcam, Cambridgeshire, UK	ab26116	1:200 (IF)
Anti-rabbit IgG, HRP-linked Antibody	Cell Signaling Technology, Boston, MA, USA	7074	1:4000 (WB)
Anti-mouse IgG, HRP-linked Antibody	Cell Signaling Technology, Boston, MA, USA	7076	1:4000 (WB)
Goat anti-mouse IgG (H+L) Highly Cross-Adsorbed Secondary Antibody, Alexa Fluor 488	Invitrogen, Carlsbad, CA, USA	A11029	1:800 (IF)
Goat anti-rabbit IgG (H+L) Highly Cross-Adsorbed Secondary Antibody, Alexa Fluor 594	Invitrogen, Carlsbad, CA, USA	A32740	1:800 (IF)
Goat anti-rabbit IgG (H+L) Highly Cross-Adsorbed Secondary Antibody, Alexa Fluor 647	Invitrogen, Carlsbad, CA, USA	A32733	1:800 (IF)

Donkey anti-goat IgG (H+L) Cross-Adsorbed Secondary Antibody, Alexa Fluor 488	Invitrogen, Carlsbad, CA, USA	A32814	1:800 (IF)
Chemicals			
DAPI	Sigma-Aldrich, St. Louis, MO, USA	D9542	1 μg/ml (IF)
Retinoic acid	MCE, Monmouth Junction, NJ, USA	HY-14649	
Talarozole	MCE, Monmouth Junction, NJ, USA	HY-14531	
Corticosterone	Solarbio, Beijing, China	C9480	0.1 mg/ml
QX314	Sigma-Aldrich, St. Louis, MO, USA	L5783	5 mM
AP5	Sigma-Aldrich, St. Louis, MO, USA	A8054	50 μM
Picrotoxin	Sigma-Aldrich, St. Louis, MO, USA	R284556	100 μM
Sodium deoxycholate	Solarbio, Beijing, China	D8331	4 %
DNase I	Solarbio, Beijing, China	D8071	40 Ku/mL
Hexamethyl disilazane	Sigma-Aldrich, St. Louis, MO, USA	283134	
Complete Freund's adjuvant	Sigma-Aldrich, St. Louis, MO, USA	F5881	1 mg/ml
Tetrodotoxin	TOCRIS, Bristol, UK	1078	1 μM
Virus Strains			
rAAV2/9-CaMKIIα-mRarb-3×FLAG-P2A-mCherry-WPRE-hGH polyA	Braincase, Shenzhen, China	BC-1070	
rAAV2/9-CaMKIIα-mCherry-WPRE-hGH polyA	Braincase, Shenzhen, China	BC-0028	
rAAV2/9-CaMKIIα-mCherry-5'miR-30a-shRNA(mRarb)-3'miR-30a-WPREs	Braincase, Shenzhen, China	BC-1033	
rAAV2/9- CaMKIIα-mCherry-5'miR-30a-shRNA(Scramble)-3'miR-30a-WPREs	Braincase, Shenzhen, China	BC-1034	
rAAV2/9-CaMKIIα-GCaMP6s-WPRE-pA	Braincase, Shenzhen, China	PT-0110	

rAAV2/9-3×RARE-Mini-TK promotor-EGFP-3×Flag-SV40-puromycin	Genechem, Shanghai, China	GOSV0366178
rAAV2/9-CaMKIIα-mAldh1a2-3×FLAG-P2A-mCherry-WPREs	Braincase, Shenzhen, China	BC-1664
rAAV2/9-U6-shRNA(scramble)-CMV-EGFP-SV40pA	BrainVTA, Wuhan, China	PT-0916
rAAV2/9-U6-shRNA (Lamb1)-CMV-EGFP-SV40pA	BrainVTA, Wuhan, China	PT-2272
Plasmids		
pCDNA3.1(+)-mRarb	Genomeditech, Shanghai, China	47719
PGL3-basic-mLamb1 promoter(-1862to+84) WT	Genomeditech, Shanghai, China	47717
pCDNA3.1(+)	Genomeditech, Shanghai, China	GM-1013FL01
PGL3-basic	Genomeditech, Shanghai, China	GM-1013P001
pGMR-TK	Genomeditech, Shanghai, China	GM-0210RC
PGL3-basic-mLamb1 promoter(-1862to+84) MT(-77~-432)	Genomeditech, Shanghai, China	110301GA-P
PGL3-basic-mRarb promoter(-2000to+30) WT	Genechem, Shanghai, China	GOSE5012850
