# Supplementary material for: Cingulate retinoic acid signaling regulates neuropathic pain and comorbid anxiodepression via extracellular matrix homeostasis
Source: J Clin Invest. 2025 Jul 1;135(17):e190539. doi: 10.1172/JCI190539 (PMC12404743; doi:10.1172/JCI190539)

Figure 1C

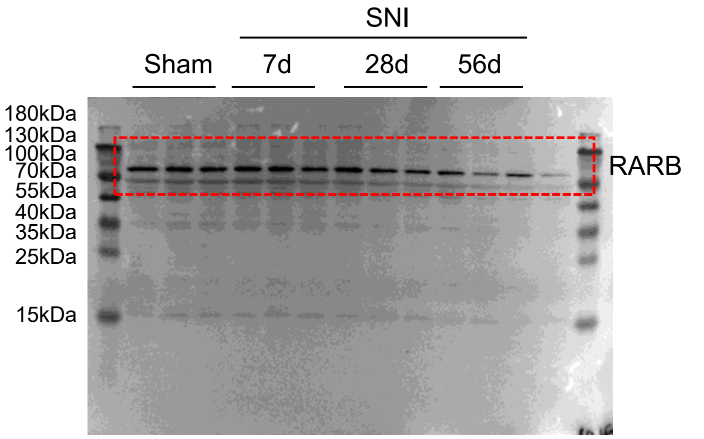
SNI
Sham
7d
28d
56d
180kDa
130kDa
100kDa
70kDa
55kDa
40kDa
35kDa
25kDa
15kDa
RARB

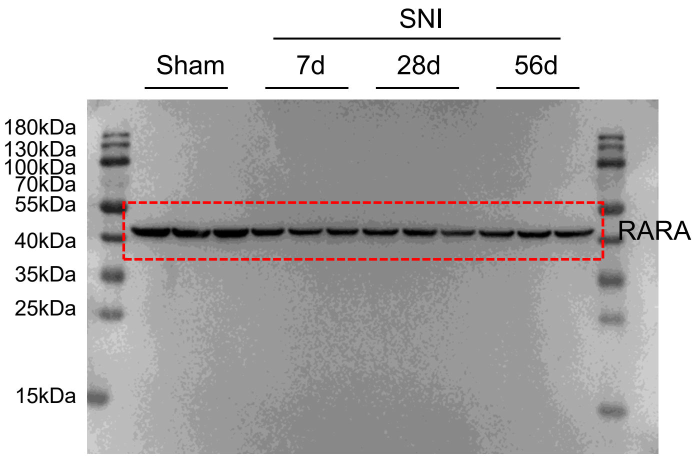
SNI
Sham
7d
28d
56d
180kDa
130kDa
100kDa
70kDa
55kDa
40kDa
35kDa
25kDa
15kDa
RARA

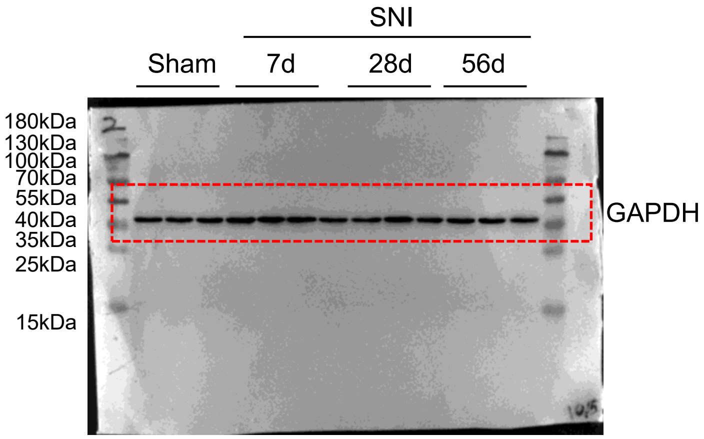
SNI
Sham
7d
28d
56d
180kDa
130kDa
100kDa
70kDa
55kDa
40kDa
35kDa
25kDa
15kDa
GAPDH

Figure 2C

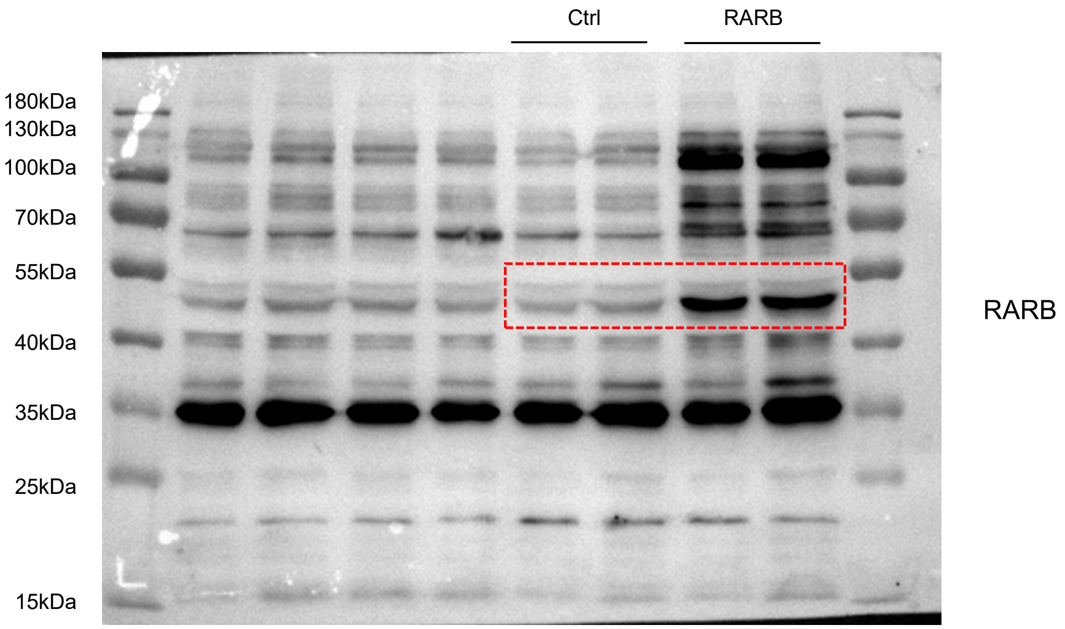

Ctrl
RARB
180kDa
130kDa
100kDa
70kDa
55kDa
40kDa
35kDa
25kDa
15kDa
RARB


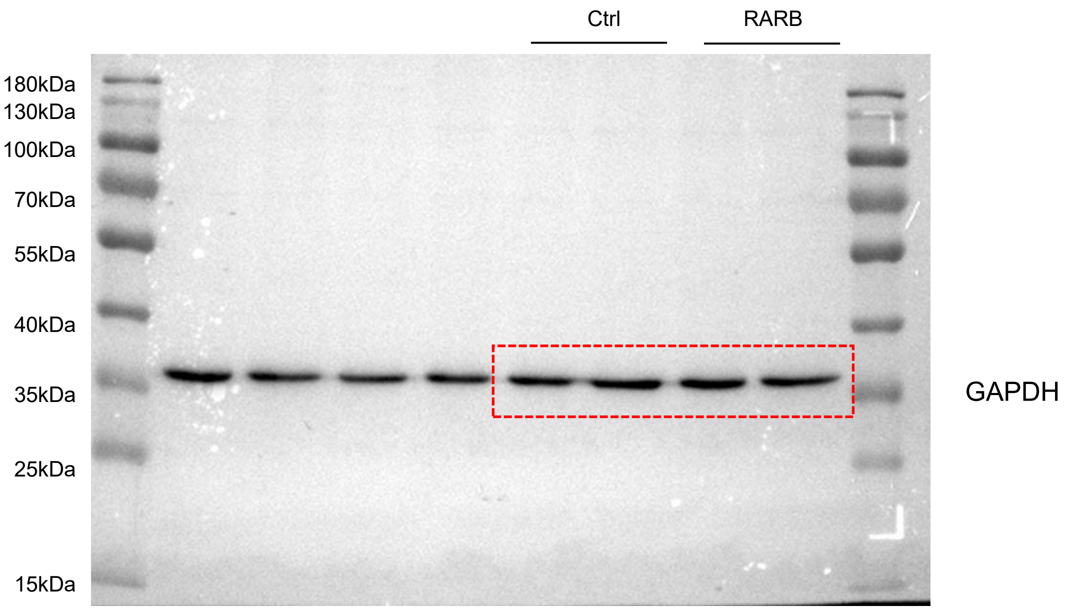

Ctrl
RARB
180kDa
130kDa
100kDa
70kDa
55kDa
40kDa
35kDa
25kDa
15kDa
GAPDH

Figure 5C

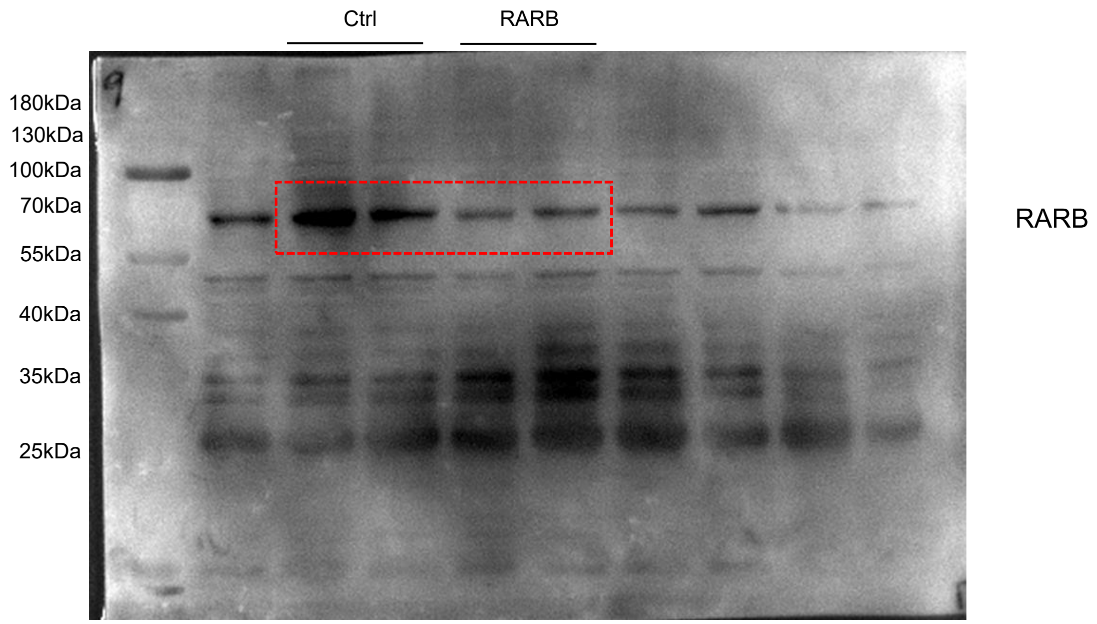
Ctrl
RARB
180kDa
130kDa
100kDa
70kDa
55kDa
40kDa
35kDa
25kDa
RARB

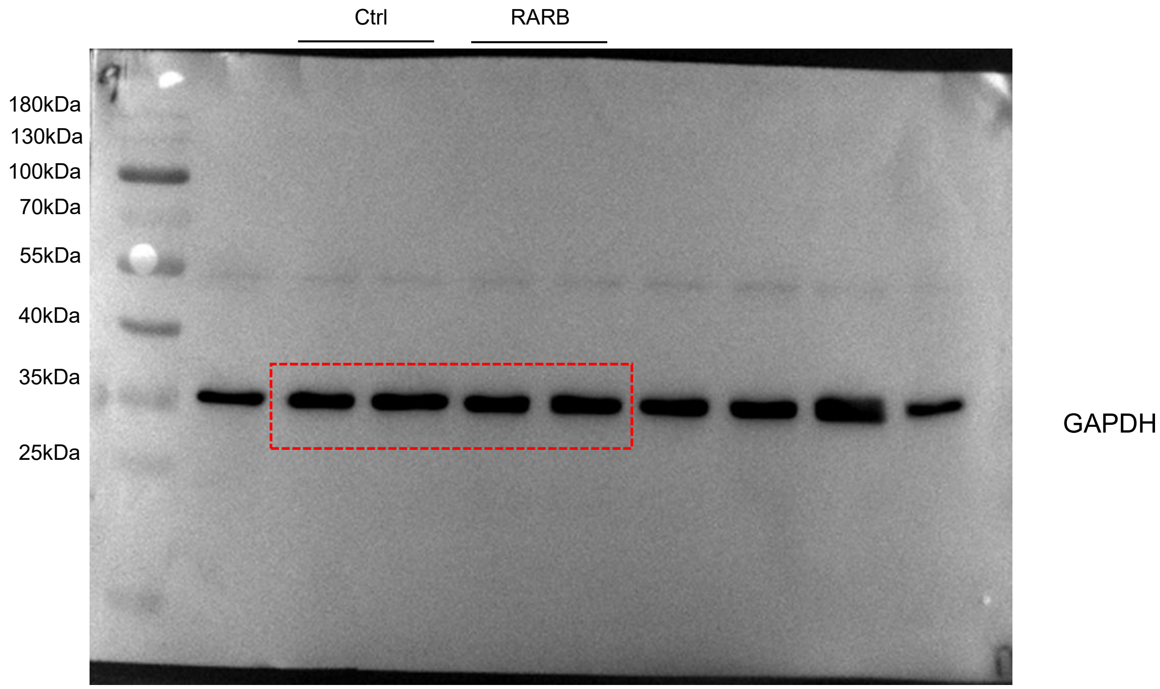
Ctrl
RARB
180kDa
130kDa
100kDa
70kDa
55kDa
40kDa
35kDa
25kDa
GAPDH

## Figure 6A

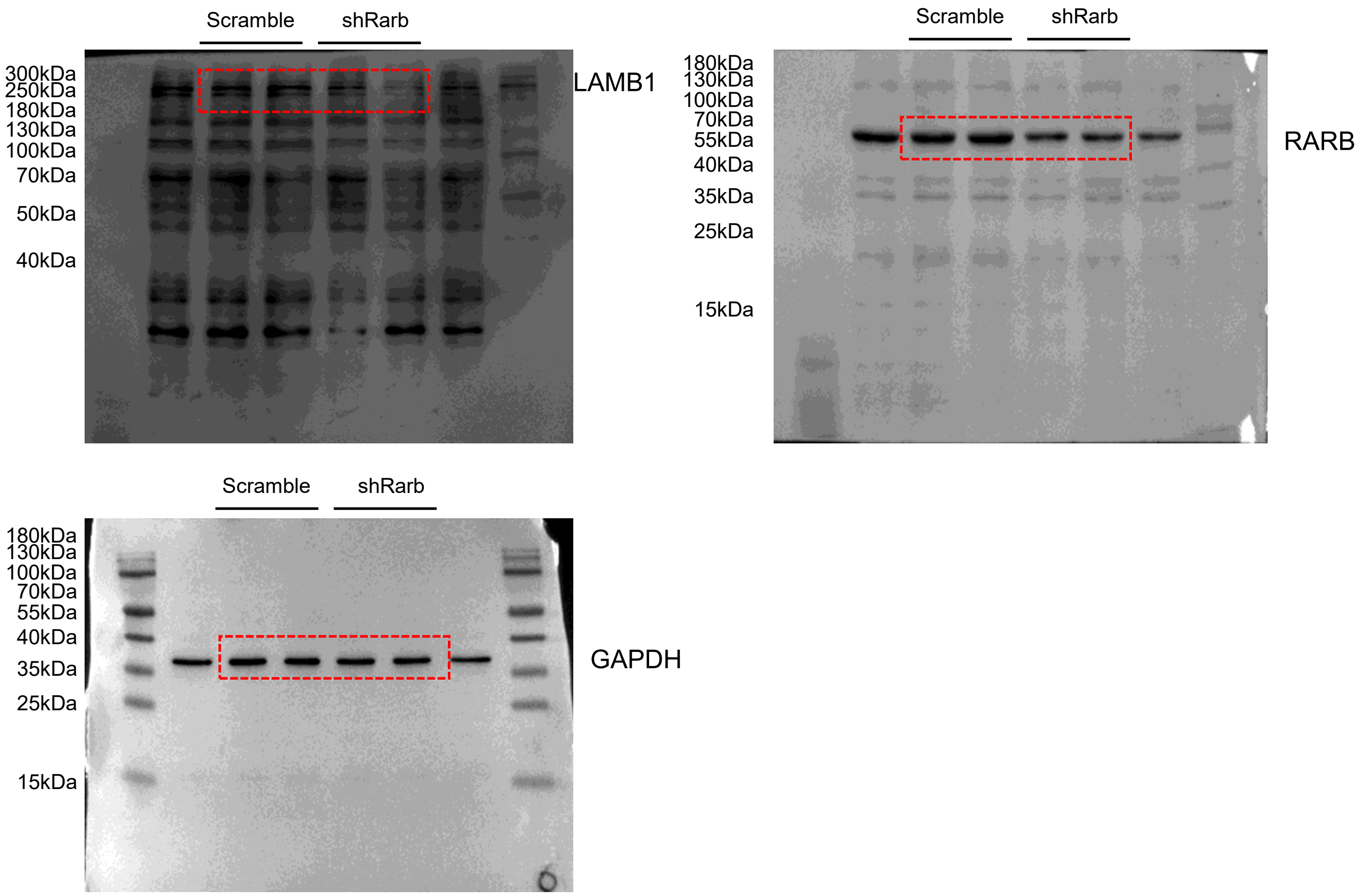


## Figure 6B

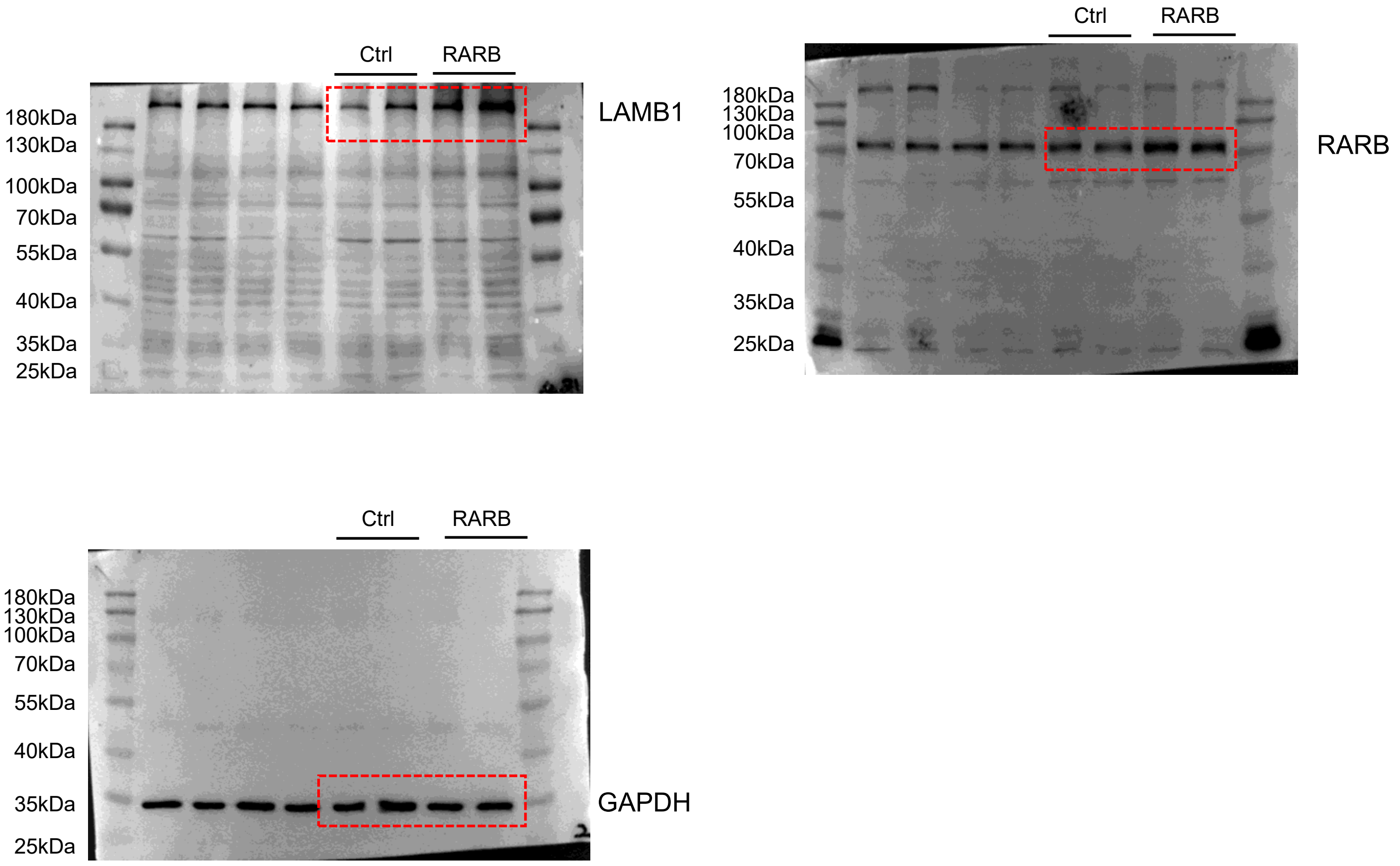

Figure 7G

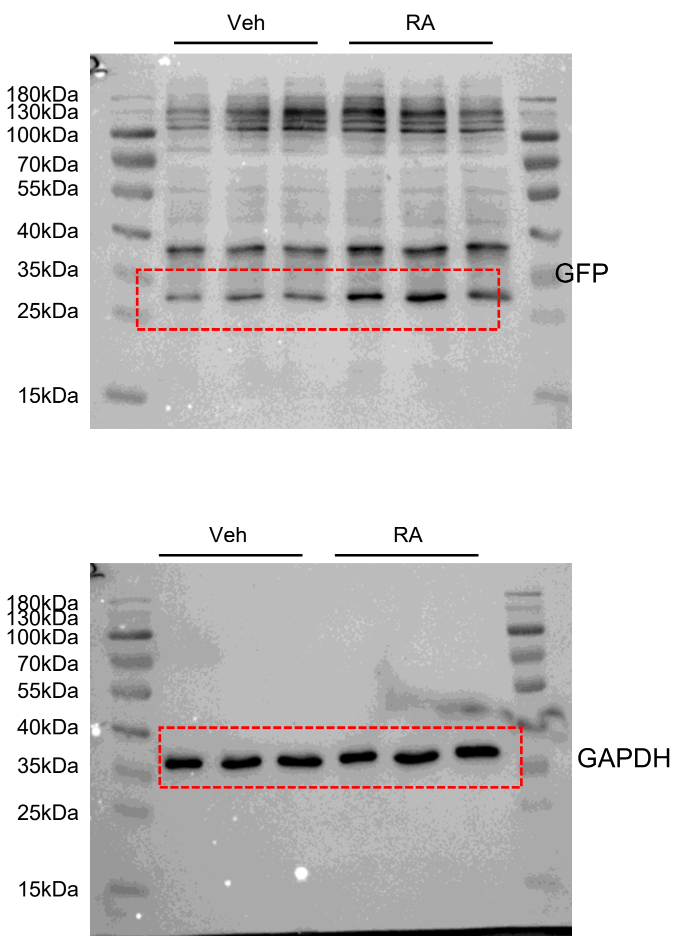
Veh
RA
180kDa
130kDa
100kDa
70kDa
55kDa
40kDa
35kDa
25kDa
15kDa
GFP
Veh
RA
180kDa
130kDa
100kDa
70kDa
55kDa
40kDa
35kDa
25kDa
15kDa
GAPDH

Figure 8B

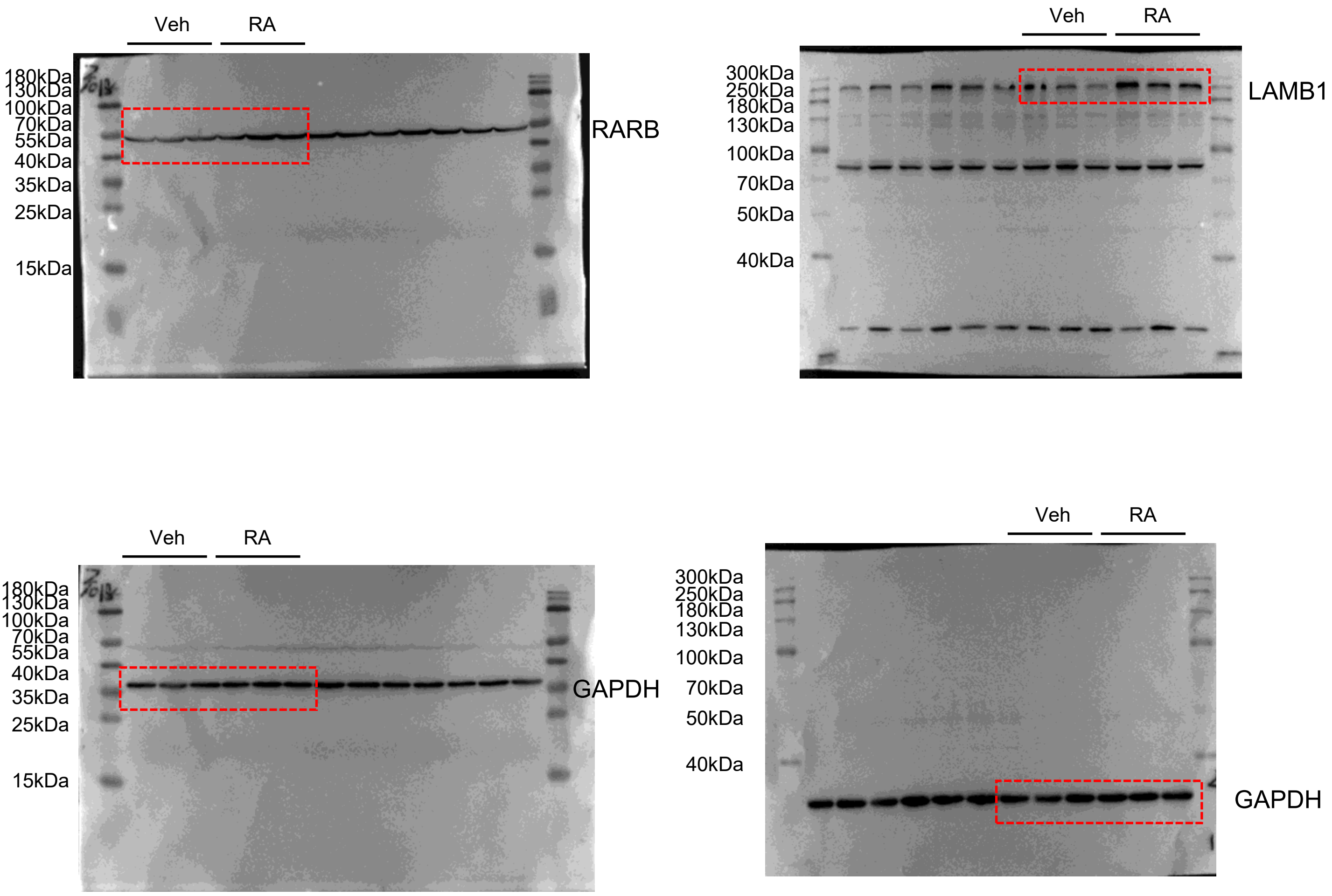
Veh
RA
180kDa
130kDa
100kDa
70kDa
55kDa
40kDa
35kDa
25kDa
15kDa
RARB
Veh
RA
300kDa
250kDa
180kDa
130kDa
100kDa
70kDa
50kDa
40kDa
LAMB1
Veh
RA
180kDa
130kDa
100kDa
70kDa
55kDa
40kDa
35kDa
25kDa
15kDa
GAPDH
Veh
RA
300kDa
250kDa
180kDa
130kDa
100kDa
70kDa
50kDa
40kDa
GAPDH

Supplementary Figure 11B

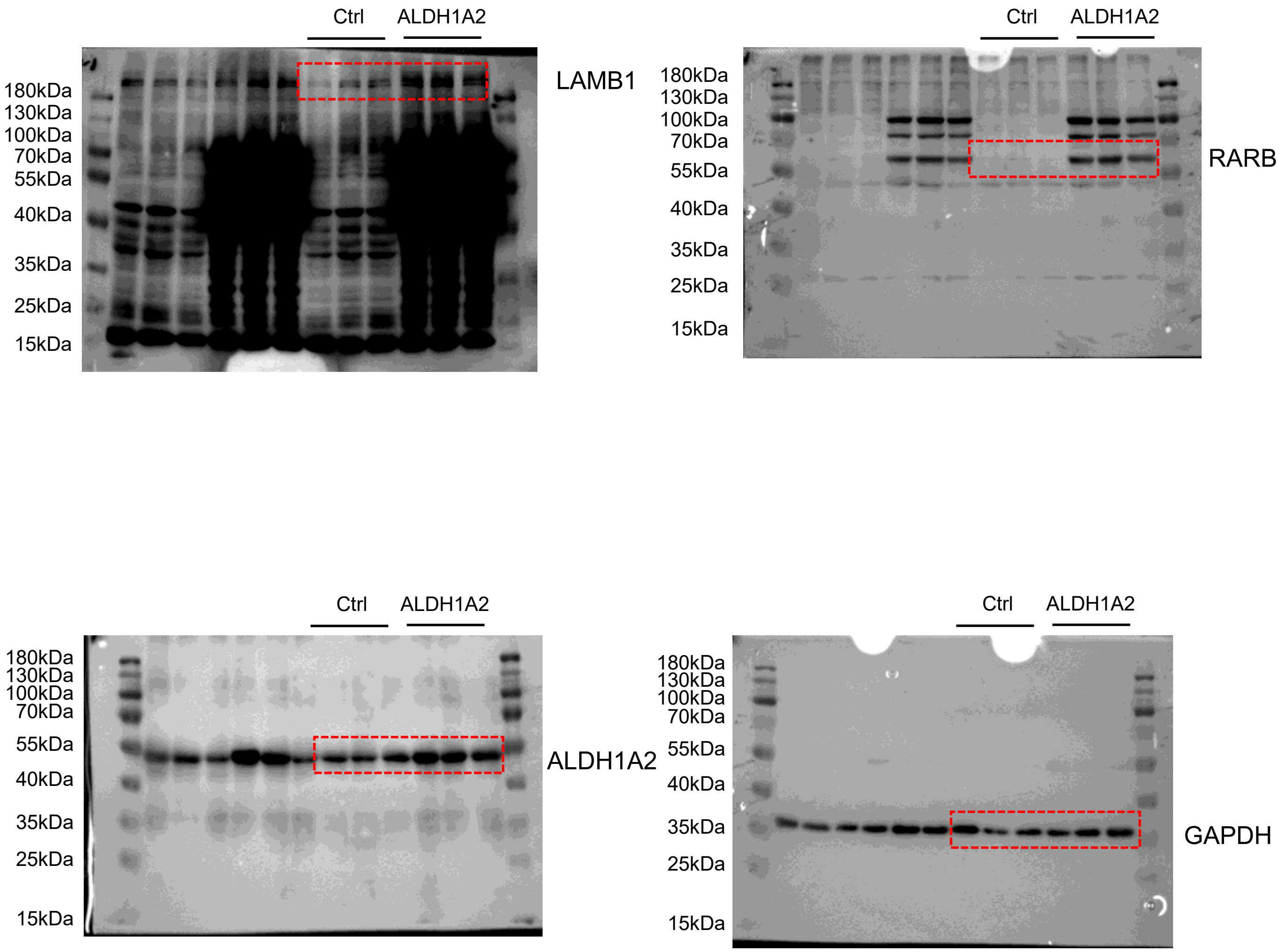

Supplementary Figure 12

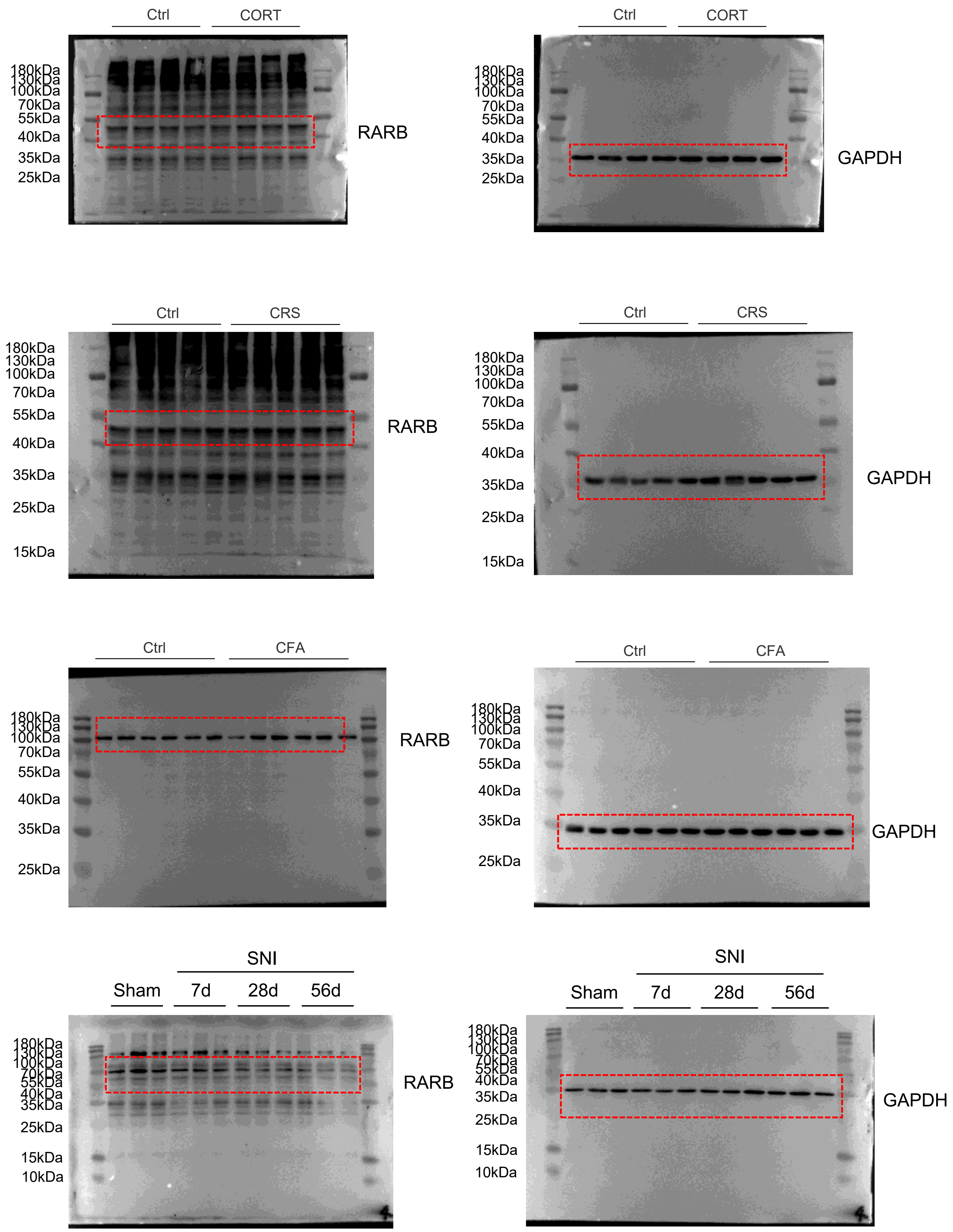

Supplement: Unedited blot and gel images [file jci-135-190539-s169.pdf]
